# Development, content validity, and reliability of an instrument to assess the commercialization of food and beverages in school canteens in Brazil

**DOI:** 10.1590/1980-549720260015

**Published:** 2026-04-03

**Authors:** Luiza Delazari Borges, Letícia Ferreira Tavares, Ariene Silva do Carmo, Letícia de Oliveira Cardoso, Raquel Canuto, Paulo César Pereira de Castro, Sabrina Gomes Ferreira Clark, Luísa Arantes Vilela, Larissa Loures Mendes

**Affiliations:** I Universidade Federal de Minas Gerais - Belo Horizonte (MG), Brazil.; II Universidade Federal do Rio de Janeiro - Rio de Janeiro (RJ), Brazil.; III Universidade Federal de Ouro Preto - Ouro Preto (MG), Brazil.; IV Fundação Oswaldo Cruz, Sergio Arouca National School of Public Health - Rio de Janeiro (RJ), Brazil.; V Universidade Federal do Rio Grande do Sul - Porto Alegre (RS), Brazil.; VI Universidade Federal de Pernambuco - Recife (PE), Brazil.

**Keywords:** School meals, Environment, Reliability and validity, Food social space

## Abstract

**Objectives:**

To develop, verify content validity, and evaluate the reliability of an instrument for assessing the commercialization of food and beverages in school canteens.

**Methods:**

The preliminary version of the instrument was developed following a literature review, identification of food environment dimensions, item development, and structuring of the instrument, which was divided into three sections: identification and characterization of canteens, commercialized foods, and food advertising. The instrument was evaluated by experts, and the content validity index was calculated for each item (deemed satisfactory if >0.80). Reliability was assessed in a sample of canteens using inter-rater and intra-rater tests. Values of kappa, prevalence-adjusted bias-adjusted kappa, and intraclass correlation coefficient were calculated.

**Results:**

The instrument included 945 items. The average content validity index score of the instrument, considering the evaluated aspects (relevance, clarity of item wording, and clarity of response options), was 0.95. Experts’ suggestions were reviewed, and items were added, removed, or modified when appropriate. Regarding reliability, 95.2% of the total analyzed items showed excellent, very good, or good agreement (≥0.6).

**Conclusion:**

The instrument proved to be reliable for measuring the food environment in school canteens in the Brazilian context, showing satisfactory values for both content validity and reliability. Thus, the instrument contributes to advancing the assessment of food commercialization in canteens, an important aspect of the school food environment.

## INTRODUCTION

The environments where people are inserted influence food choices and food consumption in various ways, through a set of elements that encompass physical, economic, political, and sociocultural aspects of the environments^1^ ^,^ ^2^ ^,^ ^3^. Thus, the school food environment is defined as spaces, infrastructure, and conditions, inside and around schools, in which food is available and can be obtained, purchased, or consumed: canteens, cafeterias, vending machines, and street vendors, as well as any facility where food is commercialized. It also includes information, promotion (marketing, advertising, brands, labels, packaging) and pricing for food^4^.

In this context, school canteens stand out as spaces within the schools destined for the commercialization of food and beverages for the school community^5^. They influence food choices, being accessible to 31.4% of students from public schools and 88% of students from private institutions in Brazil, according to the 2019 National Survey of School Health (*Pesquisa Nacional de Saúde do Escolar -* PeNSE)^6^.

The commercialization of unhealthy foods in schools is associated with higher consumption of these foods among students^7^ ^,^ ^8^ ^,^ ^9^. In Brazil, an association was verified between the regular purchase of snacks in canteens and a higher chance of sugary drinks consumption by adolescents^10^ and the high prevalence of ultra-processed food commercialization in private schools^11^.

Taking this into consideration, the importance of instruments with good psychometric properties to evaluate food commercialization in school canteens is highlighted. Studies that validate or verify the reliability of these instruments are still scarce. Authors of studies conducted in Australia^12^ ^,^ ^13^ ^,^ ^14^ ^,^ ^15^, New Zealand^16^, South Africa^17^ ^,^ ^18^, and Norway^19^ assessed the validity and/or reliability of instruments applied to different aspects of the school environment. In Brazil, there are no validated instruments to accurately and reliably measure food commercialization in school canteens, making it difficult to compare studies^20^ and limiting the advancement of scientific knowledge^21^.

In view of this gap and the impact of the school food environment on food consumption of children and adolescents, in the present study, we aimed to develop, verify content validity, and evaluate the reliability of an instrument to assess the commercialization of food and beverages in these spaces.

## METHODS

### Developing the instrument

The instrument was developed within the context of the study Food Commercialization in Brazilian Schools (*Comercialização de Alimentos em Escolas Brasileiras* - Caeb), whose main objective was to evaluate the commercialization of food and beverages in private schools in Brazilian capitals^22^.

The first stage for developing the instrument was the review of existing instruments that evaluate the school food environment and encompass dimensions or items applicable to the Brazilian context. The review was conducted by two researchers in the Medical Literature Analysis and Retrieval System Online (MEDLINE, via the United States National Library of Medicine - PubMed) and the Scientific Electronic Library Online (SciELO) databases in 2022. The preliminary versions of the instrument were elaborated based on the identification of the dimensions, formulation of the items, and structuring of the instrument.

Among the dimensions identified are availability (which includes the variety of available foods), affordability (price), convenience (payment methods, opening hours)^23^, nutritional information^1^, food promotion (advertising)^1^ ^,^ ^3^ ^,^ ^24^ ^,^ ^25^ ^,^ ^26^, infrastructure for preparing meals, governance^27^, and policies^3^. The instrument was elaborated based on the following instruments: “Restaurant Observation Tool: ESAO-restaurants”^28^; “Food environment audit instrument based on the new food classification of the Dietary Guidelines (NOVA) - AUDIT-NOVA”^29^; “University food environment assessment instrument”^30^; the instrument of PeNSE^6^; and the instrument of the project “Food security in school canteens in the municipalities of the territory of citizenship of Noroeste Colonial/RS”^31^. Food-related items were elaborated based on the recommendations of the Dietary Guidelines for the Brazilian Population^32^ and the “University food environment assessment instrument”^30^.

The instrument was organized into two sections: Section 1, intended for the identification and characterization of canteens; and Section 2, focused on the commercialized foods. After developing the preliminary versions, experts evaluated the instrument. This process consists in the prototypical phase, essential for the refinement of the initial drafts and the creation of an appropriate instrument for the proposed measurement^21^.

The instrument was developed for use by researchers, civil society, the government, and other stakeholders, aiming to evaluate the commercialization of food in school canteens focusing on adequate and healthy diet. To this end, it is imperative to understand how canteens operate, as their organization and operation influence the availability of food.

The instrument does not generate a single score or a general classification, as each dimension can be analyzed separately according to the evaluated aspect. However, based on the results of Section 2 concerning the availability of food, the healthiness index has been calculated^33^. This index is obtained by the sum of the availability of fresh, minimally processed, processed foods, and culinary preparations based on these foods and the non-availability of ultra-processed foods and culinary preparations based on these foods. The final value ranges from 0 to 100, indicating greater adequacy the closer to 100^33^.

### Experts’ evaluation

It is recommended that five to 20 experts evaluate the instrument^34^ ^,^ ^35^. In this study, 12 researchers in the area of nutrition and linked to different research institutions, specialized in Epidemiology, Public Health, Food Environments, Collective Eating, or Gastronomy, were invited to participate in the evaluation. All of them had experience in the study topics and/or in the development of instruments.

The preliminary version was analyzed at a single moment regarding the relevance of the items for the study objectives, clarity of writing, and absence of ambiguity, based on a four-point Likert scale, in which: 1=nonequivalent item; 2=item requires major review to evaluate equivalence; 3=equivalent item, requires minor changes; and 4=absolutely equivalent item^36^. The evaluators wrote down comments/suggestions. This stage, with a qualitative approach, integrates the prototypical phase of the elaboration of the instrument^21^.

### Pretest

The pretest was carried out in a school with characteristics similar to the schools of the Caeb study sample, with the printed instrument, and was performed by a qualified researcher.

### Reliability of the instrument

The reliability of the instrument was evaluated in a convenience sample composed of public and private schools of a capital and a municipality of its metropolitan region. The estimate of the sample of evaluated canteens (n=13) was performed based on the criteria proposed by Hulley et al.^37^ for calculating the correlation coefficient. A mean correlation of 0.70 was considered, which is classified as good agreement according to the criteria established by Byrt^38^. A 5% significance level and a test power of 80% were adopted. A total of 28 canteens were invited to participate in the study.

The collection took place between September and December 2023 via the Collect Box^39^ application and was applied by two qualified researchers with experience in school food environment. The canteens were evaluated three times^40^ ^,^ ^41^, namely: by the two researchers on the same day, with an interval of up to 30 minutes (inter-rater test), and by one of them once again after 21 days, on average (intra-rater test).

The collection combined different strategies: some pieces of information were obtained from an interview with managers or canteen employees, while others were obtained from direct observation (audit). To ensure the standardization of procedures, the collection handbook was used, also available as supplementary material.

### Data analysis

After obtaining the experts’ feedback, the Content Validity Index (CVI) was applied for quantitative evaluation^42^. CVI measures the proportion of experts who agree on aspects concerning the items^43^ based on the aforementioned four-point Likert scale^36^. Items rated 1 or 2 should be reviewed or excluded. The CVI of each item is calculated by the ratio between answers 3 and 4 and the total answers, and an index of ≥0.80 is acceptable, preferably >0.90^44^.

For reliability, categorical variables were analyzed by percentage agreement (PA^45^), kappa and prevalence-adjusted bias-adjusted kappa (PABAK^46^), and continuous variables (variety, size, and prices) by the Intraclass Correlation Coefficient (ICC^47^). The PABAK was included as a complementary measure for correcting distortions in situations of extreme prevalence, conferring greater robustness to the interpretation of agreement. The values of PA, kappa, PABAK, and ICC were classified according to the criterion of Byrt^38^ as: excellent agreement (≥0.92), very good agreement (0.80 to 0.91), good agreement (0.60 to 0.79), reasonable agreement (0.40 to 0.59), superficial agreement (0.20 to 0.39), poor agreement (0.00 to 0.19), and no agreement (<0.00). Kappa and PABAK values were not calculated for items that were not commercialized in any canteen, as the statistics could not be computed due to absence of one or more levels in the cross-reference tables.

The analyses were performed using the Statistical Package for the Social Sciences (SPSS 21)^48^ software and the Software for Statistics and Data Science (Stata/SE 15)^49^. This study complied with the ethical precepts of the Declaration of Helsinki and Resolutions No. 466/2012 and 510/2016 of the National Council of Health, and was approved by the Research Ethics Committee of Universidade Federal de Minas Gerais (Opinion No. 5.240.459). The study participants were experts and managers of canteens who consented to participate and registered their acceptance in the Informed Consent Form.

## Data Availability Statement:

The entire dataset that supports the results of this study was published in the article and in the “Supplementary Material” section.

## RESULTS

### Experts’ evaluation

83.3% (n=10) of the invited experts participated in content validity. The agreement was satisfactory, with a CVI mean of 0.9. The averages per section are presented in Table 1 of the Supplementary Material.

Based on the evaluation, the items were reviewed and adjusted according to the relevant suggestions, discussed by the study coordinators. The main changes were: inclusion of the question about the existence of the sales strategy, present in the dimension “food promotion”; inclusion of the item on the existence of meal offer and its price in the canteen; exclusion of four items related to the canteen’s identification; and reorganization of the size and price of foods and beverages - previously, the goal was to know the single size (when there was only one food/drink option), minimum and maximum, as well as the single price (when there was only one food/drink option), minimum and maximum. The final version included the size of the lowest-price food and the lowest price. The dimension of food advertising was reorganized and became the goal of the third section.

Experts recommended the elaboration of a comprehensive collection handbook, with guidance on the measurement of the variety, unit of measurement (weight, volume, or unit), and examples for the classification of foods as to their processing degree (according to the data collection handbook). The changes are described in Table 1 of the Supplementary Material.

### Pretest

According to the pretest, the items were understood and properly filled out, without the need for changes. The sequence of the sections proved to be pertinent, in addition to the content. Hence, the instrument was programmed in an application, with items dynamically configured and conditioned to previous responses, which conferred greater practicality and reduced typing errors.

### Final version of the instrument

The final version of the instrument considered the following dimensions: food and nutritional information, food infrastructure, convenience, affordability, availability, variety, and promotion. It comprised 945 items distributed in three sections:

1. Identification and characterization of canteens;
2. Commercialization, variety, size, value of the lowest price, and sales strategy for foods, beverages, and culinary preparations; and
3. Presence of advertising of these items.

The first section was composed of 95 items related to: administrative aspects, presence of nutritionist, type of meals, place of preparation, availability of menus and nutritional information, structure for consumption, commercialization of preparations for special diets (for example: allergies, intolerances, diabetes), availability of condiments, food criteria and prohibitions, incentives from suppliers, educational actions and materials on healthy diet, food price and sales strategies carried out by other members of the school community outside the canteen.

The second section was composed of 300 items encompassing the presence, variety, size of the lowest-price food, lowest price, and sales strategies (such as combos or promotions) for 50 foods and beverages. Of these, 21 are fresh, minimally-processed, or processed foods and culinary preparations based on these foods, and 29 are ultra-processed foods and culinary preparations based on these foods.

The third and last section concerns the possible marketing communication strategies of the 50 foods and beverages commercialized, comprising 550 items. 11 types of advertising strategies, such as banners, clothing, replicas, menus, among others, are verified. The complete instrument is available in the Supplementary Material.

### Reliability of the instrument

The collection included 14 canteens, with average interview duration of 30 minutes in private schools and 20 minutes in public schools. The instrument was reapplied, on average, after 21 days, with an interval of 19 to 24 days. In section 1, we could not calculate kappa*,* PABAK, and ICC for 33 items due to the absence of one or more levels in the cross-reference tables, given the low number of occurrences. The same occurred in section 2, with 124 items. In addition, 50 items were not included in the sample, because ten foods from the instrument were not available in the canteens, making it impossible to evaluate variety, size, price, combos, and promotions. In section 3, no item allowed us the calculation of these measurements, but the percentage of agreement in the inter-rater and intra-rater test was excellent (100%). Thus, we evaluated the reliability of 188 items in total.

In the overall performance, 95.2% of the total items analyzed (n=188) showed excellent, very good, or good percentage agreement. In the inter-rater test, considering the analyses for categorical variables, among the 112 items evaluated, 90.1% had excellent agreement and 9.9% had very good or good agreement for kappa (ranging from 0.75 to 1.00). For the PABAK, 89.3% of the items had excellent agreement and 9.8%, very good or good agreement. We observed null agreement in only 0.9% of the items, specifically in the PABAK analysis in the item on commercialization of 100% fruit juice in box, can, or bottle (ranging from -0.28 to 1.00) (Table 1). According to the ICC calculated for the items “number of clients served per day” and for the variety, size, and price of foods/meals in the inter-rater analysis, we verified a variation from 0.0 to 1.0 (92.1%≥0.92; 2.6% between 0.6 and 0.91; 1.3% between 0.2 and 0.39 and 3.9%<0.0). Of the total items evaluated (n=76), 92.1% presented excellent agreement and 2.6%, very good or good agreement (Table 2). Only 1.3% of the items showed superficial agreement (pizza variety with ultra-processed filling) and 3.9% of the items showed no agreement (variety of fruit candies; size of fresh fruit, and variety of sandwich with ultra-processed filling).

**Table 1.** Inter-rater reliability (kappa and prevalence-adjusted bias-adjusted kappa) of sections of the instrument for evaluating the commercialization of food and beverages in school canteens.

Sections	Number of items considered for calculating kappa*	% of items according to agreement (kappa)^†^			% of items according to agreement (PABAK)^‡^			
		E	VG	G	E	VG	G	N
Section 1: Identification and characterization of canteens	59	93.2	5.1	1.7	93.2	6.8	-	-
Section 2: Foods commercialized in canteens	53	86.8	9.4	3.7	84.9	11.3	1.8	1.8

*Of the 345 items that compose the instrument (section 1=95 items and section 2=250 items), 123 items refer to quantitative variables (variety, size, and price), not applying the kappa and PABAK calculation. Of the 222 items for which this calculation would be applied, in 110, the statistics could not be computed due to absence of one or more levels in the cross-reference tables (33 in section 1 and 77 in section 2); ^†^the categories “reasonable,” “superficial,” “poor,” and “null” were not found; ^‡^the categories “reasonable,” “superficial,” and “poor” were not found.

E: Excellent; VG: Very good; G: Good; N: Null.

**Table 2.** Inter-rater and intra-rater reliability (intraclass correlation coefficient) of the sections of the instrument for evaluating the commercialization of food and beverages in school canteens.

Sections	Number of items considered for calculating ICC*	% of items according to ICC Inter-rater test^†^					Number of items considered for calculating ICC*	% of items according to ICC Intra-rater test^‡^	
		E	VG	G	S	N		E	G
Section 1: Identification and characterization of canteens	3	100	0.0	0.0	0.0	0.0	3	100	0.0
Section 2: Foods commercialized in canteens	73	91.7	1.3	1.3	1.3	4.1	73	98.6	1.4

*In section 2, of the 120 items for which this calculation would be applied, in 47 of them the statistics could not be computed due to absence of one or more levels in the cross-reference tables; ^†^the “reasonable” category was not found; ^‡^the categories “very good,” “reasonable,” “superficial,” “poor,” and “null” were not found. ICC: Intraclass Correlation Coefficient; E: Excellent; VG: Very good; G: Good; S: Superficial; N: null.

In the intra-rater test, for the 76 items in which ICC was calculated, 97.3% presented excellent agreement, 2.7% presented very good and good agreement (ranging from 0.61 to 1.00), and no item had reasonable, superficial, poor, or null agreement (Table 2). Considering the analyses with categorical variables, of the total 112 items evaluated for kappa*,* 95.5% presented excellent agreement (≥0.92); 3.6%, very good and good agreement (ICC between 0.6 and 0.91); and 0.9%, no agreement (ICC<0.0). With regard to PABAK, 94.6% of the items were found to have excellent agreement and 5.4%, very good or good agreement, ranging from 0.8 to 1.0 (Table 3). Considering the kappa*,* we observed no agreement in 0.9% of the items, specifically in the item that evaluates permission according to municipal or state legislation as a criterion for food commercialization. Evaluation details can be seen in Tables 2, 3, and 4 of the Supplementary Material.

**Table 3.** Intra-rater reliability (kappa and prevalence-adjusted bias-adjusted kappa) of the sections of the instrument for evaluating the commercialization of food and beverages in school canteens.

Sections	Number of items considered for calculating kappa*	% of items according to agreement (kappa)^†^				% of items according to agreement (PABAK)^‡^	
		E	VG	G	P	E	VG
Section 1: Identification and characterization of canteens	59	96.6	1.7		1.7	96.6	3.4
Section 2: Foods commercialized in canteens	53	94.3	-	5.7		92.4	7.6

*Of the 345 items that compose the instrument (section 1=95 items and section 2=250 items), 123 items refer to quantitative variables (variety, size, and price), not applying the kappa and PABAK calculation. Of the 222 items for which this calculation would be applied, in 110 the statistics could not be computed due to absence of one or more levels in the cross-reference tables (33 in section 1 and 77 in section 2); ^†^the categories “reasonable,” “superficial,” and “null” were not found; ^‡^the categories “good,” “reasonable,” “superficial,” “poor,” and “null” were not found. E: Excellent; VG: Very good; G: Good; P: Poor.

## DISCUSSION

In this study, we developed, verified the content validity and the reliability of an instrument to evaluate the commercialization of food and beverages in school canteens. In the studied contexts, the instrument presented satisfactory content validity and reliability, and can be used in studies on food commercialization in school canteens.

The identification and characterization of canteens allows us to trace the profile of these establishments and understand the foods commercialized in this important organizational space. Authors who develop instruments to evaluate food environments organize items thoroughly, ensuring that each aspect to be evaluated is identified and that the dimensions of the food environment are clearly defined^28^ ^,^ ^29^ ^,^ ^30^. The foods present in the instrument were selected based on national research and the foods most frequently commercialized in canteens^6^ ^,^ ^50^ ^,^ ^51^, taking into account the NOVA classification^52^ and the current guidelines of the Dietary Guidelines for the Brazilian Population^32^. Information on variety, price, sales strategies, and advertising of commercialized foods allow a detailed assessment of the school food environment^4^ ^,^ ^53^, analyzing the obstacles and enablers that children and adolescents face when making decisions in these spaces.

Although over 90% of the items presented satisfactory reliability, a few items were poorly evaluated. Among them, we highlight those related to the commercialization of 100% juice in box, can, or bottle; the variety of pizzas with ultra-processed filling; the variety of fruit candies; the size of fresh fruit; the variety of sandwiches with ultra-processed filling; and to the criterion of permission to commercialize food based on municipal or state legislation, whose unsatisfactory reliability can be explained by differences in the interpretation and application of these laws between schools, an issue that emphasizes the inconsistency in the implementation of the rules.

In the inter-rater test, the differences can be attributed to the interpretation of the criteria by the evaluators, the lack of clarity or ambiguity in the instructions, or to variations in the observation and recording of the data, indicating the need for improvement of the training carried out. Other authors also encountered this scenario^30^ ^,^ ^54^ ^,^ ^55^, which demonstrates the complexity of measuring different dimensions of the food environment related to the characteristics of the items, which may involve subjectivity in the measurement or present variation in the availability of items.

In the intra-rater test, the results were even more satisfactory. Differences can be attributed to changes in the environment, such as availability and variety of foods, or to the behavior of participants between the two test applications. In the study conducted by Franco et al.^30^, whose objective was to evaluate content validity and reliability of an instrument for assessing the Brazilian university food environment, the differences found were due to variations in the environment, specifically in food offer. Borges et al.^29^, when assessing the reliability of a consumer food environment audit instrument, found small changes in the availability, price, quantity of brands, and advertising strategies of food. It is worth mentioning that the authors of the aforementioned study evaluated establishments such as supermarkets, hypermarkets, and markets, which show greater variation in price, brands, and advertising. This differs from the present study and from the study by Franco et al.^30^, who analyzed establishments such as school canteens and university cafeterias/restaurants, both inserted in the organizational environment, where the variety of the menu does not usually suffer as many changes and price readjustments are usually performed less often, in more specific moments such as the back-to-school period.

Still regarding the intra-rater test, authors of the study on the validity and reliability of the Perceived Nutrition Environment Measures Survey (NEMS-P) instrument^56^, translated and adapted to Brazil, also found acceptable ICC values. However, as observed in the present study, some dimensions showed lower performance - in the case of NEMS-P, issues related to prices, marketing in supermarkets, and food promotion, which showed low or even negative values. The authors attributed these results to the context of inflation in the country, the difficulties understanding the questions, and the limitations of psychometric methods to capture individual perceptions^56^.

Authors of a systematic review^57^, aimed at classifying and evaluating the quality of the methods for measuring the school food environment, identified that few studies carried out the stages of elaboration and evaluation of content validity and reliability of the instruments^57^. We emphasize the originality of the present study by taking all these steps for a specific instrument for measuring food commercialization in school canteens.

Among the strengths of the study, we mention content validity through qualitative and quantitative analysis and the evaluation of the reliability of the instrument in public and private schools, thus covering the two contexts of the school food environment. In addition, the instrument included a broad list of foods, based on the NOVA classification, and incorporated detailed information on the commercialization of food in canteens. The instrument includes questions for identifying the influence of the food industry on schools, enabling a more critical analysis of this context.

Among the limitations, we highlight the period of data collection, which lasted three months, not allowing the identification of seasonal differences. However, the study included two cities with distinct socioeconomic and demographic characteristics, which adds value to the analysis. For some of the items for which kappa and PABAK would be calculated, these statistics were not computed, which may be related to the reduced number of canteens evaluated, although the sample size has shown sufficient statistical power for the analyses. Furthermore, canteens of public schools were evaluated, where food commercialization is less frequent, as a result of the National School Feeding Program. In these schools, human resources limitations impact the structure of canteens, resulting in a high number of negative responses in the application of the instrument.

Within the context of the surveyed schools, the instrument proved to be reliable to measure the commercialization of food in school canteens, indicating applicability to similar contexts. Its use contributes to the advancement in assessing the food environment of these canteens, allowing detailed monitoring and providing subsidies for interventions that promote healthier environments and adequate diet for students.

## Supplementary Materials

Supplementary material 1

Supplementary material 2
