# Supplementary material for: Development, content validity, and reliability of an instrument to assess the commercialization of food and beverages in school canteens in Brazil
Source: Rev Bras Epidemiol. 2026 Apr 3;29:e260015. doi: 10.1590/1980-549720260015 (PMC13053033; doi:10.1590/1980-549720260015)
Supplement: Supplementary file 1 [file 1980-5497-rbepid-29-e260015-sppl2.pdf]

Tabela 1. Média do índice validade de conteúdo (IVC) e alterações realizadas no instrumento de avaliação da comercialização de alimentos e bebidas em cantinas escolares após consulta aos especialistas.

Seção	IVC				Alterações realizadas
	relevância	clareza das perguntas	clareza das respostas	Média do IVC	
Seção 1: Identificação e caracterização da cantina	0,94	0,96	0,93	0,94	-Padronização em alguns termos;<br>-Inclusão de ID para identificação da escola;<br>-Exclusão da razão social, CEP, nome fantasia e telefone;<br>-Alteração no item de "Número médio de clientes atendidos por dia (alunos, professores, colaboradores)" para "Qual o número médio de clientes atendidos por dia (alunos, professores, colaboradores)?";<br>-Inclusão de opção de resposta "Administrada pela associação de pais e alunos" no item "Quem é o responsável pela administração da cantina";<br>-Exclusão do item "Horário de funcionamento da cantinaenergét;<br>-Inclusão da opção de resposta "Café da manhã" no item "Quais os tipos de refeições que são ofertadas aos alunos?";<br>-Alteração das opções de resposta do item "Os cardápios das refeições abaixo estão disponíveis para os clientes?", especificando os cardápios em função das refeições;

	-Inclusão de duas alternativas de respostas no item “Como os preços são disponibilizados para os clientes?”, sendo elas: “na bandeja/no próprio produto" e “Aplicativo da escola/cantina”;<br>-Inclusão do termo “alimentares” e de exemplos do que são informações alimentares e nutricionais no item “As informações alimentares e nutricionais (ex: composição/ingredientes, calorias, carboidratos, proteínas, açúcar, gordura) das preparações culinárias que compõem o cardápio estão disponíveis para os clientes?”;<br>-Alteração na redação do item “Na cantina há oferta de açúcar na mesa/balcão, etc?” para “Na cantina há açúcar disponível na mesa/balcão?”;<br>-Inclusão da alternativa de resposta “Proposta de cardápio elaborada pela nutricionista” no item “Com base em que você seleciona os alimentos comercializados?”<br>-Inclusão da alternativa de resposta “Conta (fiado)” no item “Quais as formas de pagamento na cantina?”<br>-Alteração do termo “marcas” por “empresas” no item “Quais empresas patrocinam esses materiais na cantina?”<br>-Alteração do termo “restringe” por “proíbe” no item “A direção da escola proíbe a venda de algum tipo de alimento/produto pela cantina?”<br>-Inclusão do item “Há venda de alimentos e bebidas por outras pessoas da comunidade escolar (ex: alunos, professores, pais de alunos)?”

Seção 1.2: Alimentos comercializados na cantina	0,99	0,92	0,99	0,96	-Alteração da dimensão do tamanho e preço em todos os alimentos comercializados. De tamanho único, mínimo e máximo, bem como o preço único, mínimo e máximo, para o tamanho do menor preço e o menor preço dos alimentos e bebidas.<br><br>-Inclusão do conceito de refresco no Manual de Coleta de Dados, bem como exemplos<br><br>-Inclusão de exemplos no item “Café”<br><br>-Alteração no item, de modo a apresentar 'energético' e 'isotônico' separadamente<br><br>-Inclusão da medida “unidade” para aferição do tamanho de alguns alimentos, como preparações culinárias (salgados, sanduíches, tapioca) ou para aqueles em que não é possível ler a quantidade especificada no rótulo (balas, chicletes, pirulito, chocolate).<br><br>-Inclusão da avaliação sobre a existência da estratégia de venda (presença de combo ou promoção) e alteração do domínio publicidade para uma seção específica com a presença de diferentes tipos de publicidade.
Seção 1.3: Publicidade dos alimentos comercializados na cantina	1,00	0,88	0,99	0,95	- Seção criada após a consulta dos especialistas. Os itens desta seção faziam parte da seção 2.

Fonte: elaborada pelos autores.

Tabela suplementar S2. Estimativa de frequência de ocorrência e confiabilidade interobservador e intraobservador dos itens da Seção 1 (identificação e caracterização das cantinas) do instrumento para avaliação da comercialização de alimentos em cantinas (Brasil, 2024)

Variáveis	Interobservador							Intraobservador						
	Frequência de ocorrência/Média (DP)		Concordância (%)	Kappa/CCI (IC95%)	p (kappa)	PABAK	p (PABAK/IC95%)	Frequência de ocorrência/Média (DP)		Concordância (%)	Kappa/CCI (IC95%)	p (kappa)	PABAK	p (PABAK/IC95%)
	Obs.1	Ob.2						Obs.1	Obs.2					
Quantidade de clientes atendidos por dia *	315,7 (208,1)	315,7 (208,1)	100	1,0 (1,0-1,0)	-	1,0 (1,0-1,0)	-	315,7 (208,1)	316,07 (208,1)	85,7	1,0 (1,0-1,0)	0	-	-
**Administração da cantina (não conta)**														
A cantina é administrada por terceirizada	64,3	64,3	100	1,0 (1,0-1,0)	0,0001	1,0 (1,0-1,0)	-	64,3	64,3	100	1,0 (1,0-1,0)	0,0001	1,0 (1,0-1,0)	-
A cantina é administrada pela escola	35,7	35,7	100	1,0 (1,0-1,0)	0,0001	1,0 (1,0-1,0)	-	35,7	35,7	100	1,0 (1,0-1,0)	0,0001	1,0 (1,0-1,0)	-
Associação de pais e alunos	0	0	100	-	-	-	-	0	0	100	-	-	-	-
Professores	0	0	100	-	-	-	-	0	0	100	-	-	-	-
Outra	0	0	100	-	-	-	-	0	0	100	-	-	-	-
NS	0	0	100	-	-	-	-	0	0	100	-	-	-	-
**Número de funcionários da cantina (não conta)**														-
Menos de cinco funcionários	85,7	85,7	100	1,0 (1,0-1,0)	-	1,0 (1,0-1,0)	-	85,7	85,7	100	1,0 (1,0-1,0)	0	1,0 (1,0-1,0)	-
De cinco a dez funcionários	7,1	7,1	100	1,0 (1,0-1,0)	-	1,0 (1,0-1,0)	-	7,1	7,1	100	1,0 (1,0-1,0)	0	1,0 (1,0-1,0)	-
De dez a quinze funcionários	7,1	7,1	100	1,0 (1,0-1,0)	-	1,0 (1,0-1,0)	-	7,1	7,1	100	1,0 (1,0-1,0)	0	1,0 (1,0-1,0)	-
De 16 a 20	0	0	100	-	-	-		0	0	100	-	-	-	-
Mais de 20	0	0	100	-	-	-		0	0	100	-	-	-	-
**Presença Nutricionista**	28,5	28,5	100	1,0 (1,0-1,0)	0,0001	1,0 (1,0-1,0)	-	28,5	28,5	100	1,0 (1,0-1,0)	0,0001	1,0 (1,0-1,0)	-
Oferta de café da manhã	7,1	7,1	100	1,0 (1,0-1,0)	0,0001	1,0 (1,0-1,0)	-	7,1	7,1	100	1,0 (1,0-1,0)	0,0001	1,0 (1,0-1,0)	-
Oferta de almoço	21,4	21,4	100	1,0 (1,0-1,0)	0,0001	1,0 (1,0-1,0)	-	21,4	21,4	100	1,0 (1,0-1,0)	0,0001	1,0 (1,0-1,0)	-
Oferta de lanche	100	100	100	1,0 (1,0-1,0)	0,0001	1,0 (1,0-1,0)	-	100	100	100	1,0 (1,0-1,0)	0,0001	1,0 (1,0-1,0)	-
oferta do jantar	0	0	100	-	-	-	-	0	0	100	-	-	-	-
**Local de produção das preparações culinárias (não conta)**														
Na própria cantina	71,4	71,4	100	1,0 (1,0-1,0)	0,0001	1,0 (1,0-1,0)	-	71,4	71,4	100	1,0 (1,0-1,0)	0,0001	1,0 (1,0-1,0)	-
Na casa do funcionário ou do cantineiro	28,5	28,5	100	1,0 (1,0-1,0)	0,0001	1,0 (1,0-1,0)	-	28,5	28,5	100	1,0 (1,0-1,0)	0,0001	1,0 (1,0-1,0)	-
Na central de produção de alimentos para a cantina	14,2	14,2	100	1,0 (1,0-1,0)	0,0001	1,0 (1,0-1,0)	-	14,2	14,2	100	1,0 (1,0-1,0)	0,0001	1,0 (1,0-1,0)	-
Na cozinha da casa de vendedores autônomos ou microempresários	14,2	14,2	100	1,0 (1,0-1,0)	0,0001	1,0 (1,0-1,0)	-	14,2	14,2	100	1,0 (1,0-1,0)	0,0001	1,0 (1,0-1,0)	-
Na fábrica/indústria	100	100	100	1,0 (1,0-1,0)	0,0001	1,0 (1,0-1,0)	-	100	100	100	1,0 (1,0-1,0)	0,0001	1,0 (1,0-1,0)	-
Outros	0	0	100	-	-	-	-	0	0	100	-	-	-	-
**Cardápios das refeições disponíveis para os clientes (não conta)**														
Café da manhã	7,1	7,1	100	1,0 (1,0-1,0)	0,0001	1,0 (1,0-1,0)	-	7,1	7,1	100	1,0 (1,0-1,0)	0,0001	1,0 (1,0-1,0)	-
Almoço	21,4	21,4	100	1,0 (1,0-1,0)	0,0001	1,0 (1,0-1,0)	-	21,4	21,4	100	1,0 (1,0-1,0)	0,0001	1,0 (1,0-1,0)	-
Lanche	50	50	100	1,0 (1,0-1,0)	0,0001	1,0 (1,0-1,0)	-	50	50	100	1,0 (1,0-1,0)	0,0001	1,0 (1,0-1,0)	-
Jantar	0	0	100	-	-	-	-	0	0	100	-	-	-	-
**Formas de dispobilização dos cardápios (não conta)**														
Impresso	71,4	71,4	100	1,0 (1,0-1,0)	0,0041	1,0 (1,0-1,0)	-	71,4	71,4	100	1,0 (1,0-1,0)	0,0041	1,0 (1,0-1,0)	-
Display	0	0	100	-	-	-	-	0	0	100	-	0,0001	-	-
Banner/totem/tv	42,8	42,8	100	1,0 (1,0-1,0)	0,0041	1,0 (1,0-1,0)	-	42,8	42,8	100	1,0 (1,0-1,0)	0,0041	1,0 (1,0-1,0)	-
Internet/Qrcode	71,4	71,4	100	1,0 (1,0-1,0)	0,0041	1,0 (1,0-1,0)	-	71,4	71,4	100	1,0 (1,0-1,0)	0,0041	1,0 (1,0-1,0)	-
impresso disponível fora da cantina	14,2	14,2	100	1,0 (1,0-1,0)	0,0041	1,0 (1,0-1,0)	-	14,2	14,2	100	1,0 (1,0-1,0)	0,0041	1,0 (1,0-1,0)	-
Na bandeja/no próprio produto	0	0	100	-	-	-	-	0	0	100	-	-	-	-
Aplicativo da escola/cantina	28,5	28,5	100	1,0 (1,0-1,0)	0,0041	1,0 (1,0-1,0)	-	28,5	28,5	100	1,0 (1,0-1,0)	0,0041	1,0 (1,0-1,0)	-
Outros	0	0	100	-	-	-	-	0	0	100	-	-	-	-
**Preços disponibilizados para os clientes**	50	50	100	1,0 (1,0-1,0)	0,0001	1,0 (1,0-1,0)	-	50	50	100	1,0 (1,0-1,0)	0,0001	1,0 (1,0-1,0)	-
**Forma de disponibilizar os preços (não conta)**														-
No cardápio impresso	71,4	71,4	100	1,0 (1,0-1,0)	0,0041	1,0 (1,0-1,0)	-	71,4	71,4	100	1,0 (1,0-1,0)	0,0041	1,0 (1,0-1,0)	-
Display de mesa	0	0	100	-	-	-	-	0	0	100	-	-	-	-
Banner/totem/TV	42,8	42,8	100	1,0 (1,0-1,0)	0,0041	1,0 (1,0-1,0)	-	42,8	42,8	100	1,0 (1,0-1,0)	0,0041	1,0 (1,0-1,0)	-

Internet/QR code	71,4	71,4	100	1,0 (1,0-1,0)	0,0041	1,0 (1,0-1,0)	-	71,4	71,4	100	1,0 (1,0-1,0)	0,0041	1,0 (1,0-1,0)	-
No cardápio impresso disponível fora da cantina	14,2	14,2	100	1,0 (1,0-1,0)	0,0041	1,0 (1,0-1,0)	-	14,2	14,2	100	1,0 (1,0-1,0)	0,0041	1,0 (1,0-1,0)	-
Na bandeja/no próprio produto	0	0	100	-	-	-	-	0	7,1	92,8	0,8 (0,0-1,0)	0.0001	0,8 (0,6-1,0)	-
Aplicativo da escola/cantina	28,5	28,5	100	1,0 (1,0-1,0)	0,0041	1,0 (1,0-1,0)	-	28,5	28,5	100	1,0 (1,0-1,0)	0,0041	1,0 (1,0-1,0)	-
Outro	0	0	100	-	-	-	-	0	0	100	-	-	-	-
**Informações alimentares e nutricionais das preparações culinárias presentes**	0	0	100	-	-	-	-	0	0	100	-	-	-	-
**Alimentos para fins especiais presentes**	42,8	50	92,8	0,8 (0,6-1,0)	0,0006	0,8 (0,5-1,0)	0	42,8	42,8	100	1,0 (1,0-1,0)	0,0001	1,0 (1,0-1,0)	-
Presença de estrutura como mesa/balcão para consumo de lanche ou refeição	100	100	100	1,0	-	1,0 (1,0-1,0)	-	100	100	100	1,0	-	1,0 (1,0-1,0)	-
Presença de açúcar	0	0	100	-	-	-	-	0	0	100	-	-	-	-
Presença de sal	0	0	100	-	-	-	-	0	0	100	-	-	-	-
Presença de molhos UPP	0	0	100	-	-	-	-	0	0	100	-	-	-	-
**Critérios que os alimentos são comercializados (não conta)**														
Preferência dos alunos	92,8	92,8	100	1,0 (1,0-1,0)	0,0001	1,0 (1,0-1,0)	-	92,8	92,8	100	1,0 (1,0-1,0)	0,0001	1,0 (1,0-1,0)	-
Possibilidade de produção ou aquisição dos alimentos ou produtos	85,7	85,7	100	1,0 (1,0-1,0)	0,0001	1,0 (1,0-1,0)	-	85,7	85,7	100	1,0 (1,0-1,0)	0,0001	1,0 (1,0-1,0)	-
Mais vendidos	100	100	100	1,0	-	1,0 (1,0-1,0)	-	100	100	100	1,0	-	1,0 (1,0-1,0)	-
Maior percentual de lucro	64,2	64,2	100	1,0 (1,0-1,0)	0,0001	1,0 (1,0-1,0)	-	64,2	64,2	100	1,0 (1,0-1,0)	0,0001	1,0 (1,0-1,0)	-
Recomendação/determinação definida pela escola	92,8	92,8	100	1,0 (1,0-1,0)	0,0001	1,0 (1,0-1,0)	-	92,8	92,8	100	1,0 (1,0-1,0)	0,0001	1,0 (1,0-1,0)	-
Proposta de cardápio elaborada pela nutricionista	28,5	28,5	100	1,0 (1,0-1,0)	0,0001	1,0 (1,0-1,0)	-	28,5	28,5	100	1,0 (1,0-1,0)	0,0001	1,0 (1,0-1,0)	-
Permissão de acordo com legislação (lei, decreto, portaria) municipal ou estadual	100	100	100	1,0	-	1,0 (1,0-1,0)	-	100	92,8	92,8	0,0 (0,0-1,0)	-	0,8 (0,5-1,0)	0
Outro fator	0	0	100	-	-	-	-	0	0	100	-	-	-	-
**Formas de pagamento da cantina (não conta)**														
Dinheiro	85,7	85,7	100	1,0 (1,0-1,0)	0,0001	1,0 (1,0-1,0)	-	85,7	85,7	100	1,0 (1,0-1,0)	0,0001	1,0 (1,0-1,0)	-
Cartão de débito	28,5	28,5	100	1,0 (1,0-1,0)	0,0001	1,0 (1,0-1,0)	-	28,5	28,5	100	1,0 (1,0-1,0)	0,0001	1,0 (1,0-1,0)	-
Cartão de crédito	28,5	28,5	100	1,0 (1,0-1,0)	0,0001	1,0 (1,0-1,0)	-	28,5	28,5	100	1,0 (1,0-1,0)	0,0001	1,0 (1,0-1,0)	-
Cartão da cantina	21,4	28,5	92,8	0,8 (0,4-1,0)	0,0010	0,8 (0,5- 1,0)	0	21,4	21,4	100	1,0 (1,0-1,0)	0,0001	1,0 (1,0-1,0)	-
Pix	85,7	78,5	92,8	0,7 (0,3-1,0)	0,0017	0,8 (0,5-1,0)	0	85,7	85,7	100	1,0 (1,0-1,0)	0,0001	1,0 (1,0-1,0)	-
Vale alimentação/refeição	7,1	7,1	100	1,0 (1,0-1,0)	0,0001	1,0 (1,0-1,0)	-	7,1	7,1	100	1,0 (1,0-1,0)	0,0001	1,0 (1,0-1,0)	-
Aplicativo da cantina	7,1	7,1	100	1,0 (1,0-1,0)	0,0001	1,0 (1,0-1,0)	-	7,1	7,1	100	1,0 (1,0-1,0)	0,0001	1,0 (1,0-1,0)	-
Conta (fiado)	64,2	57,1	92,8	0,8 (0,5-1,0)	0,0006	0,8 (0,5-1,0)	0	64,2	64,2	100	1,0 (1,0-1,0)	0,0001	-	
Outros	0	0	100	-	-	-	-	0	0	100	-	-	-	-
**Recebeu/recebe algum material de incentivo/patrocínio/apoio de fornecedores**	21,4	21,4	100	1,0	0,0001	1,0 (1,0-1,0)	-	21,4	21,4	100	1,0 (1,0-1,0)	0,0001	1,0 (1,0-1,0)	-
Mobiliário (n=3)	0	0	100	-	-	-	-	0	0	100	-	-	-	-
Utrensílios (n=3)	0	0	100	-	-	-	-	0	0	100	-	-	-	-
Equipamentos (n=3)	100	100	100	1,0	-	1,0 (1,0-1,0)	-	0	0	100	-	-	-	-
Brindes (n=3)	0	0	100	-	-	-	-	0	0	100	-	-	-	-
Material de divulgação de produtos (n=3)	33,3	33,3	100	1,0 (1,0-1,0)	0,0416	1,0 (1,0-1,0)	-	33,3	33,3	100	1,0 (1,0-1,0)	0,0416	1,0 (1,0-1,0)	-
Uniforme (n=3)	0	0	100	-	-	-	-	0	0	100	-	-	-	-
Letreiro (n=3)	0	0	100	-	-	-	-	0	0	100	-	-	-	-
Material de divulgação de novos produtos (n=3)	33,3	33,3	100	1,0 (1,0-1,0)	0,0416	1,0 (1,0-1,0)	-	33,3	33,2	100	1,0 (1,0-1,0)	0,0416	1,0 (1,0-1,0)	-
Material de marketing para auxiliar nas vendas de promoções combinadas (n=3)	0	0	100	-	-	-	-	0	0	100	-	-	-	-
Comissão e incentivo financeiro (n=3)	0	0	100	-	-	-	-	0	0	100	-	-	-	-
Outros (n=3)	0	0	100	-	-	-	-	0	0	100	-	-	-	-
**Presença de ações que incentivem a alimentação saudável desenvolvidas pela cantina**	42,8	42,8	100	1,0 (1,0-1,0)	0,0001	1,0 (1,0-1,0)	-	42,8	42,8	100	1,0 (1,0-1,0)	0,0001	1,0 (1,0-1,0)	-
**Se sim, quais (questão aberta)**	-	-	-	-	-	-	-	-	-	-	-	-	-	-
**Material educativo sobre alimentação saudável na cantina**	21,4	21,4	100	1,0 (1,0-1,0)	0,0001	1,0 (1,0-1,0)	-	21,4	21,4	100	1,0 (1,0-1,0)	0,0001	1,0 (1,0-1,0)	-
Produzido pela própria cantina (n=3)	100	100	100	1,0	1,0000	1,0 (1,0-1,0)	-	100	100	100	1,0	-	1,0 (1,0-1,0)	-

Produzido pela nutricionista da cantina (n=3)	66,6	66,6	100	1,0 (1,0-1,0)	0,0416	1,0 (1,0-1,0)	-	66,6	66,6	100	1,0 (1,0-1,0)	0,0416	1,0 (1,0-1,0)	-
Produzido pela direção da escola (n=3)	33,3	33,3	100	1,0 (1,0-1,0)	0,0416	1,0 (1,0-1,0)	-	33,3	33,3	100	1,0 (1,0-1,0)	0,0416	1,0 (1,0-1,0)	-
Produzido pelos alunos da escola em atividade pedagógica (n=3)	0	0	100	-	-	-	-	0	0	100	-	-	-	-
Produzido por empresas de alimentos (n=3)	0	0	100	-	-	-	-	0	0	100	-	-	-	-
Produzido por orgãos do poder público (n=3)	0	0	100	-	-	-	-	0	0	100	-	-	-	-
Outros (n=3)	0	0	100	-	-	-	-	0	0	100	-	-	-	-
**Proibição da venda de algum tipo de alimento/produto**	92,8	92,8	100	1,0 (1,0-1,0)	-	1,0 (1,0-1,0)	-	92,8	92,8	100	1,0 (1,0-1,0)		1,0 (1,0-1,0)	-
**Oferta de refeição na cantina**	21,4	21,4	100	1,0 (1,0-1,0)	0,0001	1,0 (1,0-1,0)	-	21,4	21,4	100	1,0 (1,0-1,0)	0,0001	1,0 (1,0-1,0)	-
Menor preço da refeição prato feito *	15,0 (1,7)	15,0 (1,7)	100	1,0 (1,0-1,0)	-	1,0 (1,0-1,0)	-	15,0 (1,7)	15,0 (1,7)	100	1,0 (1,0-1,0)	-	1,0 (1,0-1,0)	-
Menor preço do quilo *	13,3 (13,3)	13,3 (13,3)	100	1,0 (1,0-1,0)	-	1,0 (1,0-1,0)	-	13,3 (13,3)	13,3 (13,3)	100	1,0 (1,0-1,0)	-	1,0 (1,0-1,0)	-
**Opção de combo na cantina**	14,2	14,2	100	1,0 (1,0-1,0)	0,0001	1,0 (1,0-1,0)	-	14,2	14,2	100	1,0 (1,0-1,0)	0,0001	1,0 (1,0-1,0)	-
**Opção de promoção na cantina**	7,1	7,1	100	1,0 (1,0-1,0)	0,0001	1,0 (1,0-1,0)	-	7,1	7,1	100	1,0 (1,0-1,0)	0,0001	1,0 (1,0-1,0)	-
**Venda de alimentos e bebidas por outras pessoas da comunidade escolar**	7,1	7,1	100	1,0 (1,0-1,0)	0,0001	1,0 (1,0-1,0)	-	7,1	7,1	100	1,0 (1,0-1,0)	0,0001	1,0 (1,0-1,0)	-

Legenda: * para as variáveis quantitativas foram calculadas a média (dp) e o Coeficiente de Correlação Intraclasse (CCI)

Tabela suplementar S3. Estimativa de frequência de ocorrência e confiabilidade interobservador e intraobservador dos itens da Seção 2 (Alimentos comercializados nas cantinas) do instrumento para avaliação da comercialização de alimentos em cantinas (Brasil, 2024)

Variáveis	Interobservador							Intraobservador						
	Frequência de ocorrência/Média (DP)		Concordância (%)	Kappa/CCI (IC95%)	p (kappa)	PABAK	p (PABAK)	Frequência de ocorrência/Média (DP)		Concordância (%)	Kappa/CCI (IC95%)	p (kappa/ICC)	PABAK	p (PABAK)
	Obs.1	Obs.2						Obs.1	Obs.2					
Refrigerante comum (presença)	21,4	21,4	100	1,0 (1,0-1,0)	0,0001	1,0 (1,0-1,0)	-	21,4	21,4	100	1,0 (1,0-1,0)	0,0001	1,0 (1,0-1,0)	-
Refrigerante comum (variedade)*	1,3 (0,5)	1,3 (0,5)	100	1,0 (1,0-1,0)	0,0000	-	-	1,3 (0,5)	1,3 (0,5)	100	1,0 (1,0-1,0)	0,0000	-	-
Refrigerante comum (tamanho)*	224,3 (42,1)	224,3 (42,1)	100	1,0 (1,0-1,0)	0,0000	-	-	224,3 (42,1)	224,3 (42,1)	100	1,0 (1,0-1,0)	0,0000	-	-
Refrigerante comum (preço)*	2,3 (1,1)	2,0 (1,0)	66,6	0,9 (-2,0-0,9)	0,0710	-	-	2,3 (1,1)	2,3 (1,1)	100	1,0 (1,0-1,0)	0,0000	-	-
Refrigerante comum (combo)	0	0	100	-	-	-	-	0	0	100	-	-	-	-
Refrigerante comum (promoção)	0	0	100	-	-	-	-	0	0	100	-	-	-	-
Refrigerante zero (presença)	0	0	100	-	-	-	-	0	0	100	-	-	-	-
Bebida a base de soja (presença)	7,1	7,1	100	1,0 (1,0-1,0)	0,0001	1,0 (1,0-1,0)	-	7,1	7,1	100	1,0 (1,0-1,0)	0,0001	1,0 (1,0-1,0)	-
Bebida a base de soja (variedade)*	1,0 (0,0)	1 (0,0)	100	-	-	-	-	1,0 (0,0)	1,0 (0,0)	100	-	-	-	-
Bebida a base de soja (tamanho) *	200,0 (0,0)	200 (0,0)	100	-	-	-	-	200,0 (0,0)	200,0 (0,0)	100	-	-	-	-
Bebida a base de soja (preço)*	3,5 (0,0)	3,5 (0,0)	0	-	-	-	-	3,5 (0,0)	3,5 (0,0)	100	-	-	-	-
Bebida a base de soja (combo)	0	0	100	-	-	-	-	0	0	100	-	-	-	-
Bebida a base de soja (promoção)	0	0	100	-	-	-	-	0	0	100	-	-	-	-
Bebida láctea e iogurte com sabor (comercializado)	14,2	14,2	100	1,0 (1,0-1,0)	0,0001	1,0 (1,0-1,0)	-	14,2	14,2	100	1,0 (1,0-1,0)	0,0001	1,0 (1,0-1,0)	-
Bebida láctea e iogurte com sabor (variedade)*	7,5 (4,9)	8 (4,2)	92,8	0,9 (-2,8-1,0)	0,0490	-	-	7,5 (4,9)	7,5 (4,9)	100	1,0 (1,0-1,0)	-	-	-
Bebida láctea e iogurte com sabor (tamanho)*	150,0 (50,0)	150,0 (50,0)	100	1,0 (1,0-1,0)	0,0000	-	-	150,0 (50,0)	150,0 (50,0)	100	1,0 (1,0-1,0)	0,0000	-	-
Bebida láctea e iogurte com sabor (preço)*	2,7 (0,3)	2,7 (0,3)	100	1,0 (1,0-1,0)	0,0000	-	-	2,7 (0,3)	2,7 (0,3)	100	1,0 (1,0-1,0)	0,0000	-	-
Bebida láctea e iogurte com sabor (combo)	0	0	100	-	-	-	-	0	0	100	-	-	-	-
Bebida láctea e iogurte com sabor (promoção)	0	0	100	-	-	-	-	0	0	100	-	-	-	-
Energético (comercializado)	0	0	100	-	-	-	-	0	0	100	-	-	-	-
Isotônico (comercializado)	7,1	7,1	100	1,0 (1,0-1,0)	0,0001	1,0 (1,0-1,0)	-	7,1	7,1	100	1,0 (1,0-1,0)	0,0001	1,0 (1,0-1,0)	-
Isotônico (variedade)*	5,0 (0,0)	5,0 (0,0)	100	-	-	-	-	5,0 (0,0)	5,0 (0,0)	100	-	-	-	-
Isotônico (tamanho)*	500,0 (0,0)	500,0 (0,0)	100	-	-	-	-	500,0 (0,0)	500,0 (0,0)	100	-	-	-	-
Isotônico (preço)*	7,0 (0,0)	7,0 (0,0)	100	-	-	-	-	7,0 (0,0)	7,0 (0,0)	100	-	-	-	-
Isotônico (combo)	0	0	100	-	-	-	-	0	0	100	-	-	-	-
Isotônico (promoção)	0	0	100	-	-	-	-	0	0	100	-	-	-	-
Néctar de fruta em caixinha, lata ou garrafa (comercializado)	42,8	42,8	100	1,0 (1,0-1,0)	0,0001	1,0 (1,0-1,0)	-	42,8	42,8	100	1,0 (1,0-1,0)	0,0001	1,0 (1,0-1,0)	-
Néctar de fruta em caixinha, lata ou garrafa (variedade)*	3,5 (1,2)	3,5 (1,2)	100	1,0 (1,0-1,0)	0,0000	-	-	3,5 (1,2)	3,5 (1,22)	100	1,0 (1,0-1,0)	0,0000	-	-
Néctar de fruta em caixinha, lata ou garrafa (tamanho)*	205,8 (14,2)	205,8 (14,2)	100	1,0 (1,0-1,0)	0,0000	-	-	205,8 (14,2)	205,8 (14,2)	100	1,0 (1,0-1,0)	0,0000	-	-
Néctar de fruta em caixinha, lata ou garrafa (preço)*	3,3 (0,9)	3,3 (0,9)	100	1,0 (1,0-1,0)	0,0000	-	-	3,3 (0,9)	3,3 (0,9)	100	1,0 (1,0-1,0)	0,0000	-	-
Néctar de fruta em caixinha, lata ou garrafa (promoção)	0	0	100	-	-	-	-	0	0	100	-	-	-	-
Néctar de fruta em caixinha, lata ou garrafa (combo)	0	0	100	-	-	-		0	0	100	-	-	-	-
Refresco (comercializado)	28,5	28,5	100	1,0 (1,0-1,0)	0,0001	1,0 (1,0-1,0)	-	28,5	28,5	100	1,0 (1,0-1,0)	0,0001	1,0 (1,0-1,0)	-
Refresco (variedade)*	1,2 (0,5)	1,2 (0,5)	100	1,0 (1,0-1,0)	0,0000	-		1,2 (0,5)	1,2 (0,5)	100	1,0 (1,0-1,0)	0,0000	-	-
Refresco (tamanho)*	200,0 (0,0)	200,0 (0,0)	100	-	-	-		200,0 (0,0)	200,0 (0,0)	100	-	-	-	-
Refresco (preço)*	1,0 (0,4)	0,87 (0,25)	75	0,8 (-1,4-0,9)	-	-		1,0 (0,4)	1,0 (0,4)	100	1,0 (1,0-1,0)	0,0000	-	-
Refresco (promoção)	0	0	100	-	-	-	-	0	0	100	-	-	-	-
Refresco (combo)	0	0	100	-	-	-	-	0	0	100	-	-	-	-
Chá pronto para beber (comercializado)	7,1	7,1	100	1,0 (1,0-1,0)	0,0001	1,0 (1,0-1,0)	-	7,1	7,1	100	1,0 (1,0-1,0)	0,0001	1,0 (1,0-1,0)	-
Chá pronto para beber (variedade)*	2,0 (0,0)	2,0 (0,0)	100	-	-	-	-	2,0 (0,0)	2,0 (0,0)	100	-	-	-	-
Chá pronto para beber (tamanho)*	500,0 (0,0)	500,0 (0,0)	100	-	-	-	-	500,0 (0,0)	500,0 (0,0)	100	-	-	-	-
Chá pronto para beber (preço)*	6,0 (0,0)	6,0 (0,0)	100	-	-	-	-	6,0 (0,0)	6,0 (0,0)	100	-	-	-	-

Chá pronto para beber (combo)	0	0	100	-	-	-	-	0	0	100	-		-	-
Chá pronto para beber (promoção)	0	0	100	-	-	-	-	0	0	100	-		-	-
Açaí com xarope (comercializado)	42,8	42,8	100	1,0 (1,0-1,0)	0,0001	1,0 (1,0-1,0)	-	42,8	42,8	100	1,0 (1,0-1,0)	0,0001	1,0 (1,0-1,0)	-
Açaí com xarope (variedade)*	1,0 (0,0)	1,0 (0,0)	100	-	-	-	-	1,0 (0,0)	1,0 (0,0)	100	-	-		-
Açaí com xarope (tamanho)*	200,1 (109,1)	200,1 (109,1)	100	1,0 (1,0-1,0)	0,0000	-	-	200,1 (109,1)	200,1 (109,1)	100	1,0 (1,0-1,0)	0,0000		-
Açaí com xarope (preço)*	8,6 (3,6)	8,6 (3,6)	100	1,0 (1,0-1,0)	0,0000	-	-	8,6 (3,6)	8,6 (3,6)	100	1,0 (1,0-1,0)	0,0000		-
Açaí com xarope (combo)	0	0	100	-	-	-	-	0	0	100	-	-	-	-
Açaí com xarope (promoção)	0	0	100	-	-	-	-	0	0	100	-	-	-	-
Açaí com toppings (comercializado)	35,7	35,7	100	1,0 (1,0-1,0)	0,0001	1,0 (1,0-1,0)	-	35,7	35,7	100	1,0 (1,0-1,0)	0,0001	1,0 (1,0-1,0)	-
Açaí com toppings (variedade)*	1,0 (0,0)	1,0 (0,0)	100	-	-	-	-	1,0 (0,0)	1,0 (0,0)	100	-	-		
Açaí com toppings (tamanho)*	240,0 (54,7)	240,0 (54,7)	100	1,0 (1,0-1,0)	-	-	-	240,0 (54,7)	240,0 (54,7)	100	1,0 (1,0-1,0)	-		
Açaí com toppings (preço)*	9,2 (3,8)	9,2 (3,8)	100	1,0 (1,0-1,0)	0,0000	-	-	9,2 (3,8)	9,2 (3,8)	100	1,0 (1,0-1,0)	0,0000		-
Açaí com toppings (combo)	0	0	100	-	-	-	-	0	0	100	-	-	-	-
Açaí com toppings (promoção)	0	0	100	-	-	-	-	0	0	100	-	-	-	-
Picolé ou sorvete(comercializado)	64,2	64,2	100	1,0 (1,0-1,0)	0,0001	1,0 (1,0-1,0)	-	64,2	64,2	100	1,0 (1,0-1,0)	0,0001	1,0 (1,0-1,0)	-
Picolé ou sorvete (variedade)*	5,3 (2,5)	5,3 (2,5)	100	1,0 (1,0-1,0)	0,0000	-	-	5,3 (2,5)	5,3 (2,5)	100	1,0 (1,0-1,0)	0,0000	-	-
Picolé ou sorvete (tamanho)*	35,4 (66,2)	35,4 (66,2)	100	1,0 (1,0-1,0)	0,0000	-	-	35,4 (66,2)	35,4 (66,2)	100	1,0 (1,0-1,0)	0,0000	-	-
Picolé ou sorvete (preço)*	2,75 (1,6)	2,75 (1,6)	100	1,0 (1,0-1,0)	0,0000	-	-	2,7 (1,6)	2,7 (1,6)	100	1,0 (1,0-1,0)	0,0000	-	-
Picolé ou sorvete (combo)	0	0	100	-	-	-	-	0	0	100	-	-	-	-
Picolé ou sorvete (promoção)	7,1	7,1	100	1,0 (1,0-1,0)	0,0000	1,0 (1,0-1,0)	-	7,1	7,1	100	1,0 (1,0-1,0)	0,0000	1,0 (1,0-1,0)	-
Chocolate e bombom (comercializado)	21,4	21,4	100	1,0 (1,0-1,0)	0,0001	1,0 (1,0-1,0)	-	21,4	21,4	100	1,0 (1,0-1,0)	0,0001	1,0 (1,0-1,0)	-
Chocolate e bombom (variedade)*	2,0 (1,0)	2,0 (1,0)	100	1,0 (1,0-1,0)	0,0000	-	-	2,0 (1,0)	2,0 (1,0)	100	1,0 (1,0-1,0)	0,0000		
Chocolate e bombom (tamanho)*	1,0 (0,0)	1,0 (0,0)	100	-	-	-	-	1,0 (0,0)	1,0 (0,0)	100	-	-		
Chocolate e bombom (preço)*	2,1 (1,7)	2,1 (1,7)	100	1,0 (1,0-1,0)	0,0000	-	-	2,1 (1,7)	2,1 (1,7)	100	1,0 (1,0-1,0)	0,0000		
Chocolate e bombom (combo)	0	0	100	-	-	-	-	0	0	100	-	-	-	-
Chocolate e bombom (promoção)	0	0	100	-	-	-	-	0	0	100	-	-	-	-
Guloseimas (comercializado)	14,2	14,2	100	1,0 (1,0-1,0)	0,0001	1,0 (1,0-1,0)	-	14,2	14,2	100	1,0 (1,0-1,0)	0,0001	1,0 (1,0-1,0)	-
Guloseimas (variedade)*	6,0 (2,8)	6,0 (2,8)	100	1,0 (1,0-1,0)	0,0000	-	-	6,0 (2,8)	6,0 (2,8)	100	1,0 (1,0-1,0)	0,0000		
Guloseimas (tamanho)*	1,0 (0,0)	1,0 (0,0)	100	-	-	-	-	1,0 (0,0)	1,0 (0,0)	100	-	-		
Guloseimas (preço)*	0,1 (0,0)	0,1 (0,0)	100	1,0 (1,0-1,0)	0,0000	-	-	0,1 (0,0)	0,1 (0,0)	100	1,0 (1,0-1,0)	0,0000		
Guloseimas (combo)	0	0	100	-	-	-	-	0	0	100	-	-	-	-
Guloseimas (promoção)	0	0	100	-	-	-	-	0	0	100	-	-	-	-
Barra de cerais (comercializado)	28,5	28,5	100	1,0 (1,0-1,0)	0,0001	1,0 (1,0-1,0)	-	28,5	28,5	100	1,0 (1,0-1,0)	-	1,0 (1,0-1,0)	-
Barra de cerais (variedade)*	2,7 (0,9)	2,7 (0,9)	100	1,0 (1,0-1,0)	-	-		2,7 (0,9)	3,2 (0,9)	92,8	0,6 (-4,7-0,9)	0,2200		
Barra de cerais (tamanho)*	38,7 (30,9)	38,7 (30,9)	100	1,0 (1,0-1,0)	0,0000	-		38,7 (30,9)	38,7 (30,9)	100	1,0 (1,0-1,0)	0,0000		
Barra de cerais (preço)*	3,1 (0,6)	3,1 (0,6)	100	1,0 (1,0-1,0)	-	-		3,1 (0,6)	3,0 (0,8)	75,0	0,9 (0,5 - 0,9)	0,0080		
Barra de cerais (combo)	0	0	100	-	-	-	-	0	0	100	-		-	-
Barra de cerais (promoção)	0	0	100	-	-	-	-	0	0	100	-		-	-
Cereal Matinal (comercializado)	0	0	100	-	-	-		0	0	100	-	-	-	-
Bolo UP (comercializado)	14,2	14,2	100	1,0 (1,0-1,0)	0,0001	1,0 (1,0-1,0)	-	14,2	14,2	100	1,0 (1,0-1,0)	0,0001	1,0 (1,0-1,0)	-
Bolo UP (variedade)*	3,0 (0,0)	3,0 (0,0)	100	-	-	-		3,0 (0,0)	3,0 (0,0)	100	-	-	-	
Bolo UP (tamanho)*	1,0 (0,0)	1,0 (0,0)	100	-	-	-		1,0 (0,0)	1,0 (0,0)	100	-	-		
Bolo UP (preço)*	2,2 (0,3)	2,2 (0,3)	100	1,0 (1,0-1,0)	0,0000		-	2,2 (0,3)	2,2 (0,3)	100	1,0 (1,0-1,0)	0,0000		
Bolo UP (combo)	14,2	14,2	100	1,0 (1,0-1,0)	0,0001	1,0 (1,0-1,0)	-	14,2	14,2	100	1,0 (1,0-1,0)	0,0001	1,0 (1,0-1,0)	-
Bolo UP (promoção)	0	0	100	-	-	-	-	0	0	100	-	-	-	-

Doce com ingredientes UP (comercializado)	28,5	28,5	100	1,0 (1,0-1,0)	0,0001	1,0 (1,0-1,0)	-	28,5	28,5	100	1,0 (1,0-1,0)	0,0001	1,0 (1,0-1,0)	-
Doce com ingredientes UP (variedade)*	1,2 (0,2)	1,2 (0,2)	100	1,0 (1,0-1,0)	0,0000	-	-	1,2 (0,2)	1,2 (0,2)	100	1,0 (1,0-1,0)	0,0000		
Doce com ingredientes UP (tamanho)*	25,5 (14,1)	25,5 (14,1)	100	1,0 (1,0-1,0)	0,0000	-	-	25,5 (14,1)	25,5 (14,1)	100	1,0 (1,0-1,0)	0,0000		
Doce com ingredientes UP (preço)*	3,4 (1,5)	3,4 (1,5)	100	1,0 (1,0-1,0)	0,0000	-	-	3,4 (1,5)	3,4 (1,5)	100	1,0 (1,0-1,0)	0,0000		
Doce com ingredientes UP (combo)	0	0	100	-	-	-	-	0	0	100	-	-	-	-
Doce com ingredientes UP (promoção)	0	0	100	-	-	-	-	0	0	100	-	-	-	-
Salada de frutas com toppings (comercializado)	0	0	-	-	-	-	-	0	0	100	-	-	-	-
Biscoito doce UP (comercializado)	28,5	28,5	100	1,0 (1,0-1,0)	0,0001	1,0 (1,0-1,0)	-	28,5	28,5	100	1,0 (1,0-1,0)	0,0001	1,0 (1,0-1,0)	-
Biscoito doce UP (variedade)*	3,0 (0,7)	3,0 (0,7)	100	1,0 (1,0-1,0)	0,0000	-	-	3,0 (0,7)	3,0 (0,7)	100	1,0 (1,0-1,0)	0,0000		
Biscoito doce UP (tamanho)*	38,2 (37,2)	38,2 (37,2)	100	1,0 (1,0-1,0)	0,0000	-	-	38,2 (37,2)	38,2 (37,2)	100	1,0 (1,0-1,0)	0,0000		
Biscoito doce UP (preço)*	2,5 (0,5)	2,5 (0,5)	100	1,0 (1,0-1,0)	0,0000	-	-	2,5 (0,5)	2,5 (0,5)	100	1,0 (1,0-1,0)	0,0000		
Biscoito doce UP (combo)	0	0	100	-	-	-	-	0	0	-	-	-	-	-
Biscoito doce UP (promoção)	0	0	100	-	-	-	-	0	0	-	-	-	-	-
Salgadinho de pacote (comercializado)	28,5	21,4	92,8	0,8 (0,4-1,0)	0,0010	0,8 (0,5-1,0)	0	28,5	28,5	100	1,0 (1,0-1,0)	0,0001	1,0 (1,0-1,0)	-
Salgadinho de pacote (variedade)*	2,0 (1,4)	2,0 (1,7)	100	1,0 (1,0-1,0)	0,0000	-	-	2,0 (1,4)	2,0 (1,4)	100	1,0 (1,0-1,0)	0,0000	-	-
Salgadinho de pacote (tamanho)*	1,0	0,7	92,8	-	-	-	-	1	1	100	1,0 (1,0-1,0)	0,0000	-	-
Salgadinho de pacote (preço)*	2,8 (0,5)	2,5 (0,8)	92,8	1,0 (1,0-1,0)	0,0000	-	-	2,8 (0,5)	2,8 (0,5)	100	1,0 (1,0-1,0)	-	-	-
Salgadinho de pacote (combo)	0	0	100	-	-	-	-	0	0	100	-	-	-	-
Salgadinho de pacote (promoção)	0	0	100	-	-	-	-	0	0	100	1,0 (1,0-1,0)	0,0001	1,0 (1,0-1,0)	-
Pão de queijo UP (comercializado)	35,7	35,7	100	1,0 (1,0-1,0)	0,0001	1,0 (1,0-1,0)	-	35,7	35,7	100	1,0 (1,0-1,0)	0,0001	1,0 (1,0-1,0)	-
Pão de queijo UP (variedade)*	1,1 (0,3)	1,1 (0,3)	100	1,0 (1,0-1,0)	0,0000			1,1 (0,3)	1,1 (0,3)	100	1,0 (1,0-1,0)	0,0000		
Pão de queijo UP (tamanho)*	1,0 (0,0)	1,0 (0,0)	100	-	-			1,0 (0,0)	1,0 (0,0)	100	-	-		
Pão de queijo UP (preço)*	2,9 (0,1)	2,9 (0,1)	100	1,0 (1,0-1,0)	-			2,9 (0,1)	2,9 (0,1)	100	1,0 (1,0-1,0)	-		
Pão de queijo UP (combo)	0	0	100	-	-	-	-	0	0	100	-	-	-	-
Pão de queijo UP (promoção)	0	0	100	-	-	-	-	0	0	100	-	-	-	-
Tapioca com recheio UP (Comercializado)	0	0	100	-	-	-	-	0	0	100	-	-	-	-
Pizza com recheio UP (comercializado)	78,5	78,5	100	1,0 (1,0-1,0)	0,0001	1,0 (1,0-1,0)	-	78,5	78,5	100	1,0 (1,0-1,0)	0,0001	1,0 (1,0-1,0)	-
Pizza com recheio UP (variedade)*	1,3 (0,6)	1,0 (0,3)	81,8	0,3 (-1,3-0,8)	0,2300			1,3 (0,6)	1,3 (0,6)	100	1,0 (1,0-1,0)	0,0000		
Pizza com recheio UP (tamanho)*	1,0 (0,0)	1,0 (0,0)	100	-	-			1,0 (0,0)	1,0 (0,0)	100	-	-		
Pizza com recheio UP (preço)*	4,4 (1,3)	4,4 (1,3)	100	1,0 (1,0-1,0)	0,0000			4,4 (1,3)	4,4 (1,3)	90,9	0,9 (0,9-0,9)	0,0000		
Pizza com recheio UP (combo)	0	0	100	-	-	-	-	0	0	100	-	-	-	-
Pizza com recheio UP (promoção)	0	0	100	-	-	-	-	0	0	100	-	-	-	-
Salgado assado com recheio UP (comercializado)	50	50	100	1,0 (1,0-1,0)	0,0001	1,0 (1,0-1,0)	-	50,0	50,0	100	1,0 (1,0-1,0)	0,0001	1,0 (1,0-1,0)	-
Salgado assado com recheio UP (variedade)*	1,5 (0,7)	1,5 (0,7)	100	1,0 (1,0-1,0)	0,0000	-	-	1,5 (0,7)	1,5 (0,7)	100	1,0 (1,0-1,0)	0,0000		
Salgado assado com recheio UP (tamanho)*	1,0 (0,0)	1,0 (0,0)	100	-	-	-	-	1,0 (0,0)	1,0 (0,0)	100	-	-		
Salgado assado com recheio UP (preço)*	4,3 (1,1)	4,3 (1,1)	100	1,0 (1,0-1,0)	0,0000	-	-	4,3 (1,1)	4,3 (1,1)	100	1,0 (1,0-1,0)	0,0000		
Salgado assado com recheio UP (combo)	0	0	100	-	-	-	-	0	0	100	-	-	-	
Salgado assado com recheio UP (promoção)	0	0	100	-	-	-	-	0	0	100	-	-	-	
Salgado frito com recheio UP (comercializado)	0	0	100	-	-	-	-	0	0	100	-	-	-	
Sanduíche com recheio UP (comercializado)	71,4	71,4	100	1,0 (1,0-1,0)	0,0001	1,0 (1,0-1,0)	-	71,4	71,4	100	1,0 (1,0-1,0)	0,0001	1,0 (1,0-1,0)	-
Sanduíche com recheio UP (variedade)*	1,2 (0,6)	1,0 (0,6)	90	0,0 (-3,0-0,7)	0,5000	-	-	1,2 (0,2)	1,2 (0,2)	100	1,0 (1,0-1,0)	0,0000		
Sanduíche com recheio UP (tamanho)*	1,0 (0,0)	1,0 (0,0)	100	-	-	-	-	1,0 (0,0)	1,0 (0,0)	100	-	-		
Sanduíche com recheio UP (preço)*	4,4 (1,7)	4,5 (1,9)	90	0,9 (0,9-0,9)	0,0000	-	-	4,4 (1,7)	4,4 (1,7)	100	1,0 (1,0-1,0)	-		
Sanduíche com recheio UP (combo)	0	0	100	-	-	-	-	0	0	100	-	-	-	
Sanduíche com recheio UP (promoção)	0	0	100	-	-	-	-	0	0	100	-	-	-	

Pipoca doce de pacote (promoção)	14,2	14,2	100	1,0 (1,0-1,0)	0,0001	-	-	14,2	7,1	92,8	0,6 (-0,0-1,0)	0,0050	0,8 (0,5-1,0)	0
Pipoca doce de pacote (variedade)*	1,0 (0,0)	1,0 (0,0)	100	-	-	-	-	1,0 (0,0)	1,0 (0,0)	100	-	-		
Pipoca doce de pacote (tamanho)*	1	1	100	-	-	-	-	1	1	100	-	-		
Pipoca doce de pacote (preço)*	1,5 (0,7)	1,5 (0,7)	100	1,0 (1,0-1,0)	-	-	-	1,5 (0,7)	1,0 (0,0)	100	-	-		
Pipoca doce de pacote (combo)	0	0	100	-	-	-	-	0	0	100	-	-	-	-
Pipoca doce de pacote (promoção)	0	0	100	-	-	-	-	0	0	100	-	-	-	-
Pipoca UP (comercializada)	0	0	100	-	-	-	-	0	0	100	-	-	-	-
Pipoca com grão natural (comercializada)	64,2	64,2	100	1,0 (1,0-1,0)	0,0001	1,0 (1,0-1,0)	-	64,2	64,2	100	1,0 (1,0-1,0)	0,0001	1,0 (1,0-1,0)	-
Pipoca com grão natural (variedade)*	1,0 (0,0)	1,0 (0,0)	100	-	-	-	-	1,0 (0,0)	1,0 (0,0)	100	-	-		
Pipoca com grão natural (tamanho)*	28,6 (7,5)	28,6 (7,5)	100	1,0 (1,0-1,0)	0,0000	-	-	28,6 (7,5)	28,6 (7,5)	100	1,0 (1,0-1,0)	0,0000		
Pipoca com grão natural (preço)*	1,6 (1,1)	1,6 (1,1)	100	1,0 (1,0-1,0)	0,0000	-	-	1,6 (1,1)	1,9 (1,2)	88,9	0,9 (0,6-0,9)	0,0010		
Pipoca com grão natural (combo)	0	7,1	92,8	0,8 (0,0-1,0)	0,0002	0,8 (0,6-1,0)	0	0	0	100	1,0 (1,0-1,0)	0,0001	1,0 (1,0-1,0)	-
Pipoca com grão natural (promoção)	0	0	100	-	-	-	-	0	0	100	-	-	-	
Chá natural (comercializado)	0	0	100	-	-	-	-	0	0	100	-	-	-	
Leite batido com fruta (comercializado)	7,1	7,1	100	1,0 (1,0-1,0)	0,0001	1,0 (1,0-1,0)	-	7,1	7,1	100	1,0 (1,0-1,0)	0,0001	1,0 (1,0-1,0)	-
Leite batido com fruta (variedade)*	2,0 (0,0)	2,0 (0,0)	100	-	-	-	-	2,0 (0,0)	2,0 (0,0)	100	-	-		
Leite batido com fruta (tamanho)*	300,0 (0,0)	300,0 (0,0)	100	-		-	-	300,0 (0,0)	300,0 (0,0)	100	-	0,0000		
Leite batido com fruta (preço)*	5,0 (0,0)	5,0 (0,0)	100	-	-	-	-	5,0 (0,0)	5,0 (0,0)	100	-	-		
Leite batido com fruta (combo)	0	0	100	-	-	-	-	0	0	-	-	-	-	-
Leite batido com fruta (promoção)	0	0	100	-	-	-	-	0	0	-	-	-	-	-
Água de coco (comercializado)	21,4	21,4	100	1,0 (1,0-1,0)	0,0001	1,0 (1,0-1,0)	-	21,4	21,4	100	1,0 (1,0-1,0)	0,0001	1,0 (1,0-1,0)	-
Água de coco (variedade)*	1,0 (0,0)	1,0 (0,0)	100	-	-	-		1,0 (0,0)	1,0 (0,0)	100	-	-		
Água de coco (tamanho)*	200,0 (0,0)	200,0 (0,0)	100	-	-	-		200,0 (0,0)	200,0 (0,0)	100	-	-		
Água de coco (preço)*	4,4 (1,1)	4,4 (1,1)	100	1,0 (1,0-1,0)	0,0000			4,4 (1,1)	4,4 (1,1)	100	1,0 (1,0-1,0)	0,0000		
Água de coco (combo)	0	0	100	-	-	-	-	0	0	100	-	-	-	-
Água de coco (promoção)	0	0	100	-	-	-	-	0	0	100	-	-	-	-
Água mineral (com ou sem gás) - cpmercializado	35,7	35,7	100	1,0 (1,0-1,0)	0,0001	1,0 (1,0-1,0)	-	35,7	35,7	100	1,0 (1,0-1,0)	0,0001	1,0 (1,0-1,0)	-
Água mineral (com ou sem gás) - variedade*	1,0 (0,0)	1,0 (0,0)	100	-	-	-	-	1,0 (0,0)	1,0 (0,0)	100	-	-		
Água mineral (com ou sem gás) - tamanho*	500,0 (0,0)	500,0 (0,0)	100	-	-	-	-	500,0 (0,0)	500,0 (0,0)	100	-	-		
Água mineral (com ou sem gás)- preço*	3,3 (0,8)	3,3 (0,8)	100	1,0 (1,0-1,0)	0,0000	-	-	3,3 (0,8)	3,3 (0,8)	100	1,0 (1,0-1,0)	0,0000		
Água mineral (com ou sem gás) - combo	0	0	100	-	-	-	-	0	0	100	-	-	-	-
Água mineral (com ou sem gás)- promoção	0	0	100	-	-	-	-	0	0	100	-	-	-	-
Suco natural da fruta (in natura ou polpa de fruta) - comercialiado	57,1	57,1	100	1,0 (1,0-1,0)	0,0001	1,0 (1,0-1,0)	-	57,1	57,1	100	1,0 (1,0-1,0)	0,0001	1,0 (1,0-1,0)	-
Suco natural da fruta (in natura ou polpa de fruta) - variedade*	2,5 (1,9)	2,5 (1,9)	100	1,0 (1,0-1,0)	-	-	-	2,5 (1,9)	2,6 (1,8)	87,5	0,9 (0,9-0,9)	0,0000		
Suco natural da fruta (in natura ou polpa de fruta) - tamanho *	200,0 (0,0)	200,0 (0,0)	100	-	-	-	-	200,0 (0,0)	200,0 (0,0)	100	-	-		
Suco natural da fruta (in natura ou polpa de fruta) - preço*	3,4 (1,0)	3,4 (1,0)	87,5	0,9 (0,9-1,0)	0,0000	-	-	3,4 (1,0)	3,4 (1,0)	100	1,0 (1,0-1,0)	-		
Suco natural da fruta (in natura ou polpa de fruta) - combo	7,1	7,1	100	1,0 (1,0-1,0)	0,0000	1,0 (1,0-1,0)	-	7,1	7,1	100	1,0 (1,0-1,0)	0,0000	1,0 (1,0-1,0)	-
Suco natural da fruta (in natura ou polpa de fruta) - promoção	0	0	100	-		-	-	0	0	100	-	-	-	-
Suco 100% integral em caixinha, lata ou garrafa (comercializado)	14,2	14,2	100	1,0 (1,0-1,0)	0,0001	-0,2 (-0,8-0,2)	0,3	14,2	14,2	100	1,0 (1,0-1,0)	0,0001	1,0 (1,0-1,0)	
Suco 100% integral em caixinha, lata ou garrafa (variedade)*	1,5 (0,7)	1,5 (0,7)	100	1,0 (1,0-1,0)	-	-	-	1,5 (0,7)	1,5 (0,7)	100	1,0 (1,0-1,0)	-		
Suco 100% integral em caixinha, lata ou garrafa (tamanho)*	300,0 (0,0)	300,0 (0,0)	100	-	-	-	-	300,0 (0,0)	300,0 (0,0)	100	-	-		
Suco 100% integral em caixinha, lata ou garrafa (preço)*	5,7 (1,0)	5,7 (1,0)	100	1,0 (1,0-1,0)	0,0000	-	-	5,7 (1,0)	5,7 (1,0)	100	1,0 (1,0-1,0)	0,0000		

Suco 100% integral em caixinha, lata ou garrafa (combo)	0	0	100	-	-	-	-	0	0	100	-	-	-	-
Suco 100% integral em caixinha, lata ou garrafa (promoção)	0	0	100	-	-	-	-	0	0	100	-	-	-	-
Café (coado ou expresso) (comercializado)	14,2	21,4	92,8	0,7 (0,3-1,0)	0,0017	0,8 (0,5- 1,0)	0	14,2	21,4	92,8	0,7 (0,3-1,0)	0,0017	0,8 (0,5- 1,0)	0
Café (coado ou expresso) (variedade)*	1,5 (0,7)	1,3 (0,5)	92,8	1,0 (1,0-1,0)	0,0000	-	-	1,5 (0,7)	1,3 (0,5)	92,8	1,0 (1,0-1,0)	0,0000		
Café (coado ou expresso) (tamanho)*	100,0 (0,0)	100,0 (0,0)	92,8	-	-	-	-	100,0 (0,0)	100,0 (0,0)	92,8	-	-		
Café (coado ou expresso) (preço)*	2,0 (0,7)	2,0 (0,9)	85,7	0,9 (-24,9-1,0)	0,1200	-	-	2,0 (0,7)	2,2 (0,6)	92,8	1,0 (1,0-1,0)	0,0000		
Café (coado ou expresso) (combo)	0	0	100	-	-	-	-	0	0	100	-	-	-	-
Café (coado ou expresso) (promoção)	0	0	100	-	-	-	-	0	0	100	-	-	-	-
Fruta fresca (comercializado)	28,5	28,5	100	1,0 (1,0-1,0)	0,0001	1,0 (1,0-1,0)	-	28,5	28,5	100	1,0 (1,0-1,0)	0,0001	1,0 (1,0-1,0)	-
Fruta fresca (variedade)*	3,0 (0,0)	3,0 (1,6)	100	0,0 (14,4-0,9)	0,5000	-	-	3,0 (0,0)	3,0 (0,0)	100	-	-		
Fruta fresca (tamanho)*	1,0 (0,0)	1,0 (0,0)	100	-	-	-	-	1,0 (0,0)	1,0 (0,0)	100	-	-		
Fruta fresca (preço)*	3,1 (1,2)	3,1 (1,2)	100	1,0 (1,0-1,0)	0,0000	-	-	3,1 (1,2)	3,1 (1,2)	100	1,0 (1,0-1,0)	0,0000		
Fruta fresca (combo)	7,1	7,1	100	1,0 (1,0-1,0)	0,0000	1,0 (1,0-1,0)	-	7,1	7,1	100	1,0 (1,0-1,0)	0,0000	1,0 (1,0-1,0)	-
Fruta fresca (promoção)	0	0	100	-	-	-	-	0	0	-	-	-	-	-
Fruta seca/desidratada (comercializado)	0	0	100	-	-	-	-	0	0	-	-	-	-	-
Salada de fruta simples (comercializado)	28,5	28,5	100	1,0 (1,0-1,0)	0,0001	1,0 (1,0-1,0)	-	28,5	28,5	100	1,0 (1,0-1,0)	0,0001	1,0 (1,0-1,0)	-
Salada de fruta simples (variedade)*	1,0 (0,0)	1,0 (0,0)	100	-	-	-	-	1,0 (0,0)	1,0 (0,0)	100	-	-		
Salada de fruta simples (tamanho)*	225,0 (64,5)	225,0 (64,5)	100	1,0 (1,0-1,0)	0,0000	-	-	225,0 (64,5)	225,0 (64,5)	100	1,0 (1,0-1,0)	0,0000		
Salada de fruta simples (preço)*	5,3 (0,9)	5,3 (0,9)	100	1,0 (1,0-1,0)	0,0000	-	-	5,3 (0,9)	5,3 (0,9)	100	1,0 (1,0-1,0)	0,0000		
Salada de fruta simples (combo)	0	0	100	-	-	-	-	0	0	100	-	-	-	-
Salada de fruta simples (promoção)	0	0	100	-	-	-	-	0	0	100	-	-	-	-
Doce de frutas (comercializado)	21,4	21,4	100	1,0 (1,0-1,0)	0,0001	1,0 (1,0-1,0)	-	21,4	21,4	100	1,0 (1,0-1,0)	0,0001	1,0 (1,0-1,0)	-
Doce de frutas (variedade)*	1,3 (0,5)	1,0 (0,0)	100	0,0 (-38,0-0,9)	0,5000	-	-	1,3 (0,5)	1,3 (0,5)	100	1,0 (1,0-1,0)	0,0000		
Doce de frutas (tamanho)*	1,0 (0,0)	1,0 (0,0)	100	-	-	-	-	1,0 (0,0)	1,0 (0,0)	100	-	-		
Doce de frutas (preço)*	0,8 (0,2)	0,8 (0,2)	100	1,0 (1,0-1,0)	0,0000	-	-	0,8 (0,2)	0,8 (0,2)	100	1,0 (1,0-1,0)	0,0000		
Doce de frutas (combo)	0	0	100	-	-	-	-	0	0	100	-	-	-	-
Doce de frutas (promoção)	0	0	100	-	-	-	-	0	0	100	-	-	-	-
Pão de queijo (preparação culinária)- comercializado	57,1	57,1	100	1,0 (1,0-1,0)	0,0001	1,0 (1,0-1,0)	-	57,1	57,1	100	1,0 (1,0-1,0)	0,0001	1,0 (1,0-1,0)	-
Pão de queijo (preparação culinária)- variedade*	1,1 (0,3)	1,1 (0,3)	100	1,0 (1,0-1,0)	0,0000	-	-	1,1 (0,3)	1,1 (0,3)	100	1,0 (1,0-1,0)	0,0000	-	-
Pão de queijo (preparação culinária) - tamanho *	1,0 (0,0)	1,0 (0,0)	100	-	-	-	-	1,0 (0,0)	1,0 (0,0)	100	-	-	-	-
Pão de queijo (preparação culinária) - preço *	3,2 (0,9)	3,2 (0,9)	100	1,0 (1,0-1,0)	0,0000	-	-	3,2 (0,9)	3,2 (0,9)	100	1,0 (1,0-1,0)	0,0000	-	-
Pão de queijo (preparação culinária) - combo	7,1	14,2	92,8	0,8 (0,7-1,0)	0,0000	0,8 (0,6-1,0)	0	7,1	7,1	100	1,0 (1,0-1,0)	0,0000	1,0 (1,0-1,0)	-
Pão de queijo (preparação culinária) - promoção	0	0	100	-	0,0001	-	-	0	0	100	-	-	-	-
Açaí sem xarope (comercializado)	14,2	14,2	100	1,0 (1,0-1,0)	0,0001	1,0 (1,0-1,0)	-	14,2	14,2	100	1,0 (1,0-1,0)	0,0001	1,0 (1,0-1,0)	-
Açaí sem xarope (variedade)*	1,0 (0,0)	1,0 (0,0)	100	-	-	-	-	1,0 (0,0)	1,0 (0,0)	100	-	-		-
Açaí sem xarope (tamanho)*	200,0 (0,0)	200,0 (0,0)	100	-	-	-	-	200,0 (0,0)	200,0 (0,0)	100	-	-		-
Açaí sem xarope (preço)*	9,2 (4,6)	9,2 (4,6)	100	1,0 (1,0-1,0)	0,0000	-	-	9,2 (4,6)	9,2 (4,6)	100	1,0 (1,0-1,0)	0,0000		-
Açaí sem xarope (combo)	7,1	7,1	100	1,0 (1,0-1,0)	0,0000	1,0 (1,0-1,0)	-	7,1	7,1	100	1,0 (1,0-1,0)	0,0000	1,0 (1,0-1,0)	-
Açaí sem xarope (promoção)	0	0	100	-	-	-	-	0	0	100	-	-	-	-
Bolo de preparação culinária (comercializado)	50,0	50,0	100	1,0 (1,0-1,0)	0,0001	1,0 (1,0-1,0)	-	50,0	50,0	100	1,0 (1,0-1,0)	0,0001	1,0 (1,0-1,0)	-
Bolo de preparação culinária (variedade)*	1,8 (0,6)	1,8 (0,6)	100	1,0 (1,0-1,0)	0,0000	-	-	1,8 (0,6)	1,8 (0,6)	100	1,0 (1,0-1,0)	0,0000		-
Bolo de preparação culinária (tamanho)*	36,2 (20,8)	36,2 (20,8)	100	1,0 (1,0-1,0)	0,0000	-	-	36,2 (20,8)	36,2 (20,8)	100	1,0 (1,0-1,0)	0,0000		-
Bolo de preparação culinária (preço) *	3,7 (0,9)	3,6 (1,0)	85,7	0,9 (0,9-0,9)	0,0000	-	-	3,7 (0,9)	3,7 (0,9)	100	1,0 (1,0-1,0)	0,0000		-
Bolo de preparação culinária (combo)	7,1	7,1	100	1,0 (1,0-1,0)	0,0000	1,0 (1,0-1,0)	-	7,1	7,1	100	1,0 (1,0-1,0)	0,0000	1,0 (1,0-1,0)	-
Bolo de preparação culinária (promoção)	0	0	100	-	-	-	-	0	0	100	-	-	-	-

Biscoito preparação culinária (comercializado)	14,2	14,2	100	1,0 (1,0-1,0)	0,0001	1,0 (1,0-1,0)	-	14,2	14,2	100	1,0 (1,0-1,0)	0,0001	1,0 (1,0-1,0)	-
Biscoito preparação culinária (variedade)*	1,0 (0,0)	1,0 (0,0)	100	-	-	-	-	1,0 (0,0)	1,0 (0,0)	100	-	-		-
Biscoito preparação culinária (tamanho)*	27,0 (23,0)	27,0 (23,0)	100	1,0 (1,0-1,0)	-	-	-	27,0 (23,0)	27,0 (23,0)	100	1,0 (1,0-1,0)	-		-
Biscoito preparação culinária (preço)*	3,8 (0,5)	3,8 (0,5)	100	1,0 (1,0-1,0)	0,0000	-	-	3,8 (0,5)	3,8 (0,5)	1	1,0 (1,0-1,0)	0,0000		-
Biscoito preparação culinária (combo)	7,1	7,1	100	1,0 (1,0-1,0)	0,0000	1,0 (1,0-1,0)	-	7,1	7,1	100	1,0 (1,0-1,0)	0,0000	1,0 (1,0-1,0)	-
Biscoito preparação culinária (promoção)	0	0	100	-	-	-	-	0	0	100	-	-	-	-
Tapioca sem recheio UP (comercizalizado)	0	0	100	-	-	-	-	0	0	100	-	-	-	-
Pizza sem recheio UP (comercializado)	78,5	78,5	100	1,0 (1,0-1,0)	0,0001	1,0 (1,0-1,0)	-	78,5	78,5	100	1,0 (1,0-1,0)	0,0001	1,0 (1,0-1,0)	-
Pizza sem recheio UP (variedade)*	1,1 (0,4)	1,0 (0,3)	90,9	0,7 (0,1-0,9)	0,0120	-	-	1,1 (0,4)	1,1 (0,4)	100	1,0 (1,0-1,0)	-		
Pizza sem recheio UP (tamanho)*	1 (0,0)	1 (0,0)	100	-	-	-	-	1,0	1,0	100	-	-		
Pizza sem recheio UP (preço)*	4,8 (1,3)	4,8 (1,3)	100	1,0 (1,0-1,0)	-	-	-	4,8 (1,3)	4,9 (1,2)	90,9	0,9 (0,9-0,9)	0,0000		
Pizza sem recheio UP (combo)	7,1	14,2	92,8	0,8 (0,5-1,0)	0,0000	0,8 (0,6-1,0)	0	7,1	7,1	100	1,0 (1,0-1,0)	0,0000	1,0 (1,0-1,0)	-
Pizza sem recheio UP (promoção)	0	0	100	-	0,0001	-	-	0	0	100	-	-	-	-
Salgado assado sem recheio UP (comercializado)	78,5	85,7	92,8	0,7 (0,3-1,0)	0,0017	0,8 (0,5-1,0)	0	78,5	85,7	92,8	0,7 (0,3-1,0)	0,0017	0,8 (0,5-1,0)	0
Salgado assado sem recheio UP (variedade)*	2,4 (2,3)	2,3 (2,2)	100	1,0 (1,0-1,0)	0,0000	-	-	2,4 (2,3)	2,3 (2,2)	100	1,0 (1,0-1,0)	0,0000		
Salgado assado sem recheio UP (tamanho)*	1,0 (0,0)	1,0 (0,0)	100	-	-	-	-	1,0 (0,0)	1,0 (0,0)	100	-	-		
Salgado assado sem recheio UP (preço)*	4,2 (1,1)	4,1 (1,1)	92,8	1,0 (1,0-1,0)	0,0000	-	-	4,2 (1,1)	4,1 (1,1)	92,8	1,0 (1,0-1,0)	0,0000		
Salgado assado sem recheio UP (combo)	7,1	14,2	85,7	0,8 (0,7-1,0)	0,0003	0,7 (0,4-1,0)	0	7,1	7,1	92,8	1,0 (1,0-1,0)	0,0000	0,8 (0,6-1,0)	0
Salgado assado sem recheio UP (promoção)	0	0	100	-	-	-	0	0	0	100	-	-	-	-
Salgado frito sem recheio UP (comercializado)	14,2	14,2	100	1,0 (1,0-1,0)	0,0001	1,0 (1,0-1,0)	-	14,2	14,2	100	1,0 (1,0-1,0)	0,0001	1,0 (1,0-1,0)	-
Salgado frito sem recheio UP (variedade)*	1,0 (0,0)	1,0 (0,0)	100	-	-	-	-	1,0 (0,0)	1,0 (0,0)	100	-	-	-	-
Salgado frito sem recheio UP (tamanho)*	1,0 (0,0)	1,0 (0,0)	100	-	-	-	-	1,0 (0,0)	1,0 (0,0)	100	1,0 (1,0-1,0)	-	-	-
Salgado frito sem recheio UP (preço)*	5,5 (0,7)	5,5 (0,7)	100	1,0 (1,0-1,0)	-	-	-	5,5 (0,7)	5,5 (0,7)	100	1,0 (1,0-1,0)	-	-	-
Salgado frito sem recheio UP (combo)	0	0	100	-	-	-	-	0	0	100	-	-	-	-
Salgado frito sem recheio UP (promoção)	0	0	100	-	-	-	-	0	0	100	-	-	-	-
Sanduíche sem recheio UP (comercializado)	35,7	35,7	100	1,0 (1,0-1,0)	0,0001	1,0 (1,0-1,0)	-	35,7	35,7	100	1,0 (1,0-1,0)	0,0001	1,0 (1,0-1,0)	-
Sanduíche sem recheio UP (variedade)*	1,0 (0,0)	1,0 (0,0)	100	-	-	-	-	1,0 (0,0)	1,0 (0,0)	100	-	-	-	-
Sanduíche sem recheio UP (tamanho)*	1,0 (0,0)	1,0 (0,0)	100	-	-	-	-	1,0 (0,0)	1,0 (0,0)	100	-	-	-	-
Sanduíche sem recheio UP (preço)*	5,8 (0,9)	5,8 (0,9)	100	1,0 (1,0-1,0)	0,0000	-	-	5,8 (0,9)	5,8 (0,9)	100	1,0 (1,0-1,0)	0,0000		-
Sanduíche sem recheio UP (combo)	0	0	100	-	-	-	-	0	0	100	-	-	-	-
Sanduíche sem recheio UP (promoção)	0	0	100	-	-	-	-	0	0	100	-	-	-	-

Legenda: * para as variáveis quantitativas foram calculadas a média (dp) e o Coeficiente de Correlação Intraclasse (CCI)

Tabela 4. Itens do questionário que não se aplicavam à avaliação da confiabilidade interobservador e intraobservador

Itens
Refrigerante zero (variedade)*
Refrigerante zero (tamanho)*
Refrigerante zero (preço)*
Refrigerante zero (combo)
Refrigerante zero (promoção)
Energético (variedade)*
Energético (tamanho)*
Energético (preço)*
Energético (combo)
Energético (promoção)
Cereal matinal (variedade)*
Cereal matinal (tamanho)*
Cereal matinal (preço)*
Cereal matinal (combo)
Cereal matinal (promoção)
Salada de frutas com toppings (variedade)*
Salada de frutas com toppings (tamanho)*
Salada de frutas com toppings (preço)*
Salada de frutas com toppings (combo)
Salada de frutas com toppings (promoção)
Tapioca com recheio UP (variedade)*
Tapioca com recheio UP (tamanho)*
Tapioca com recheio UP (preço)*
Tapioca com recheio UP (combo)
Tapioca com recheio UP (promoção)
Salgado frito com recheio UP (variedade)*
Salgado frito com recheio UP (tamanho)*
Salgado frito com recheio UP (preço)*
Salgado frito com recheio UP (combo)
Salgado frito com recheio UP (promoção)
Pipoca UP (variedade)*
Pipoca UP (tamanho)*
Pipoca UP (preço)*
Pipoca UP (combo)
Pipoca UP (promoção)
Chá natural (variedade)*
Chá natural (tamanho)*
Chá natural (preço)*
Chá natural (combo)
Chá natural (promoção)
Fruta seca/desidratada (variedade)
Fruta seca/desidratada (tamanho)
Fruta seca/desidratada (preço)

Fruta seca/desidratada (combo)
Fruta seca/desidratada (promoção)
Tapioca sem recheio UP (variedade)*
Tapioca sem recheio UP (tamanho)*
Tapioca sem recheio UP (preço)*
Tapioca sem recheio UP (combo)
Tapioca sem recheio UP (promoção)
