# Supplementary material for: Development, content validity, and reliability of an instrument to assess the commercialization of food and beverages in school canteens in Brazil
Source: Rev Bras Epidemiol. 2026 Apr 3;29:e260015. doi: 10.1590/1980-549720260015 (PMC13053033; doi:10.1590/1980-549720260015)
Supplement: Supplementary file 2 [file 1980-5497-rbepid-29-e260015-sppl1.pdf]

MANUAL PARA COLETA DE DADOS

“Comercialização de Alimentos em Escolas Brasileiras”

Dados Internacionais de Catalogação na Publicação (CIP)
(Câmara Brasileira do Livro, SP, Brasil)

Manual para coleta de dados [livro eletrônico] : "Comercialização de Alimentos em Escolas Brasileiras" / [coordenação e elaboração do texto Larissa Loures Mendes...[et al.]]. -- Belo Horizonte, MG : Ed. dos Autores, 2024. PDF

Outros autores: Raquel Canuto, Letícia Ferreira Tavares, Letícia de Oliveira Cardoso, Paulo César Pereira de Castro Júnior, Luiza Delazari Borges, Sabrina Gomes Ferreira Clark.
Bibliografia.
ISBN 978-65-01-23104-4

1. Alimentação - Aspectos sociais 2. Alimentação - Cuidados e higiene 3. Alimentos - Comércio 4. Alimentos - Qualidade 5. Ambiente escolar 6. Dados - Análise 7. Método de estudo 8. Segurança alimentar 9. Serviços de alimentação I. Mendes, Larissa Loures. II. Canuto, Raquel. III. Tavares, Letícia Ferreira. IV. Cardoso, Letícia de Oliveira. V. Castro Júnior, Paulo César Pereira de. VI. Borges, Luiza Delazari. VII. Clark, Sabrina Gomes Ferreira. VIII. Mendes, Larissa Loures.

24-239145 CDD-371.7160981

Índices para catálogo sistemático:

1. Brasil : Alimentos e bebidas : Ambiente escolar : Educação 371.7160981

Eliete Marques da Silva - Bibliotecária - CRB-8/9380

EQUIPE TÉCNICA

EQUIPE COORDENADORA

Larissa Loures Mendes

Raquel Canuto

Letícia Ferreira Tavares

Letícia de Oliveira Cardoso

Paulo César Pereira de Castro Júnior

Luiza Delazari Borges

Sabrina Gomes Ferreira Clark

ELABORAÇÃO DO TEXTO

Larissa Loures Mendes

Raquel Canuto

Letícia Ferreira Tavares

Letícia de Oliveira Cardoso

Paulo César Pereira de Castro Júnior

Luiza Delazari Borges

Sabrina Gomes Ferreira Clark

1. APRESENTAÇÃO

Pensando em avaliar a comercialização de alimentos realizada pelas cantinas das escolas e caracterizar o comércio de rua no entorno imediato das escolas de cidades brasileiras, idealizou-se a realização do estudo intitulado **"Comercialização de alimentos em escolas brasileiras - Caeb"** que está sendo desenvolvido pela UFMG em parceria com a UFRGS, UFRJ, Fiocruz, UFC e UFPE. Esse manual visa, dessa forma, orientar a coleta de dados e esclarecer dúvidas dos pesquisadores envolvidos.

O(A) pesquisador(a) de campo, em qualquer investigação, possui importante papel, ao considerar que todos os dados por ele(a) coletados serão utilizados como base para posteriores análises da pesquisa. Por esse motivo, destaca-se a importância de seu trabalho e a seriedade de sua atitude para o sucesso da pesquisa.

O(A) pesquisador(a) deve buscar obter dados fidedignos, e para isso, realizar suas funções de forma tranquila e paciente e não influenciar as respostas dos entrevistados(as) por meio de suas atitudes ou comentários, e seguir rigorosamente as instruções deste manual. Outro ponto relevante é que o estudo está ocorrendo em várias cidades ao mesmo tempo e a qualidade da coleta de dados precisa ser a mesma em todos os locais de coleta, e também por isso, a padronização na forma de coletar os dados é fundamental. Neste sentido, este manual irá detalhar o trabalho de campo e será fonte permanente de consulta e orientação para conduzir adequadamente a coleta de dados, do estudo Caeb. Ele contemplará os instrumentos de coleta de dados que serão utilizados, além de questões de logísticas da coleta. Leia-o atentamente, visando contribuir para a coleta de dados fidedignos.

Em resumo, esse manual possui os seguintes objetivos: (a) padronizar e orientar a coleta de dados de maneira geral; (b) esclarecer possíveis dúvidas de preenchimento do questionário e facilitar a adequada coleta de dados; (c) orientar a abordagem aos responsáveis/colaboradores das cantinas e vendedores(as) de comércio de rua, (d) reduzir e eliminar a ocorrência de erros durante a coleta objetivando a obtenção de dados confiáveis.

Agradecemos a sua colaboração.

1. COLETA DE DADOS

Esta coleta de dados consistirá em observação das cantinas e do comércio ambulante no entorno das escolas, bem como entrevista com responsáveis/colaboradores das cantinas e com os vendedores ambulantes na porta de escolas privadas de cidades brasileiras.Há questões, nos instrumentos de coleta, que poderão ser respondidas por meio de observação do espaço e outras que precisarão de diálogo com o indivíduo responsável pelo local de venda de alimentos.

1.1. Abordagem

a. *Seja proativo e comunicativo.* Cumprimente o(a) entrevistado(a) com “Bom dia, como você está?”, “Boa tarde, tudo bem com você?”.

b. *Se apresente.* É fundamental que você se apresente falando sua função e qual empresa/universidade você representa na etapa de coleta, qual seu objetivo ali e qual a justificativa para estar fazendo este trabalho. Exemplo: *“Meu nome é _______ e trabalho para a Vox Populi, uma empresa que pesquisa a opinião pública. Sua opinião é muito importante para nós. Hoje estamos fazendo uma pesquisa sobre a comercialização de alimentos e bebidas nas cantinas e no entorno das escolas particulares em várias cidades do país. Garantimos que suas respostas são totalmente confidenciais e serão somadas às de outras pessoas, para apuração dos resultados. Elas nunca são tratadas individualmente, nem sua identidade ou opinião divulgada. Caso não saiba ou não queira responder alguma questão, não tem problema. É só falar que não quer ou não sabe responder que registramos sua resposta. Você poderia colaborar com o nosso trabalho respondendo a pesquisa?”*

c. Ao perguntar se há o desejo de *“colaborar com o nosso trabalho”*, **se a resposta do(a) entrevistado(a) for “Sim”, marque “Sim” e aplique o questionário.** Essa terá sido a resposta ao termo de consentimento livre e esclarecido (TCLE) e uma cópia impressa do TCLE (dos resumo e dados de contato) deve ser entregue ao(a) entrevistado(a).

d. **Caso haja recusa, marque “Não”, marque “Não” e substitua o(a) entrevistado(a)**. Essa terá sido a resposta ao termo de consentimento livre e esclarecido (TCLE) e uma cópia impressa do TCLE (dos resumo e dados de contato) deve ser entregue ao(a) entrevistado(a).

e. *Sorria.* Demonstre empatia e educação, mesmo de máscara se for a realidade local segundo as normas sanitárias municipais.

f. Agradeça ao final das perguntas da seção.

1.2. Local de coleta

Vamos coletar os dados nas cantinas das escolas, preferencialmente, por meio do(a) gestor(a)/funcionário (a) da cantina e do comércio ambulante com todos os indivíduos que estiverem comercializando alimentos na porta das escolas (calçada que circunda toda a escola) e passeio/calçada do outro lado a rua em que a escola está situada (voltados à fachada da escola). **Para a abordagem nas cantinas, evite os horários do recreio (9:30-10:30 e 15:00-16:00)** pois é quando pode haver maior demanda de trabalho para o(a) entrevistado(a).

1.3. Check List de itens para levar para o campo

- Manual para coleta de dados
- Tablets/celular
- Planilha de endereços das escola
- Crachá
- Termo de Consentimento Livre Esclarecido (TCLE) impresso (cópia do(a) entrevistado(a)

1.4. Check List de atividades em campo (cantinas escolares)

a. Identifique-se à segurança/portaria/recepção da escola conforme a autorização de entrada dada pelo dono ou gestor da cantina, quando a cantina for administrada por empresa terceirizada, na fase de contato telefônico com (o)a supervisor(a) do campo. Em escolas que fazem a autogestão da cantina, a autorização de entrada terá sido dada pela direção/membro da secretaria escolar.

b. Apresente-se ao (a) segurança/recepcionista da escola e solicite o direcionamento ao(a) gestor(a)/funcionário(a) da cantina que será entrevistado(a).

c. Apresente-se ao(a) gestor(a)/funcionário(a) da cantina solicitando permissão para observar os produtos disponíveis e cardápio (se houver), segundo a abordagem que consta no tópico 1.1 deste manual.

d. Certifique-se de preencher o cabeçalho com o município, nome da escola, localização do(a) ambulante no momento da coleta de dados como responsável/proprietário(a) da cantina.

e. Observe e registre as informações referentes aos alimentos comercializados, perguntando:

   - *Esta cantina comercializa (insira aqui o nome dos itens da nossa lista).*

f. Pergunte ao(a) entrevistado(a) sobre os sabores de recheio de alguns alimentos quando o questionário demandar essa informação e não houver etiquetas com essa descrição.

g. Observe e registre as informações referentes à publicidade dos alimentos comercializados, conforme a seção.

h. Solicite o nº de telefone, endereço do(a) entrevistado(a) para checagem dos dados, se for preciso.

i. **Solicite autorização para fotografar o cardápio (se houver), o espaço da cantina (sem captar a identidade da escola ou indivíduos) e os materiais publicitários (se houver).**

j. Verifique se todos os itens do questionário foram preenchidos.

ATENÇÃO: Em caso de recusa do(a) vendedor(a), registre a resposta de recusa e siga para outro candidato è eleição.

1.6 Check List de atividades em campo (ambulantes)

a. Identifique os(as) vendedores(as) de rua no entorno das escolas **assim que chegar** para o encontro marcado dentro da escola. Se localizar algum, proceda a abordagem. **Ao sair do interior da escola** volte a verificar se há outros ambulantes no entorno da escola, se houver novos, proceda a abordagem.

b. Aborde um de cada vez e apresente-se solicitando permissão para observar os produtos disponíveis e cardápio (se houver), segundo a abordagem que consta no tópico 1.1 deste manual.

c. Certifique-se de preencher o cabeçalho com o município, nome da escola (mesmo para ambulantes), localização do(a) ambulante (na porta da escola ou no quarteirão) no momento da coleta de dados com ele.

d. Lembre-se de marcar a opção que diz respeito ao local (no quarteirão ou na porta da escola) em que vocês se encontraram.

e. Observe e registre as informações referentes aos alimentos comercializados, conforme a seção 2.2 do questionário.

f. Observe e registre as informações referentes aos alimentos comercializados, perguntando: - *Esta cantina comercializa __________?* (insira aqui o nome dos itens da nossa lista).

g. Pergunte ao(a) vendedor(a) sobre os sabores de recheio de alguns alimentos quando o questionário demandar essa informação e não houver etiquetas com essa descrição.

h. Observe e registre as informações referentes à publicidade dos alimentos comercializados, conforme a seção 2.3 do questionário.

i. Solicite o nº de telefone, endereço do(a) entrevistado(a) para checagem dos dados, se for preciso.

j. **Solicite autorização para fotografar o cardápio (se houver), o espaço do comércio ambulante (sem captar a identidade da escola ou indivíduos) e os materiais publicitários (se houver).**

k. Verifique se todos os itens do questionário foram preenchidos.

l. Dirija-se para o(a) próximo(a) vendedor(a) ambulante (se houver).

ATENÇÃO: Em caso de recusa do(a) vendedor(a), registre a resposta de recusa e siga para outro candidato è eleição.

2. INSTRUMENTO PARA AVALIAÇÃO DAS INFORMAÇÕES DA CANTINA – SEÇÃO 1

Preencher adequadamente o cabeçalho.

2.2 IDENTIFICAÇÃO E CARACTERIZAÇÃO DA CANTINA - SEÇÃO 1.1

As questões desse bloco devem ser respondidas pela pessoa que estiver trabalhando na cantina no momento da coleta de dados, seja o responsável pela cantina ou um funcionário.

Questão 6: Nome do responsável legal/proprietário do estabelecimento

Questão 7: Número médio de clientes atendidos por dia:
Nessa questão a resposta é aberta, pergunte ao(a) entrevistado(a) “Em média, quantos clientes são atendidos por dia pela cantina?” e digite a resposta dele(a) no local determinado.

Questão 8: Responsável pela administração da cantina
No caso de existir um responsável diferente das opções listadas, assinale o campo correspondente a “outra” e escreva qual é a outra administração.

Questão 9: Número de funcionários da cantina
Pergunte ao(a) entrevistado(a) “Quantos funcionários trabalham na cantina?” e preencha somente uma das opções apresentadas.

Questão 10: Presença de nutricionista na cantina.
Pergunte ao(a) entrevistado(a) “A cantina tem nutricionista?”. Assinale “Sim” ou “Não” de acordo com a resposta obtida.

Questão 11: Refeições ofertadas.
Pergunte ao(a) entrevistado(a) “Quais os tipos de refeições ofertadas aos alunos?” Leia as opções do questionário para ele(a) e assinale “Sim” ou “Não” para cada refeição no questionário, de acordo com a resposta obtida.

Questão 12: Produção das preparações culinárias comercializadas
Pergunte ao(a) entrevistado(a) “*Onde são produzidos as preparações culinárias comercializadas na cantina?”* Leia as opções do questionário para ele(a) e assinale “Sim” ou “Não” para cada opção de local no questionário, de acordo com a resposta obtida. E diga “Se houver outro local de produção além dos citados aqui, também pode me dizer”. Assinale e preencha a opção “Outros” se ele(a) informar outro local de produção além dos indicados para assinalar.

Questão 13: Disponibilidade dos cardápios das refeições para os clientes:
Pergunte ao(a) entrevistado(a) “*Os cardápios das refeições (café da manhã, almoço, lanche, jantar) estão disponíveis para os clientes?”* Leia as opções do questionário para ele(a) e assinale “Sim” ou “Não” para cada opção de local no questionário, de acordo com a resposta obtida.Caso a opção de resposta escolhida pelo

entrevistado seja “não”, pule para a questão 15.

<u>Atenção: Pedir ao responsável pela cantina uma cópia do cardápio ou pedir para tirar uma foto do mesmo.</u>

Questão 14: Forma dos cardápios disponibilizados para os clientes:

Pergunte ao(a) entrevistado(a) “*Como os cardápios das refeições (café da manhã, almoço, lanche, jantar) estão disponíveis para os clientes?”* Leia as opções do questionário para ele (a), e assinale “Sim” ou “Não” para cada opção de local no questionário. E diga “Se houver outra forma de disponibilizar os cardápios além das citadas aqui, também pode me dizer”. Assinale e preencha a opção “Outros” se ele(a) informar outro local de produção além dos indicados para assinalar.

Tipo de cardápio	Caracterização	Exemplo
Impresso (entregue ao cliente)	lista de preparações culinárias que compõe uma refeição ou lista de preparações que compõem todas as refeições de um dia ou período determinado disponibilizado de forma impressa ao cliente	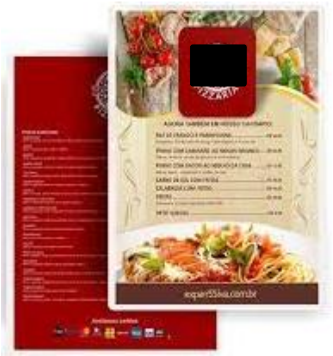 [redacted]<br>Fonte: Google imagens.
Display de mesa	lista de preparações culinárias que compõe uma refeição ou lista de preparações que compõem todas as refeições de um dia ou período determinado em formato de estrutura de material leve (ex. papel cartão, acrílico, vinil) que se mantêm sobre a mesa/balcão	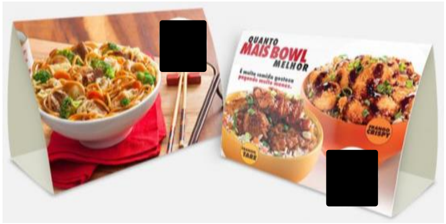 [redacted] [redacted]<br>Fonte: Google imagens.
Banner/totem/TV	Banner: peça publicitária em forma de bandeira, confeccionada em plástico, tecido ou papel, impressa de um ou de ambos os lados, ger. para ser pendurada em postes, fachadas ou paredes, exposta na via pública, em pavilhões de exposições, pontos de venda etc<br><br>Totem: um terminal conectado ao sistema de atendimento da loja, compartilhando o mesmo banco de dados de produtos e vendas, com o qual o cliente interage para fazer seu	Banner: 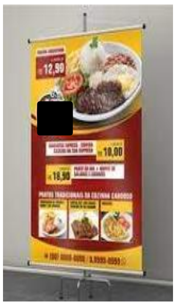 [redacted]<br>Fonte: Google imagens.<br><br>Totem: 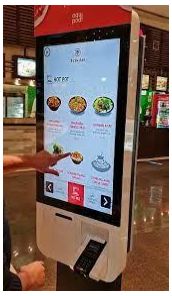<br>Fonte: Google imagens.

	pedido, sem interação com agentes humanos.<br><br>TV: um sistema de transmissão de imagens e som à distância através de ondas hertzianas. No caso da televisão por cabo, a transmissão é feita através de uma rede especializada.	TV:<br>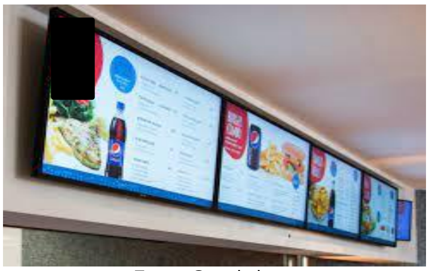<br>Fonte: Google imagens.
Internet/QR code	O cardápio QR Code permite que os clientes visualizem o cardápio do estabelecimento em seu próprio celular, dentro do estabelecimento. Eles podem ver fotos dos pratos, conferir seus preços e saber mais sobre cada um deles lendo suas descrições.	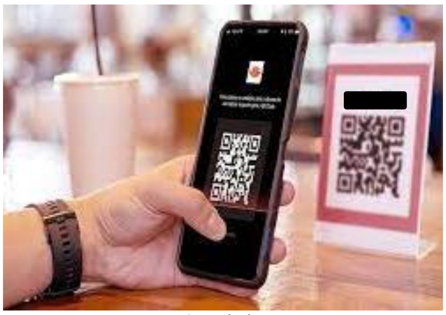<br>Fonte: Google imagens.
No cardápio impresso disponível fora da cantina	lista de preparações culinárias que compõe uma refeição ou lista de preparações que compõem todas as refeições de um dia ou período determinado disponibilizado de forma impressa ao cliente **em espaços fora da cantina** como corredores, murais em salas de aula, sala de professores, secretaria etc.	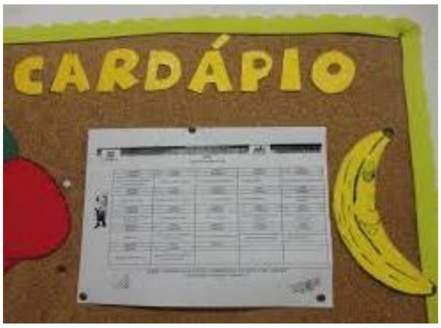<br><br>Fonte: Google imagens.
Na bandeja/no próprio produto	O Jogo Americano ou Papel Bandeja, é um elemento que além de poder mostrar a lista de preparações culinárias, pode transmitir informações diversas e facilitar a limpeza das mesas.	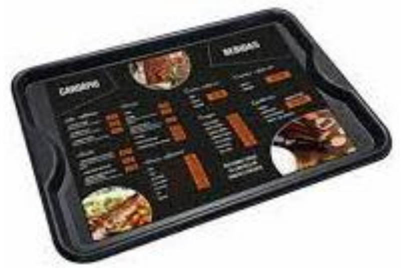<br>Fonte: Google imagens.
Aplicativo da escola/cantina	Por meio do download de um aplicativo pode-se consultar o cardápio da cantina	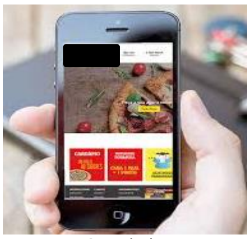<br>Fonte: Google imagens.

Questão 15: Disponibilidade do preço das refeições para os clientes:

Pergunte ao(a) entrevistado(a) "*Os preços são disponibilizados para os clientes?"* Leia as opções do questionário para ele(a) e assinale "Sim" ou "Não", de acordo com a resposta obtida. Caso a opção de resposta escolhida pelo entrevistado seja "não", pule para a questão 17.

Questão 16: Forma em que os preços são disponibilizados para os clientes:

Pergunte ao(a) entrevistado(a) "*Como os preços estão disponíveis para os clientes?"* Leia as opções do questionário para ele (a), e assinale "Sim" ou "Não" para cada opção de local no questionário. E diga "Se houver outra forma de disponibilizar os preços, além das citadas aqui, também pode me dizer". Assinale e preencha a opção "Outros" se ele(a) informar outro local de produção além dos indicados para assinalar.

Questão 17: Disponibilidade das informações alimentares e nutricionais das preparações culinárias que compõem o cardápio:

Pergunte ao(a) entrevistado(a) "As informações alimentares e nutricionais (ex: composição nutricional/ingredientes, calorias (kcal), carboidratos, proteínas, açúcar, gordura) das preparações culinárias que compõem o cardápio estão disponíveis para os clientes?" Leia as opções do questionário para ele(a) e assinale "Sim, para todos", "Sim, para parte deles" ou "Não", de acordo com a resposta obtida.

Questão 18: Comercialização de alimentos para fins especiais:

Pergunte ao(a) entrevistado(a) "A cantina comercializa alimentos para clientes que possuem alguma particularidade (ex: alergia, intolerância, diabetes)? Leia as opções do questionário para ele(a) e assinale "Sim" ou "Não", de acordo com a resposta obtida.

Questão 19: Presença de estrutura como mesa/balcão para consumo de lanche ou refeição no local:

Pergunte ao(a) entrevistado(a) "Na cantina ou próximo da cantina há estrutura como mesa/balcão para consumo de lanche ou refeição no local? Leia as opções do questionário para ele(a) e assinale "Sim" ou "Não", de acordo com a resposta obtida.

Questão 20: Presença de açúcar disponível na mesa/balcão:

Pergunte ao(a) entrevistado(a) "Na cantina há açúcar disponível na mesa/balcão?" Leia as opções do questionário para ele(a) e assinale "Sim" ou "Não", de acordo com a resposta obtida.

Questão 21: Presença de adoçante disponível na mesa/balcão:

Pergunte ao(a) entrevistado(a) "Na cantina há adoçante disponível na mesa/balcão?" Leia as opções do questionário para ele(a) e assinale "Sim" ou "Não", de acordo com a resposta obtida.

Questão 22: Presença de sal na mesa/balcão:

Pergunte ao(a) entrevistado(a) "Na cantina há oferta de sal na mesa/balcão?" Leia as opções do questionário para ele(a) e assinale "Sim" ou "Não", de acordo com a resposta obtida.

Questão 23: Oferta de maionese, mostarda, catchup e outros molhos ultraprocessados na mesa/balcão:

Pergunte ao(a) entrevistado(a) "Na cantina há oferta de maionese, mostarda, catchup e outros molhos ultraprocessados na mesa/balcão?" Leia as opções do questionário para ele(a) e assinale "Sim" ou "Não", de

acordo com a resposta obtida.

Questão 24: Critério (s) para seleção dos alimentos comercializados:
Pergunte ao(a) entrevistado(a) “Com base em que você seleciona os alimentos comercializados?” Leia as opções do questionário para ele (a), e assinale “Sim” ou “Não” para cada opção de local no questionário. E diga “Se houver outro (s) critério (s) de seleção dos alimentos além dos citados aqui, também pode me dizer”. Assinale e preencha a opção “Outros” se ele(a) informar outro critério além dos indicados para assinalar.

Questão 25: Formas de pagamento:
Pergunte ao(a) entrevistado(a) “Quais as formas de pagamento na cantina?” Leia as opções do questionário para ele (a), e assinale “Sim” ou “Não” para cada opção de local no questionário. E diga “Se houver outra (s) forma (s) de pagamento além das citadas aqui, também pode me dizer”. Assinale e preencha a opção “Outros” se ele (a) informar outra(s) formas de pagamento.

Questão 26: Recebimento de material de incentivo/patrocínio/apoio de fornecedores dos produtos comercializados:
Pergunte ao(a) entrevistado(a) “A cantina já recebeu/recebe algum material de incentivo/patrocínio/apoio de fornecedores dos produtos que comercializa? Como por exemplo freezer, mesas, porta guardanapo, isopor etc.”. Assinale “Sim” ou “Não” de acordo com a resposta obtida. Ainda que a resposta seja “Não”, observe e assinale a presença de materiais descritos na questão 27.

Questão 27: Materiais de incentivo/patrocínio/apoio de fornecedores que a cantina costuma utilizar:

Observe as opções de materiais listados no questionário e assinale “Sim” quando identificar sua presença. Quando não encontrar alguma das opções, pergunte ao(a) entrevistado(a) se há a presença do determinado item não encontrado e assinale “Sim ou Não” conforme o(a) entrevistado(a) informar. Você pode perguntar *“Há algum brinde de marcas de alimentos como chaveiros, canetas, imãs de geladeira por exemplo?”*.

Questão 28: Empresas patrocinadoras dos materiais da cantina:
Apenas SE você houver identificado materiais listados na questão 27, digite no local destinado quais empresas patrocinadoras foram identificadas. Como por exemplo: Coca-cola, Elma-Chips etc

Questão 29: Ações da cantina para a alimentação saudável:
Pergunte ao(a) entrevistado(a) “ A cantina desenvolve ações que incentivem a alimentação saudável?” Leia as opções do questionário para ele (a), e assinale “Sim” ou “Não” de acordo com a resposta obtida. Caso a opção de resposta escolhida pelo entrevistado seja “sim” pergunte “Quais ações são essas?” e digite a resposta no local destinado.

Questão 30: Presença de materiais educativos (murais, cartazes, banners, quadros e etc) sobre alimentação saudável na cantina:
Atenção: Essa questão deve ser OBSERVADA pelo pesquisador de campo

Após a observação do espaço, o pesquisador de campo deve assinalar “Sim” ou “Não” em relação a presença de materiais educativos (murais, cartilhas, cartazes, banners, quadros e etc) sobre alimentação saudável. Caso a opção de resposta escolhida pelo entrevistado (a) seja “não”, pule para a questão 32.

Questão 31: Elaboração dos materiais educativos:
Pergunte ao(a) entrevistado(a) “Por quem esses materiais educativos foram elaborados?” Leia as opções do questionário para ele (a), e assinale “Sim” ou “Não” de acordo com a resposta obtida. E diga “Se houver fonte ou identificação da elaboração desses materiais além das citadas aqui, também pode me dizer”. Assinale e preencha a opção “Outros” se ele (a) informar outra resposta.

Questão 32: Proibição da venda de algum tipo de alimento/produto pela cantina:
Pergunte ao(a) entrevistado(a) “A direção da escola proíbe a venda de algum tipo de alimento/produto pela cantina?” Leia as opções do questionário para ele (a), e assinale “Sim” ou “Não” de acordo com a resposta obtida. Caso a opção de resposta escolhida pelo entrevistado seja “sim” pergunte “Quais alimentos a direção da escola proíbe?” e digite a resposta no local destinado.

Questão 33: Presença de refeição (comida) na cantina:
Pergunte ao(a) entrevistado(a) “A cantina oferece refeição (comida)? Leia as opções do questionário para ele (a), e assinale “Sim” ou “Não” de acordo com a resposta obtida. Caso a opção de resposta escolhida pelo entrevistado (a) seja “não”, pule para a questão 35.

Questão 34: Menor preço da refeição oferecida pela cantina:
Pergunte ao(a) entrevistado(a) “. Qual o menor preço da refeição oferecida pela cantina? Tanto para o prato feito quanto do quilo” e digite a resposta dele(a) no local determinado.

Questão 35: Combo comercializado na cantina:
Pergunte ao(a) entrevistado(a) “A cantina oferece opção de combo (combinado de alimentos com bebidas) com preço final inferior ao da soma dos preços individuais de cada componente da combinação?” Leia as opções do questionário para ele (a), e assinale “Sim” ou “Não” de acordo com a resposta obtida.

Questão 36: Promoção de alimentos e bebidas na cantina:
Pergunte ao(a) entrevistado(a) “A cantinas faz promoção de alimentos e bebidas?” Leia as opções do questionário para ele (a), e assinale “Sim” ou “Não” de acordo com a resposta obtida.

Questão 37: Venda de alimentos e bebidas por outras pessoas da comunidade escolar:
Pergunte ao(a) entrevistado(a) “Há venda de alimentos e bebidas por outras pessoas da comunidade escolar (ex: alunos, professores, pais de alunos)?” Leia as opções do questionário para ele (a), e assinale “Sim” ou “Não” de acordo com a resposta obtida.

INSTRUÇÕES PARA AS PRÓXIMAS SEÇÕES (1.2, 1.3, 2.2, 2.3)

O Guia Alimentar para a População Brasileira define as diretrizes a serem utilizadas na orientação de escolhas alimentares saudáveis e adota a classificação NOVA que agrupa os alimentos segundo o nível e objetivo de processamento deles. Relembre a definição das classes de alimentos segundo a NOVA:

Alimentos *in natura*	Aqueles extraídos diretamente de plantas e animais e adquiridos para consumo, sem que passem por alterações após retirados da natureza.	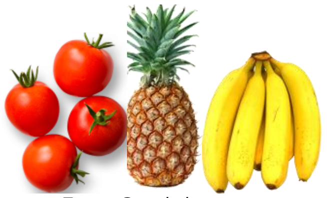 Fonte: Google imagens.
Alimentos minimamente processados	Aqueles alimentos in natura que, antes de serem adquiridos, passaram por alterações mínimas (lavagem, corte, trituração, moagem, congelamento, pasteurização, vácuo etc.) e nenhuma adição de outros componentes.	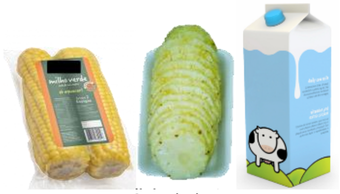 Fonte: Google imagens.
Alimentos processados	Aqueles produtos à base de alimentos *in natura* ou minimamente processados com a adição de açúcar, sal, vinagre ou gorduras para o aumento da durabilidade.	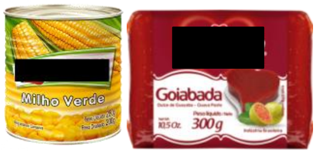 Fonte: Google imagens.
Alimentos ultraprocessados	Aqueles produzidos exclusivamente em indústrias com a adição de substâncias artificiais ou componentes que não são considerados alimentos (gordura hidrogenada, amido modificado, proteína de soja, aromatizante, emulsificante, adoçante etc.) para alteração de sabor, aroma, cor e aumento da durabilidade.	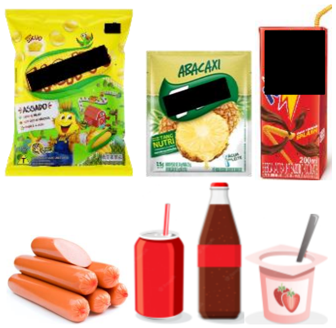 Fonte: Google imagens.
Ingredientes culinários processados	Substâncias extraídas diretamente da natureza e utilizadas para temperar os alimentos e criar preparações culinárias. Nunca são consumidos isoladamente.	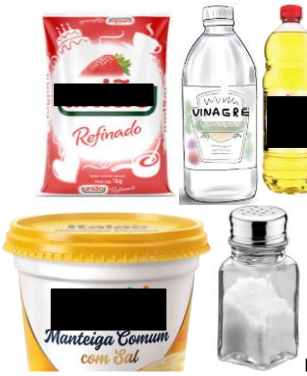 Fonte: Google imagens.

Você deve relembrar essa definição sempre que houver dúvidas quanto à lista de alimentos das seções 1.2 e 2.2, visto que alguns alimentos são diferenciados segundo a classificação acima e devemos prevenir erros. Se houver dúvida para a identificação de alimentos no questionário, primeiramente verifique as informações do rótulo (se houver), o tipo de embalagem e a lista de ingredientes (se houver). Se restarem dúvidas sobre os ingredientes, os recheios ou o “nome” anunciado pelo estabelecimento pergunte diretamente ao(a) entrevistado(a).

Caso você localize os alimentos abaixo sendo comercializados, deve identificá-los no questionário da seguinte forma:

Alimento	Orientação de verificação	Identificação
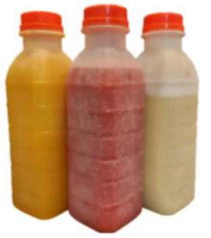<br>Fonte: Google imagens.<br>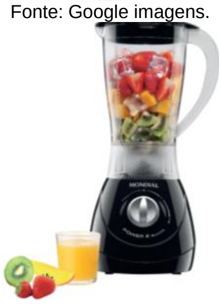<br>Fonte: Google imagens.	Note a ausência de rótulo e lista de ingredientes<br><br>Note que este tipo de embalagem, e ausência de rotulagem, é comum em alimentos de preparo artesanal<br><br>A bebida pode ser preparada e vendida imediatamente no copo<br><br>Verifique se a bebida é anunciada como suco ou refresco ou vitamina<br><br>Pergunte se a bebida foi feita com polpa de fruta/fruta (suco natural) ou se foi utilizado preparado em pó (refresco) ou se foi/será adicionado leite (leite batido com frutas).	MARCAR: 40. Suco natural da fruta
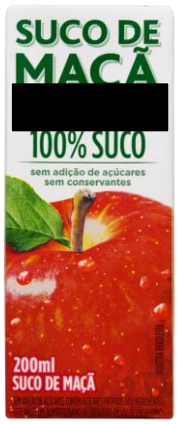<br><br>Fonte: Google imagens.	Note que na embalagem há “Suco de maçã” “100% suco” “sem adição de açúcares, sem conservantes”<br><br>Note que este tipo de embalagem (caixinha, garrafa, copo lacrado) é comum de alimentos industrializados<br><br>Peça a embalagem e note que na lista de ingredientes consta apenas o suco	MARCAR: 41. Suco 100% integral
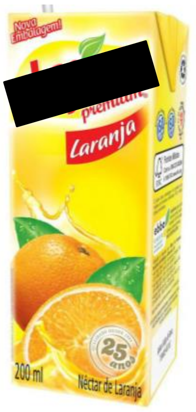<br><br>Fonte: Google imagens.	Note que na embalagem há “Néctar de laranja”<br><br>Note que este tipo de embalagem (caixinha, garrafa, copo lacrado) é comum de alimentos industrializados<br><br>Peça a embalagem e note que na lista de ingredientes há a adição de substâncias artificiais além do suco (ex. acidulante, aromatizante, conservante etc)	MARCAR: 42. Néctar
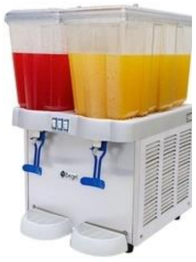<br>Fonte: Google imagens.	Note que este tipo de dispenser é comum na distribuição de refrescos<br><br>Verifique se a bebida é anunciada como suco ou refresco<br><br>Pergunte se a bebida foi feita com polpa de fruta/fruta (suco natural) ou se foi utilizado preparado em pó (refresco)	MARCAR: 43. Refresco

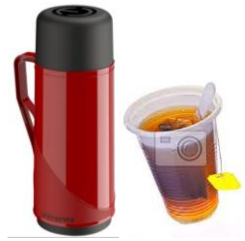 Fonte: Google imagens.	Note a ausência de rótulo e lista de ingredientes<br><br>Note que essa bebida normalmente fica armazenada em garrafa térmica e é vendida imediatamente no copo descartável ou xícara<br><br>Pergunte se a bebida foi feita com ervas/plantas ou se foi utilizado sachê	MARCAR: 45. Chá natural
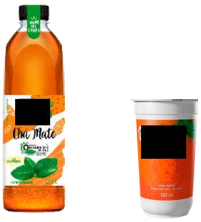 Fonte: Google imagens.	Note que este tipo de embalagem (garrafa, copo lacrado) é comum de alimentos industrializados<br><br>Peça a embalagem e note que na lista de ingredientes há a adição de substâncias artificiais além do chá (ex. acidulante, aromatizante, conservante etc)	MARCAR: 46. Chá pronto para beber
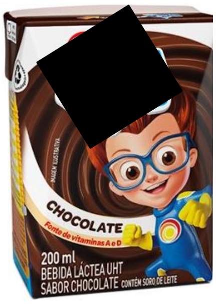 Fonte: Google imagens.	Note que na embalagem há “Bebida láctea”<br><br>Note que este tipo de embalagem (caixinha, garrafa, copo lacrado) é comum de alimentos industrializados<br><br>Peça a embalagem e note que na lista de ingredientes há a adição de substâncias artificiais além de leite ou soro do leite (ex. emulsificante, aromatizante, conservante etc)	MARCAR: 48. Bebida láctea e iogurte com sabor
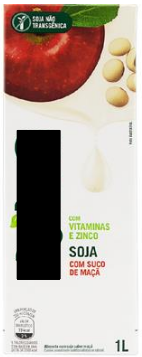 Fonte: Google imagens.	Note que na embalagem há “Soja com suco de maçã”<br><br>Note que este tipo de embalagem (caixinha, garrafa, copo lacrado) é comum de alimentos industrializados<br><br>Peça a embalagem e note que na lista de ingredientes há a presença de soja, sozinha ou adicionada a outros ingredientes (ex. acidulante, aromatizante, conservante etc)	MARCAR: 49. Bebida à base de soja
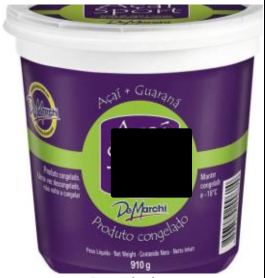 Fonte: Google imagens.	Note que na embalagem há “Açaí + Guaraná”<br><br>Pergunte se o açaí é adoçado ou de xarope<br><br>Peça a embalagem e note que na lista de ingredientes há a adição de substâncias adoçantes além do açaí (ex. açúcar, xarope, glicose, adoçante, aroma artificial de… etc)	MARCAR: 59. Açaí com açúcar ou xarope
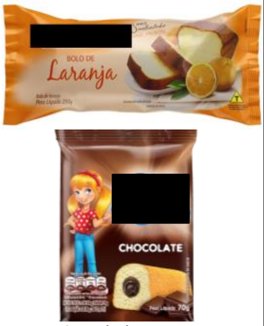 Fonte: Google imagens.	Note que este tipo de embalagem é comum de alimentos industrializados<br><br>Note na embalagem a provável presença do símbolo de transgênicos:<br><br>Peça a embalagem e note que na lista de ingredientes há a adição de uma série de substâncias artificiais (ex. emulsificante, aromatizante, conservante etc)	MARCAR: 63. Bolo ultraprocessado

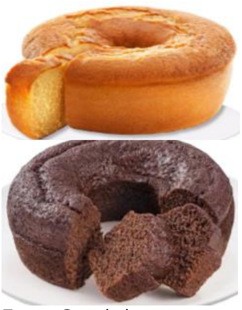<br>Fonte: Google imagens.	Note a ausência de rótulo e lista de ingredientes<br><br>O bolo pode ser vendido em fatias sem embalagem personalizada<br><br>Pergunte se o bolo foi feito com mistura pronta para bolo (bolo ultraprocessado) ou se foi feito com ingredientes comuns (bolo preparação culinária)	MARCAR: 64. Bolo preparação culinária
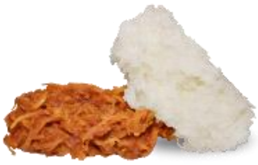<br>Fonte: Google imagens.	Note a ausência de rótulo e lista de ingredientes<br><br>Peça a embalagem (se houver) e note que na lista de ingredientes consta a fruta ou legume base além de açúcar e talvez ácido ascórbico	MARCAR: 65. Doce a base de fruta ou legume
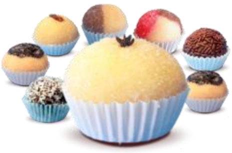<br>Fonte: Google imagens.	Note a ausência de rótulo e lista de ingredientes<br><br>Note que esses doces são aqueles parecidos com os servidos em festas de aniversários (no formato de bolinha) ou gelatina, geralmente feitos com leite condensado e/ou creme de leite, margarina e açúcar	MARCAR: 66. Doce com ingrediente ultraprocessado
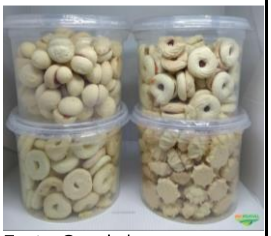<br>Fonte: Google imagens.	Peça a embalagem e note que na lista de ingredientes (se houver) há apenas ingredientes culinários de fácil identificação: manteiga, farinha, açúcar, ovos	MARCAR: 70. Biscoito preparação culinária
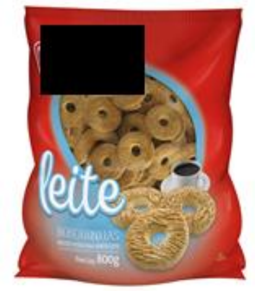<br>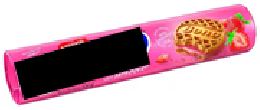<br>Fonte: Google imagens.	Geralmente se houver recheio e, na embalagem, uma ilustração associado ao sabor do recheio, considere como ultraprocessado<br><br>Note na embalagem a provável presença do símbolo de transgênicos: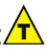<br><br>Peça a embalagem e note que na lista de ingredientes há a adição de uma série de substâncias artificiais (ex. emulsificante, aromatizante, conservante etc)	MARCAR: 71. Biscoito doce com ou sem recheio (ultraprocessado)
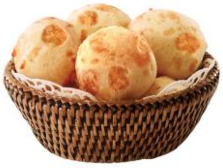<br>Fonte: Google imagens.	Os pãezinhos podem ser pode ser vendidos em unidades sem embalagem personalizada<br><br>Pergunte se o pão de queijo foi assado após retirado de embalagem de congelamento (ultraprocessado) ou se foi feito com ingredientes comuns (preparação culinária) trazido ao estabelecimento já assado<br><br>Peça a embalagem de congelamento (se houver) e note que na lista de ingredientes há a adição de uma série de substâncias	MARCAR: 73. Pão de queijo (ultraprocessado)

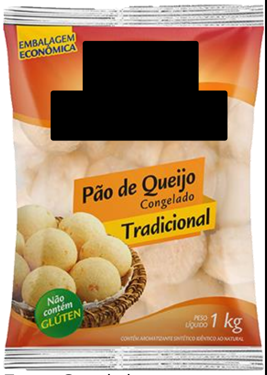 Fonte: Google imagens.	artificiais (ex. emulsificante, aromatizante, conservante etc)<br><br>Note se há alguma menção aos termos “artesanal” ou “caseiro” na embalagem (preparação culinária)	
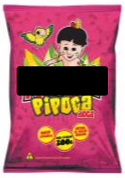 Fonte: Google imagens. 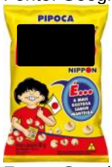 Fonte: Google imagens.	Note que este tipo de embalagem é comum de alimentos industrializados<br><br>Peça a embalagem e note que na lista de ingredientes consta a adição de uma série de substâncias artificiais além do milho(ex. ciclamato de sódio, sacarina sódica, gordura vegetal hidrogenada, margarina etc)	MARCAR: 86. Pipoca ultraprocessada doce/salgada
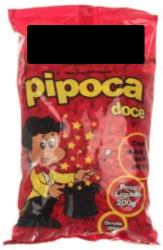 Fonte: Google imagens.	Note que este tipo de embalagem é comum de alimentos industrializados<br><br>Peça a embalagem e note que na lista de ingredientes consta apenas o milho de canjica ou canjicado, açúcar e/ou sal.	MARCAR: 87. Pipoca doce/salgada de pacote

Para a devida identificação de lanches você precisará diferenciar os recheios utilizados em tapiocas, pizzas, salgados assados, salgados fritos e sanduíche para que seja possível marcar como “COM ou SEM” recheios ultraprocessados. Preparamos o quadro abaixo para facilitar a identificação do tipo de recheio a ser marcado:

Recheios SEM ultraprocessados	Recheios COM ultraprocessados
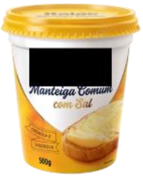<br><br>Manteiga<br>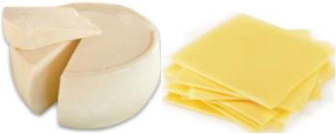<br>Queijos<br>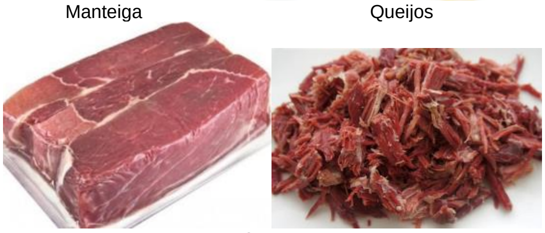<br>Carne seca<br>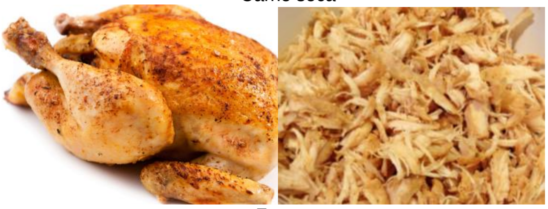<br>Frango<br>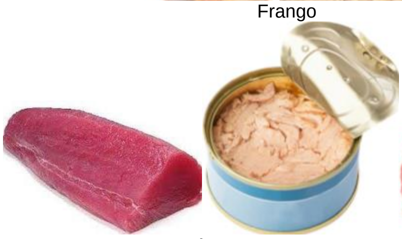<br>Atum<br>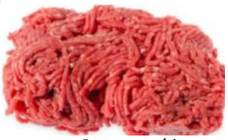<br>Carne moída<br>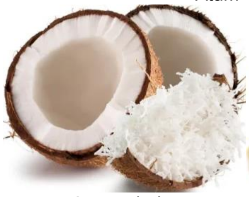<br>Coco ralado<br>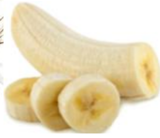<br>Banana<br>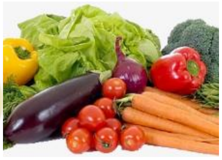<br>Vegetais<br>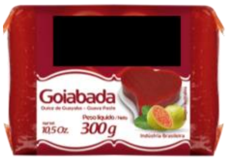<br><br>Goiabada<br>Fonte: Google imagens.	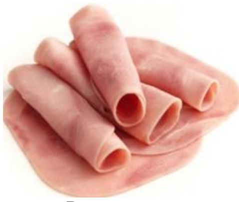<br>Presunto<br>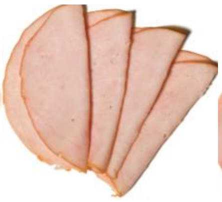<br>Peito de peru<br>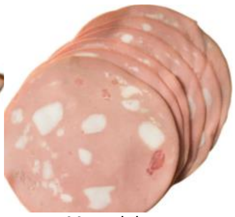<br>Mortadela<br>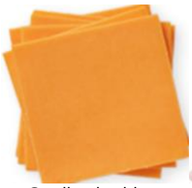<br>Queijo cheddar<br>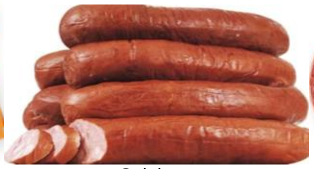<br>Calabresa<br>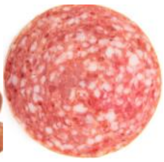<br>Salame<br>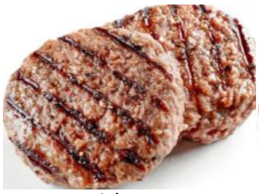<br>Hambúrguer<br>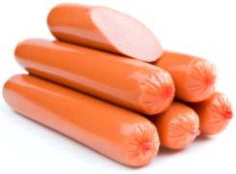<br>Salsicha<br>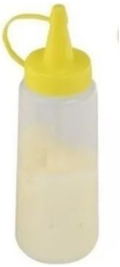<br>Maionese<br>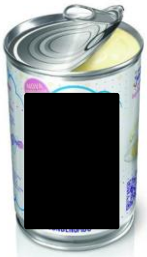<br><br>Leite condensado<br>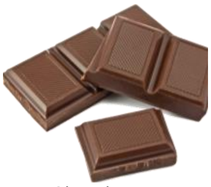<br>Chocolate<br>Fonte: Google imagens.

2.4 OBSERVAÇÃO DA VENDA INTERNA DE ALIMENTOS (Seção 1.2)

Nesta seção estão listados vários alimentos que você deve observar para identificar sua comercialização na cantina. Sugerimos que você siga seguinte ordem:

1º) Busque item por item da coluna de alimentos e assinale ("Sim" ou "Não") a coluna "Comercializado" quando identificá-lo à venda na cantina.

2º) Ao identificar o produto, anote o número de marcas ou sabores encontrados, NÃO PRECISA DETALHAR, <u>apenas conte o número de diferentes opções do mesmo tipo de alimento</u>.

Ex.

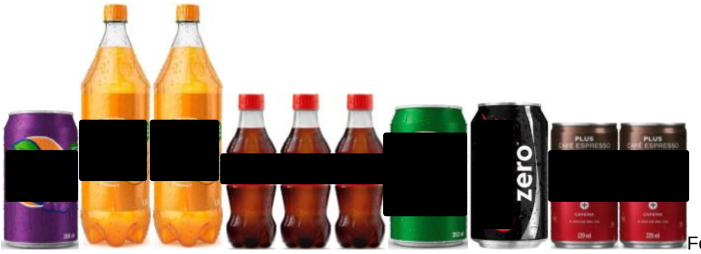

Fonte: Google imagens.

*Se há 2 marcas de refrigerante e cada uma com 3 sabores diferentes, anota-se o número **6** na coluna "Variedade".*

3º) Conhecendo a variedade, anote o tamanho (em g, mg, L, mL) do item de menor preço (o mais barato) e respectivo o menor preço (R$) disponível.

<u>Atenção: para preparações culinárias levar em conta a unidade como tamanho.</u>

4º) Por fim, identifique se há combos (venda associada a outros produtos diferentes) ou promoções (compra unitária ou duplicada do mesmo item com vantagem econômica ou acréscimo item "gratuito") anunciados na cantina (no cardápio, em banner/cartaz, folder, display etc.), em seguida assinale o(s) respectivo(s) alimento(s) anunciado(s) no combo e/ou promoção.

Combo	Promoção
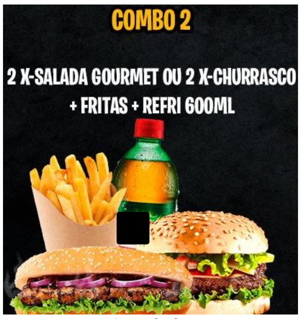<br><br>Fonte: Google imagens.	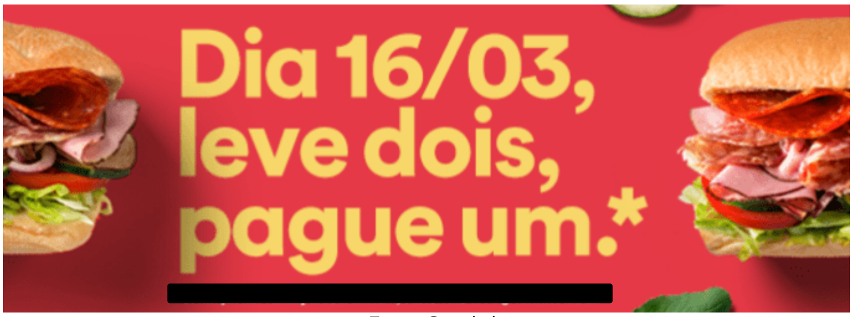<br><br>Fonte: Google imagens.

Caso identifique algum produto comercializado no comércio ambulante que não esteja presente na lista, use a opção "outros" e descreva qual(is) é(são) o(s) produto(s).

IMPORTANTE: SE for autorizado pelo(a) entrevistado(a), fotografe o cardápio (se houver), o espaço geral da cantina (sem captar a identidade da escola ou indivíduos) e os materiais publicitários (se houver).

Cardápio (frente e verso)	Materiais publicitários
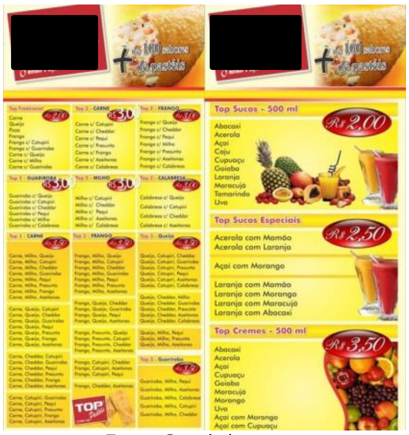<br><br>Fonte: Google imagens.	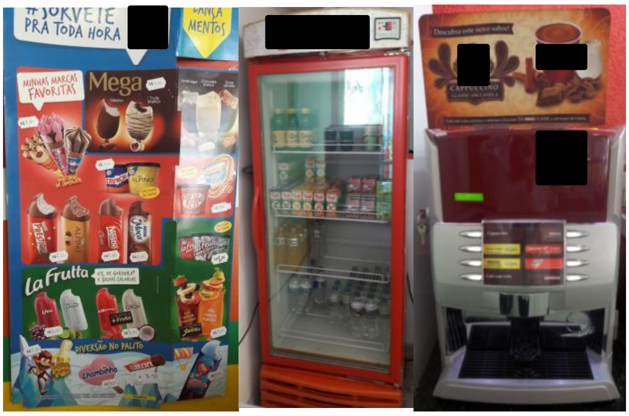<br><br>Fonte: Autoras do manual

COMO FOTOGRAFAR A CANTINA: utilize o celular/câmera na posição horizontal e distancie-se pelo menos 1m para captar a estrutura geral da cantina.

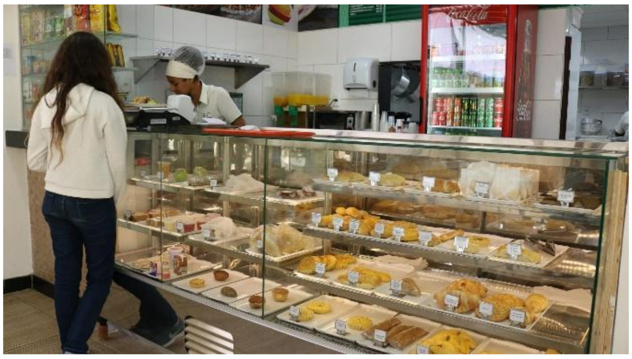

Fonte: Google imagens.

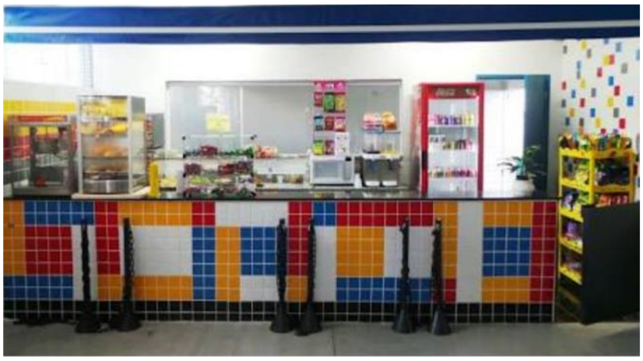

Fonte: Google imagens

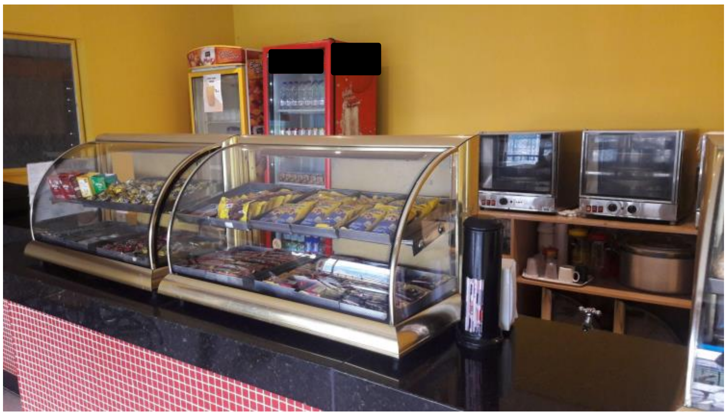

Fonte: Autoras do manual

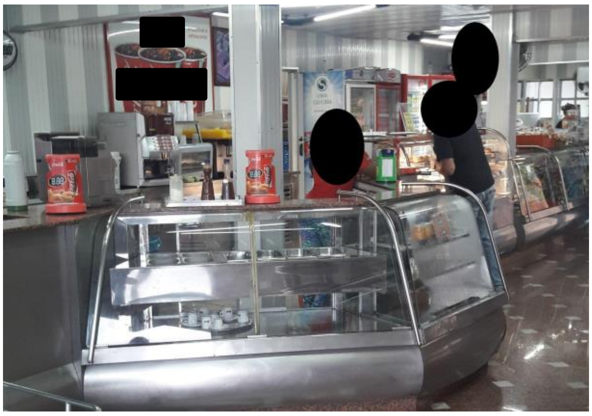

Fonte: Autoras do manual

2.5 OBSERVAÇÃO DA PUBLICIDADE INTERNA DE ALIMENTOS (Seção 1.3)

Nesta seção estão listados os mesmos alimentos da seção anterior. Sugerimos que você siga seguinte ordem:

1º) Localize as formas de apresentação de publicidade existentes na cantina (exemplos no quadro abaixo).

2º) Identifique quais alimentos estão sendo anunciados nas determinadas formas de apresentação listadas no questionário, em seguida assinale (“Sim” ou “Não”) os produtos propagandeados em cada forma de apresentação.

Formas de apresentação

Banner/cartaz do fornecedor

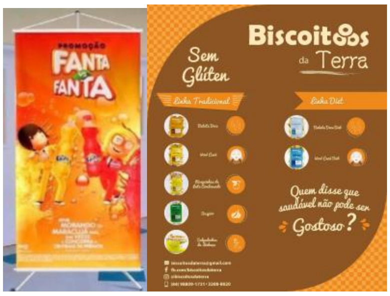

Fonte: Google imagens.

Banner/cartaz do estabelecimento

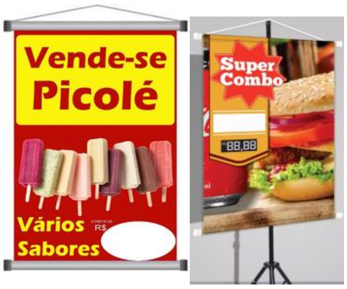

Fonte: Google imagens.

Vestimenta

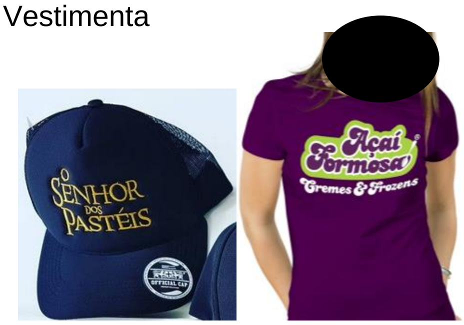

Fonte: Google imagens.

Cardápio

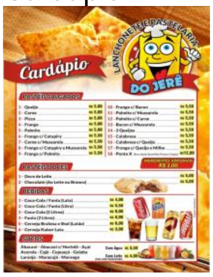

Fonte: Google imagens.

Embalagem

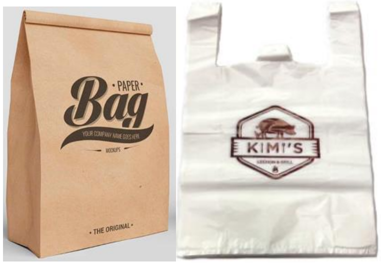

Fonte: Google imagens.

Painel/televisão

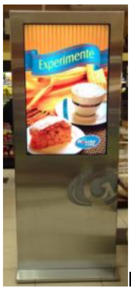

Fonte: Google imagens.

Folder

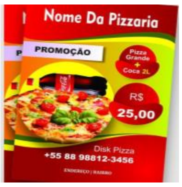

Fonte: Google imagens.

Display

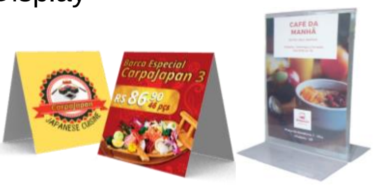

Fonte: Google imagens.

Brindes

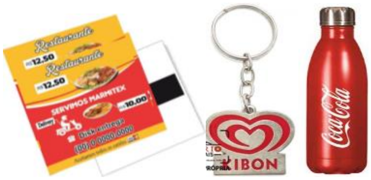

Fonte: Google imagens.

Mobiliário

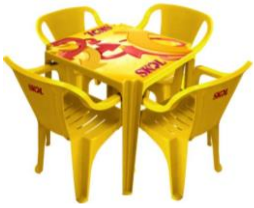

Fonte: Google imagens.

Utensílios

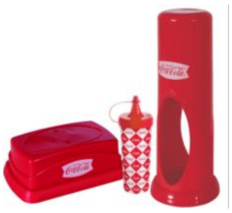

Fonte: Google imagens.

Equipamentos

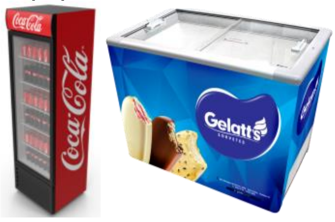

Fonte: Google imagens.

Aplicativo da escola/cantina

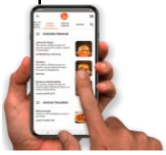

Fonte: Google imagens.

3. INSTRUMENTO PARA AVALIAÇÃO DO ENTORNO DAS ESCOLAS – SEÇÃO 2

3.1 ENTREVISTA COM OS AMBULANTES

Questão 138: Nome da escola de proximidade.

Preencha o nome completo da escola a qual o comércio ambulante está próximo.

Questão 139: Data da aplicação.

Questão 140: Nome do(a) vendedor(a) entrevistado(a).

Preencher o nome completo do(a) entrevistado(a).

Questão 141: Idade.

Pergunte ao(a) vendedor(a) "Poderia me informar, por favor, a sua idade?" e preencha no espaço determinado.

Questão 142: Gênero.

Pergunte ao(a) vendedor(a) "Você se declara do gênero masculino, feminino, outro ou prefere não declarar?" e assinale de acordo com a resposta dele(a). A partir daqui, se refira a ele(a) conforme o gênero informado.

Questão 143: Escolaridade.

Pergunte ao(a) vendedor(a) "E qual a sua escolaridade?" ou "Até que série o(a) senhor(a) estudou; concluiu na escola?" e assinale de acordo com a resposta dele(a). Lembrando que uma pessoa analfabeta é aquela que não pode participar de todas as atividades nas quais a alfabetização é requerida. É considerado "Completo" quando o indivíduo cumpriu todos os requisitos escolares e possui certificado ou diploma para o determinado grau. **Marcar apenas uma opção.**

Questão 144: Raça/cor/etnia.

Pergunte ao(a) vendedor(a) "Como o(a) senhor(a) autodeclara a sua raça/cor/etnia?" e assinale de acordo com a resposta dele(a). **Marcar apenas uma opção.**

Questão 145: Localização do vendedor(a).

Observe e assinale em qual local o estabelecimento se encontra.

Localização	Caracterização	
(1) Porta da escola	para estabelecimentos que estão no passeio/calçada que circunda a escola e/ou localizados imediatamente em frente ao portão de saída ou entrada da escola.	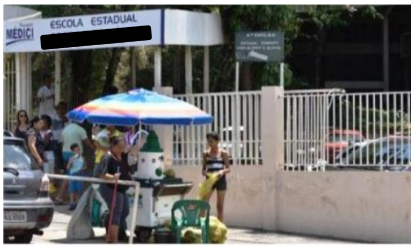 Fonte: Google imagens.
(2) No quarteirão	para estabelecimentos que estão localizados no passeio/calçada do outro lado da rua em que a escola está situada, mas posicionados voltados à fachada da escola.	
(3) Outros	aqueles que estão em locais que não se enquadram em nenhum dos dois anteriores.	Descreva a localização no espaço determinado.<br><br>Ex. Na calçada do outro lado da rua, voltado à lateral da escola.

Questão 146: Tipo de estrutura.

Observe e assinale (sim ou não) o tipo de estrutura utilizada para a venda.

Estrutura	Caracterização	Exemplos	
146.1 Carrinho	aqueles meios de venda que possuem rodas para locomoção, porém não são motorizados, podem possuir cobertura ou não.	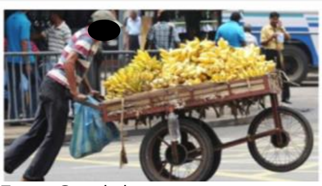 Fonte: Google imagens.	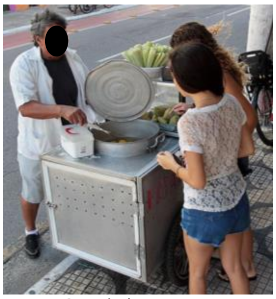 Fonte: Google imagens.
146.2 Carro particular	aqueles meios de comércio que ocorrem no interior ou com o suporte imediato de um carro de passeio comum motorizado sem identificação de logomarcas nem modificações específicas para venda de alimentos.	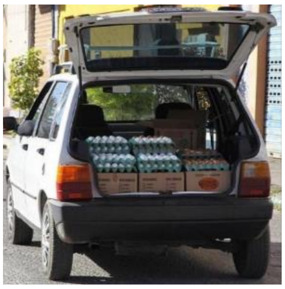 Fonte: Google imagens.	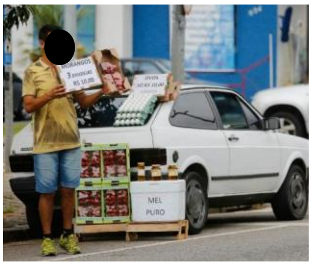 Fonte: Google imagens.
146.3 Barraca	aqueles meios de venda que são cobertos com estrutura de lona ou outro material e não possuem rodas e/ou motores.	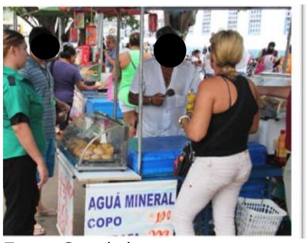 Fonte: Google imagens.	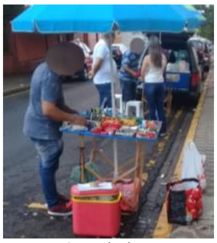 Fonte: Google imagens.

146.4 Food Truck	aqueles meios de comércio que possuem rodas e são motorizados, geralmente são automóveis modificados para venda de alimentos. Podem possuir cobertura metálica e estrutura mais elaborada.	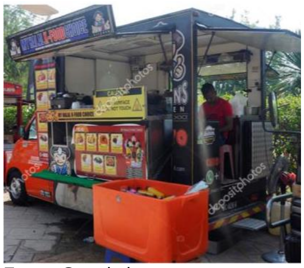 Fonte: Google imagens.	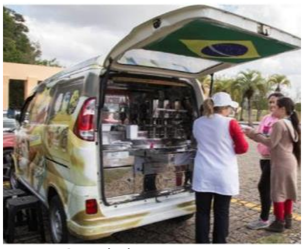 Fonte: Google imagens.
146.5 Banca	aqueles meios de comércio que não possuem cobertura, rodas ou motor. Meio de vendas simples, de fácil desmonte e transporte.	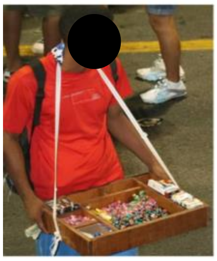 Fonte: Google imagens.	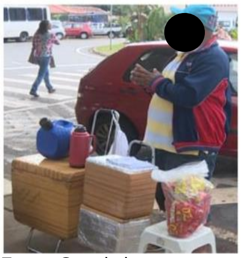 Fonte: Google imagens.
146.6 Isopor	aqueles meios de comércio que se resumem ao comércio do conteúdo acondicionado em caixa térmica isopor ou polietileno. Podendo haver suporte com rodas ou não.	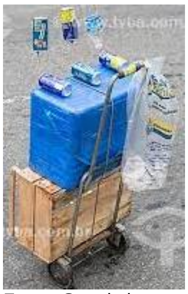 Fonte: Google imagens.	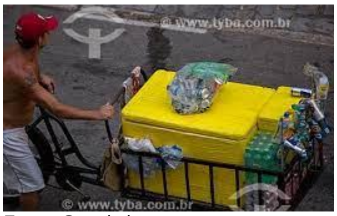 Fonte: Google imagens.
146.7 Outros	aqueles meios que não se enquadram em nenhum dos outros meios de venda anteriores.	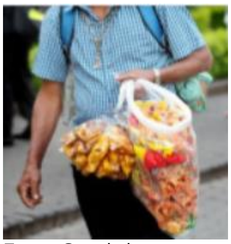 Fonte: Google imagens.	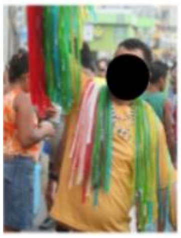 Fonte: Google imagens.

Em caso de dúvidas, não assinale! Tire uma foto (quando autorizado pelo(a) entrevistado(a)) e apresente aos coordenadores de campo.

Questão 147: Principal fonte de renda.

Pergunte ao(a) vendedor(a) “Este trabalho é a sua principal fonte de renda?” Assinale de acordo com a resposta dele(a) “Sim” ou “Não”. Caso ele(a) responda “Não”, peça para que ele(a) informe a principal atividade de trabalho e anote no espaço determinado.

Questão 148: Tempo de venda de alimentos como ambulante.

Pergunte ao(a) vendedor(a) “*Há quanto tempo vende alimentos como ambulante*?”. Assinale a opção de período que corresponde à resposta dele(a).

Questão 149: Outro local de funcionamento.

Pergunte ao(a) vendedor(a) “O(A) senhor(a) vende alimentos ou bebidas em outro local, além daqui?”. Assinale “Sim” ou “Não” de acordo com a resposta obtida.

Questão 150: Resistência à sua permanência.

Pergunte ao(a) vendedor(a) "*Alguém já tentou impedir ou proibir a sua atividade neste local*?". Assinale de acordo com a resposta dele(a) "Sim" ou "Não". Caso ele(a) responda "Sim" pergunte "Por parte de quem? (ex. População, poder público, polícia, prefeitura etc.)" e anote no espaço determinado.

Questão 151: Interferência da gestão escolar.

Pergunte ao(a) vendedor(a) "*A gestão da escola já tentou interferir no que o(a) senhor(a) vende?"*. Assinale de acordo com a resposta dele(a) "Sim" ou "Não". Caso ele(a) responda "Sim" pergunte "Sobre quais itens/alimentos?" e anote no espaço determinado.

Questão 152: Produção dos alimentos comercializados.

Pergunte ao(a) vendedor(a) "*Onde são produzidos os alimentos comercializados pelo(a) senhor(a)?* Leia as opções do questionário para ele(a) e assinale "Sim" ou "Não" para cada opção de local no questionário, de acordo com a resposta obtida. E diga "Se houver outro local de produção além dos citados aqui, também pode me dizer". Assinale e preencha a opção "Outros" se ele(a) informar outro local de produção além dos indicados para assinalar.

Questão 153: Horário de trabalho.

Pergunte ao vendedor(a) "Qual o período em que seu ponto de comércio está funcionando neste local?" (Note que é especificamente no local de atuação em que ele se encontra naquele momento!). Assinale de acordo com a resposta obtida. **Marcar apenas uma opção.**

Questão 154: Dias de trabalho.

Pergunte ao vendedor(a) "Quais os dias em que o seu ponto de comércio está funcionando neste local?" (Note que é especificamente no local de atuação em que ele se encontra naquele momento!). Leia as opções do questionário para ele(a) e assinale "Sim" ou "Não" para cada opção de dia no questionário, de acordo com a resposta obtida.

Questão 155: Opção pelo comércio ambulante.

Pergunte ao(a) vendedor(a) "Qual o principal motivo que o levou a estar nesta profissão?" Leia as opções do questionário para ele(a), e diga "Se houver outro motivo além dos citados aqui, também pode me dizer". Assinale a opção que corresponde ao que o(a) vendedor(a) informou. Assinale e preencha a opção "Outros" se ele(a) informar outro motivo além dos indicados para assinalar. **Marcar apenas uma opção.**

Questão 156: Cadastro ou licença.

Pergunte ao(a) vendedor(a) "Você possui algum tipo de cadastro ou licença junto a prefeitura?". Assinale "Sim" ou "Não" de acordo com a resposta obtida. Caso ele(a) responda "Sim" pergunte "Qual?" e digite no espaço determinado.

Questão 157: Material publicitário.

Pergunte ao(a) vendedor(a) “O(A) senhor(a) já recebeu algum material de incentivo/patrocínio/apoio dos fornecedores ou marcas dos produtos que comercializa? Como por exemplo freezer, mesa, porta guardanapo, isopor etc.”. Assinale “Sim” ou “Não” de acordo com a resposta obtida. Ainda que a resposta seja “Não”, observe e assinale a presença de materiais descritos na questão 159.

Questão 158: Material publicitário recebido.

Apenas SE você houver identificado materiais listados na questão 159, digite no local destinado quais empresas patrocinadoras foram identificadas.

Questão 159: Material publicitário recebido.

Observe as opções de materiais listados no questionário e assinale “Sim” quando identificar sua presença. Quando não encontrar alguma das opções, pergunte ao(a) entrevistado(a) se há a presença do determinado item não encontrado e assinale “Sim ou Não” conforme o(a) vendedor(a) informar. Você pode perguntar.

3.2 OBSERVAÇÃO DOS ALIMENTOS COMERCIALIZADOS NO ENTORNO DAS ESCOLAS (Seção 2.2)

Nesta seção estão listados vários alimentos que você deve observar para identificar sua comercialização no comércio ambulante. Sugerimos que você siga seguinte ordem:

1º) Busque item por item da coluna de alimentos e assinale (“Sim” ou “Não”) a coluna “Comercializado” quando identificá-lo no estabelecimento.

2º) Ao identificar o produto, anote o número de marcas ou sabores encontrados, NÃO PRECISA DETALHAR, apenas conte o número de diferentes opções do mesmo tipo de alimento.

Ex.

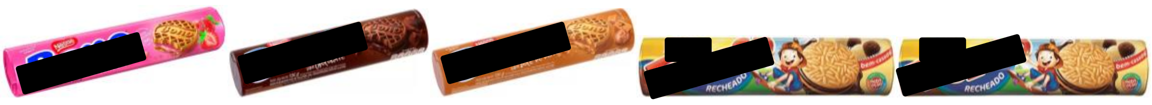

Fonte: Google imagens.

Se há 2 marcas de biscoito, onde 1 tem 3 sabores diferentes e a outra tem 1 sabor, anota-se o número ***4*** *na coluna “Variedade”.*

3º) Conhecendo a variedade, anote o tamanho (em g, mg, L, mL) do item de menor preço (o mais barato) e respectivo o menor preço (R$) disponível.

4º) Por fim, identifique se há combos (venda associada a outros produtos diferentes) ou promoções (compra unitária ou duplicada do mesmo item com vantagem econômica ou acréscimo de item “gratuito”) anunciados no comércio ambulante (no cardápio, em banner/cartaz, folder, display etc.), em seguida assinale o(s) respectivo(s) alimento(s) anunciado(s) no combo e/ou promoção.

<table>
<tr><th>Combo</th><th>Promoção</th></tr>
<tr><td>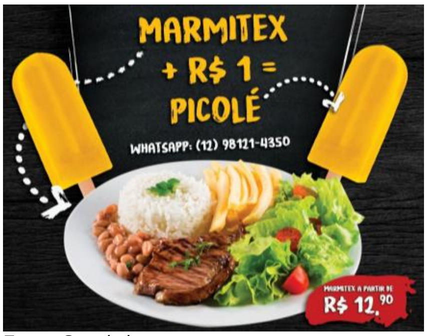

Fonte: Google imagens.</td><td>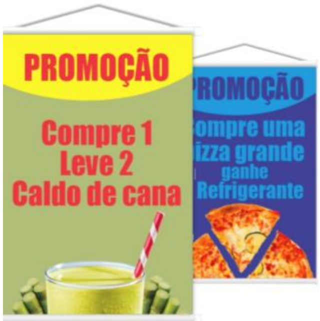

Fonte: Google imagens.</td></tr>
</table>

Caso identifique algum produto comercializado no comércio ambulante que não esteja presente na lista, use a opção “outros” e descreva qual(is) é(são) o(s) produto(s).

IMPORTANTE: SE for autorizado pelo(a) vendedor(a), fotografe o cardápio (se houver), o espaço geral do comércio (sem captar a identidade da escola ou indivíduos) e os materiais publicitários (se houver).

<table>
<tr><th>Cardápio (frente e verso)</th><th>Materiais publicitários</th></tr>
<tr><td>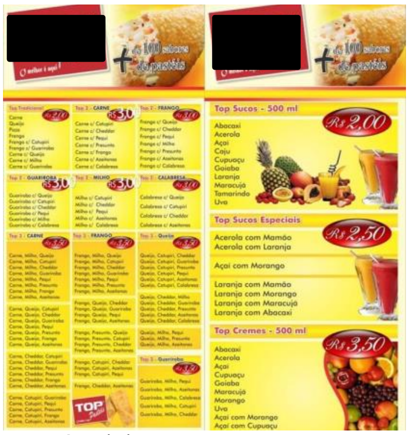

Fonte: Google imagens.</td><td>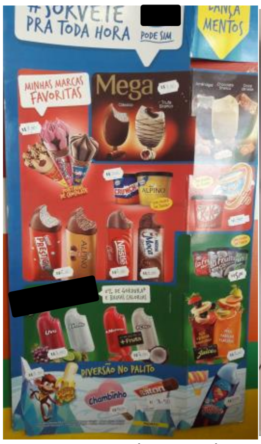

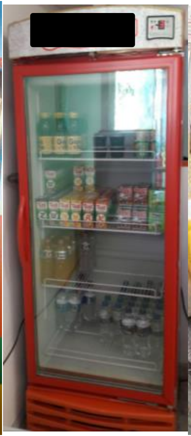
Fonte: Autoras do manual.
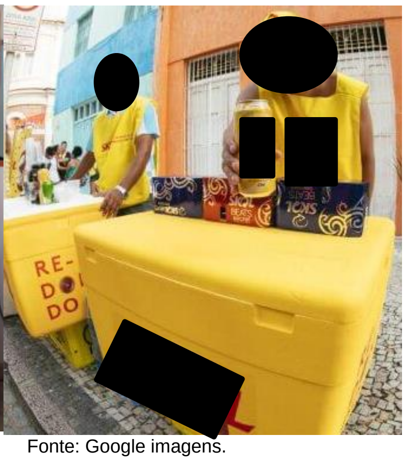
Fonte: Google imagens.</td></tr>
</table>

COMO FOTOGRAFAR: utilize o celular/câmera na posição horizontal e distancie-se pelo menos 1m para captar a estrutura geral do comércio ambulante.

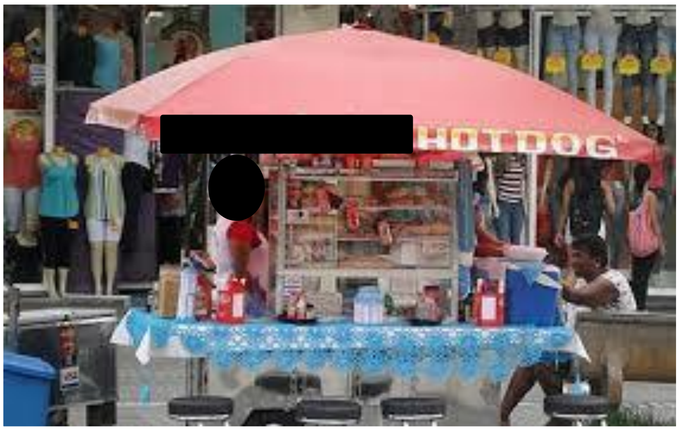

Fonte: Google imagens.

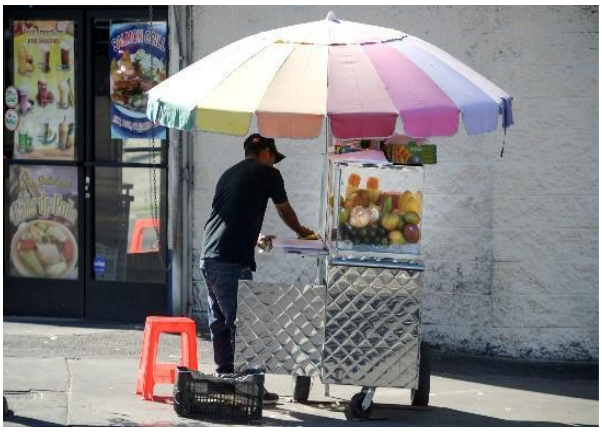

Fonte: Google imagens.

3.3 OBSERVAÇÃO DA PUBLICIDADE DE ALIMENTOS NO ENTORNO DAS ESCOLAS (Seção 2.3)

Nesta seção estão listados os mesmos alimentos da seção anterior. *Lembre-se de identificar o nome da escola a qual o comércio ambulante está próximo.* Sugerimos que você siga seguinte ordem:

1º) Localize as formas de apresentação de publicidade existentes no estabelecimento (exemplos no quadro abaixo).

2º) Identifique quais alimentos estão sendo anunciados nas determinadas formas de apresentação listadas no questionário, em seguida assinale (“Sim” ou “Não”) os produtos propagandeados em cada forma de apresentação.

Formas de apresentação		
Banner/cartaz do fornecedor<br>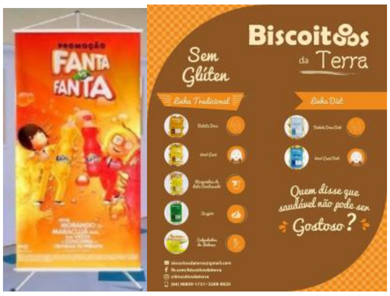<br><br>Fonte: Google imagens.	Banner/cartaz do estabelecimento<br>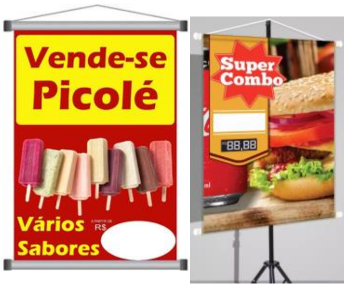<br><br>Fonte: Google imagens.	Vestimenta<br>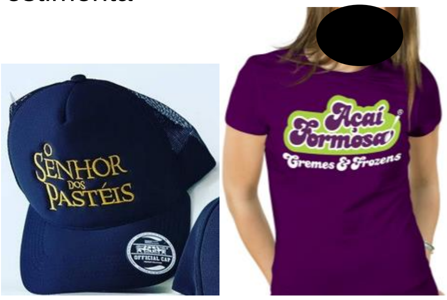<br><br>Fonte: Google imagens.
Cardápio<br>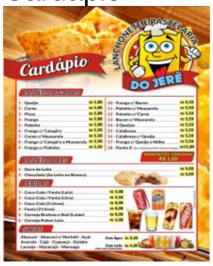<br><br>Fonte: Google imagens.	Embalagem<br>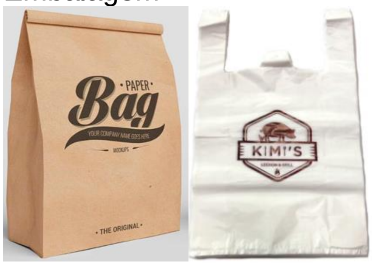<br><br>Fonte: Google imagens.	Painel/televisão<br>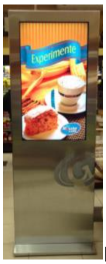<br>Fonte: Google imagens.
Folder<br>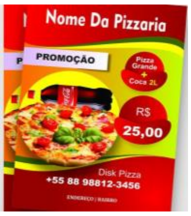<br><br>Fonte: Google imagens.	Display<br>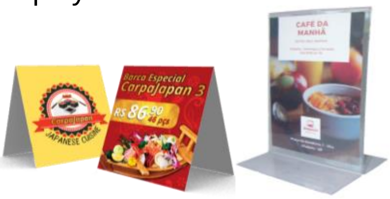<br>Fonte: Google imagens.	Brindes<br>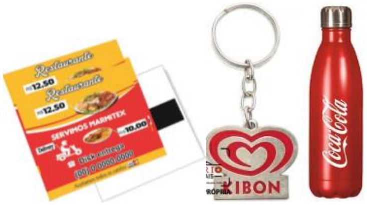<br><br>Fonte: Google imagens.
Mobiliário<br>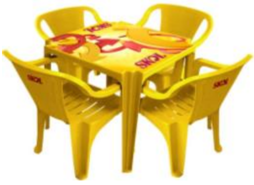<br>Fonte: Google imagens.	Utensílios<br>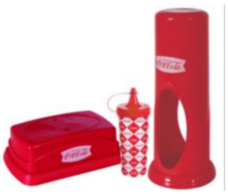<br>Fonte: Google imagens.	Equipamentos<br>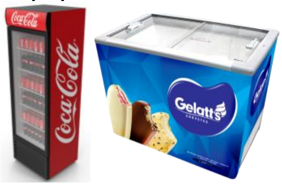<br><br>Fonte: Google imagens.
Aplicativo da escola/cantina<br>**NA**		
