# Supplementary material for: Development, content validity, and reliability of an instrument to assess the commercialization of food and beverages in school canteens in Brazil
Source: Rev Bras Epidemiol. 2026 Apr 3;29:e260015. doi: 10.1590/1980-549720260015 (PMC13053033; doi:10.1590/1980-549720260015)
Supplement: Translation

# Translation: Elaboração, validade de conteúdo e confiabilidade de instrumento de avaliação da comercialização de alimentos e bebidas em cantinas escolares do Brasil

**DOI:** 10.1590/1980-549720260015.2

**Authors:** Luiza Delazari Borges, Letícia Ferreira Tavares, Ariene Silva do Carmo, Letícia de Oliveira Cardoso, Raquel Canuto, Paulo César Pereira de Castro, Sabrina Gomes Ferreira Clark, Luísa Arantes Vilela, Larissa Loures Mendes

**Affiliations:** I Universidade Federal de Minas Gerais - Belo Horizonte (MG), Brasil.; II Universidade Federal do Rio de Janeiro - Rio de Janeiro (RJ), Brasil.; III Universidade Federal de Ouro Preto - Ouro Preto (MG), Brasil.; IV Fundação Oswaldo Cruz, Escola Nacional de Saúde Pública Sergio Arouca - Rio de Janeiro (RJ), Brasil.; V Universidade Federal do Rio Grande do Sul - Porto Alegre (RS), Brasil.; VI Universidade Federal de Pernambuco - Recife (PE), Brasil.

**Keywords:** Alimentação escolar, Ambiente, Confiabilidade e validade, Espaço social alimentar

## Abstract

**Objetivos:**

Elaborar, verificar a validade de conteúdo e avaliar a confiabilidade de um instrumento de avaliação da comercialização de alimentos e bebidas em cantinas escolares.

**Métodos:**

A versão preliminar do instrumento foi construída após revisão de literatura, identificação das dimensões do ambiente alimentar, elaboração dos itens e estruturação do instrumento, dividido em três seções, quais sejam: identificação e caracterização da cantina, alimentos comercializados e publicidade dos alimentos comercializados. O instrumento foi avaliado por especialistas e foi calculado o índice de validade de conteúdo (IVC) para cada item do instrumento (satisfatório >0,80). A confiabilidade foi examinada em uma amostra de cantinas por meio do teste interobservador e intraobservador. Foram calculados valores de *kappa*, PABAK e coeficiente de correlação intraclasse.

**Resultados:**

O instrumento contemplou 945 itens. A média de pontuação do IVC do instrumento, considerando os aspectos avaliados (relevância, clareza da escrita dos itens e das suas opções de respostas) foi de 0,95. As sugestões dos especialistas foram avaliadas e os itens foram incluídos, retirados ou alterados quando pertinente. Com relação à confiabilidade, 95,2% do total dos itens analisados apresentou concordância excelente, muito boa ou boa (≥0,6).

**Conclusão:**

O instrumento apresentou validade de conteúdo adequada e confiabilidade satisfatória no contexto das escolas estudadas para mensurar o ambiente alimentar de cantinas escolares. Dessa forma, contribui para o avanço da aferição da comercialização de alimentos em cantinas, um importante aspecto do ambiente alimentar escolar.

## INTRODUÇÃO

Os ambientes nos quais as pessoas estão inseridas influenciam de diversas maneiras as escolhas alimentares e o consumo de alimentos, por meio de um conjunto de elementos que englobam aspectos físicos, econômicos, políticos e socioculturais dos ambientes^1^ ^,^ ^2^ ^,^ ^3^. Assim, o ambiente alimentar escolar é definido como os espaços, a infraestrutura e as condições, dentro e ao redor das escolas, em que os alimentos estão disponíveis, podendo ser obtidos, comprados ou consumidos: cantinas, lanchonetes, máquinas de autosserviço e vendedores ambulantes, bem como qualquer instalação em que haja venda de alimentos. Inclui também informações, promoção (*marketing*, propaganda, marcas, rótulos, embalagens) e precificação dos alimentos^4^.

Nesse contexto, as cantinas escolares destacam-se como espaços dentro das escolas destinados à comercialização de alimentos e bebidas para a comunidade escolar^5^. Elas influenciam as escolhas alimentares, sendo acessíveis a 31,4% dos alunos de escolas públicas e 88% dos estudantes de instituições privadas no Brasil, segundo a Pesquisa Nacional de Saúde do Escolar (PeNSE) de 2019^6^.

A comercialização de alimentos não saudáveis nas escolas está associada ao maior consumo desses alimentos entre os alunos^7^ ^,^ ^8^ ^,^ ^9^. No Brasil, foi verificada associação entre a compra regular de lanches nas cantinas a uma maior chance de consumo de bebidas açucaradas pelos adolescentes^10^ e à alta prevalência da comercialização de alimentos ultraprocessados em escolas privadas^11^.

Diante desse cenário, destaca-se a importância de instrumentos com boas propriedades psicométricas para avaliar a comercialização de alimentos em cantinas escolares. Ainda são escassos os estudos que validam ou verificam a confiabilidade desses instrumentos. Pesquisas realizadas na Austrália^12^ ^,^ ^13^ ^,^ ^14^ ^,^ ^15^, Nova Zelândia^16^, África do Sul^17^ ^,^ ^18^ e Noruega^19^ avaliaram a validade e/ou confiabilidade de instrumentos aplicados a diferentes aspectos do ambiente escolar. No Brasil, não há instrumentos validados para medir de forma precisa e confiável a comercialização de alimentos em cantinas escolares, dificultando a comparação entre estudos^20^ e limitando o avanço do conhecimento científico^21^.

Tendo em vista a lacuna e o impacto do ambiente alimentar escolar no consumo alimentar de crianças e adolescentes, o presente estudo teve como objetivo elaborar, verificar a validade de conteúdo e avaliar a confiabilidade de um instrumento de avaliação da comercialização de alimentos e bebidas nesses espaços.

## MÉTODOS

### Elaboração do instrumento

O instrumento foi elaborado no contexto do estudo Comercialização de Alimentos em Escolas Brasileiras (Caeb), cujo objetivo principal foi avaliar a comercialização de alimentos e bebidas em escolas privadas das capitais brasileiras^22^.

A primeira etapa da elaboração do instrumento foi a revisão de instrumentos existentes que avaliam o ambiente alimentar escolar e contêm dimensões ou itens aplicáveis para o contexto brasileiro. A revisão foi conduzida por dois pesquisadores nas bases Sistema Online de Busca e Análise de Literatura Médica (MEDLINE, via United States National Library of Medicine - PubMed) e Scientific Electronic Library Online (SciELO) em 2022. As versões preliminares do instrumento foram elaboradas com base na identificação das dimensões, formulação dos itens e estruturação do instrumento.

Entre as dimensões identificadas estão a disponibilidade (que inclui a variedade de alimentos disponíveis), a acessibilidade financeira (preço), a comodidade/conveniência (formas de pagamento, horário de funcionamento)^23^, a informação nutricional^1^, a promoção de alimentos (publicidade)^1^ ^,^ ^3^ ^,^ ^24^ ^,^ ^25^ ^,^ ^26^, a infraestrutura para a produção de refeições, a governança^27^ e a política^3^. Os instrumentos que serviram de base para a elaboração do instrumento foram: “Instrumento de avaliação do ambiente alimentar: ESAO-restaurantes”^28^; “Instrumento de auditoria do ambiente alimentar baseado na nova classificação de alimentos do Guia Alimentar (NOVA) - AUDIT-NOVA”^29^; “Instrumento de avaliação do ambiente alimentar universitário”^30^; o instrumento da PeNSE^6^ e o Instrumento do projeto “Segurança alimentar em cantinas escolares dos municípios do território da cidadania do noroeste colonial/RS^31^. Os itens referentes a alimentos foram elaborados com base nas recomendações do Guia Alimentar para a População Brasileira^32^ e no “Instrumento de avaliação do ambiente alimentar universitário”^30^.

O instrumento foi organizado em duas seções: Seção 1, destinada à identificação e caracterização da cantina; e Seção 2, voltada para os alimentos comercializados. Após a elaboração das versões preliminares, passou por avaliação de especialistas. Esse processo compõe a fase prototípica, essencial para o refinamento dos esboços iniciais e a criação de um instrumento adequado à mensuração proposta^21^.

O instrumento foi desenvolvido para uso por pesquisadores, sociedade civil, governo e demais interessados, visando avaliar a comercialização de alimentos em cantinas escolares com foco na alimentação adequada e saudável. Para isso, é fundamental compreender o funcionamento da cantina, cuja organização e operação influenciam a disponibilidade de alimentos.

O instrumento não gera um escore único nem uma classificação geral, pois cada dimensão pode ser analisada separadamente conforme o aspecto avaliado. No entanto, com base nos resultados da Seção 2, referentes à disponibilidade de alimentos, tem sido calculado o índice de saudabilidade^33^. Esse índice é obtido pela soma da disponibilidade de alimentos *in natura*, minimamente processados, processados e preparações culinárias baseadas nesses alimentos (AIMPP) e da não disponibilidade de alimentos ultraprocessados e preparações culinárias baseadas nesses alimentos (AUPP). O valor final varia de 0 a 100, indicando maior adequação quanto mais próximo de 100^33^.

### Avaliação por especialistas

Recomenda-se que de cinco a 20 especialistas avaliem o instrumento^34^ ^,^ ^35^. Neste estudo, 12 pesquisadores da área da nutrição e vinculados a diferentes instituições de pesquisa, com especialidade em epidemiologia, saúde coletiva, ambientes alimentares, alimentação coletiva ou gastronomia, foram convidados a participar da avaliação. Todos possuíam experiência nos temas do estudo e/ou na construção de instrumentos.

A versão preliminar foi analisada em um único momento quanto à relevância dos itens para os objetivos do estudo, clareza da redação e ausência de ambiguidade, com base em escala Likert de quatro pontos, em que: 1=item não equivalente; 2=item necessita de grande revisão para ser avaliada a equivalência; 3=item equivalente, necessita de pequenas alterações; e 4=item absolutamente equivalente^36^. Os avaliadores registraram comentários/sugestões. Essa etapa, de abordagem qualitativa, integra a fase prototípica da elaboração do instrumento^21^.

### Pré-teste

O pré-teste foi realizado em uma escola com características semelhantes às escolas da amostra do estudo Caeb, por meio do instrumento impresso, e foi realizado por uma pesquisadora treinada.

### Confiabilidade do instrumento

A confiabilidade do instrumento foi avaliada em uma amostra de conveniência composta de escolas públicas e privadas de uma capital e de um município da sua região metropolitana. A estimativa da amostra de cantinas avaliadas (n=13) foi realizada com base nos critérios propostos por Hulley et al.^37^ para o cálculo do coeficiente de correlação. Considerou-se uma correlação média de 0,70, a qual é classificada como boa concordância segundo os critérios estabelecidos por Byrt^38^. Adotou-se nível de significância de 5% e poder de teste de 80%. Foram convidadas a participarem do estudo 28 cantinas.

A coleta ocorreu entre setembro e dezembro de 2023 por meio do aplicativo Collect Box^39^ e foi aplicada por duas pesquisadoras treinadas e com experiência em ambiente alimentar escolar. As cantinas foram avaliadas três vezes^40^ ^,^ ^41^: pelas duas pesquisadoras no mesmo dia, com até 30 minutos de intervalo (teste interobservador), e por uma delas novamente após, em média, 21 dias (teste intraobservador).

A coleta combinou diferentes estratégias: algumas informações foram obtidas por meio de entrevista com gestores ou funcionários da cantina, enquanto outras decorreram de observação direta (auditoria). Para assegurar a padronização dos procedimentos, foi utilizado o manual de coleta, disponibilizado também como material suplementar.

### Análise dos dados

Após o retorno dos especialistas, aplicou-se o Índice de Validade de Conteúdo (IVC) para avaliação quantitativa^42^. O IVC mede a proporção de especialistas que concordam sobre aspectos dos itens^43^, com base na escala Likert de 1 a 4 descrita^36^. Itens com notas 1 ou 2 devem ser revistos ou excluídos. O IVC de cada item é calculado pela razão entre respostas 3 e 4 e o total de respostas, sendo aceitável um índice ≥ 0,80, preferencialmente > 0,90^44^.

Para a confiabilidade, analisaram-se variáveis categóricas por meio da concordância percentual (CP^45^), *kappa* e *kappa* ajustado para prevalência e viés (PABAK^46^) e variáveis contínuas (variedade, tamanho e preços) pelo Coeficiente de Correlação Intraclasse (CCI^47^). O PABAK foi incluído como medida complementar, por corrigir distorções em situações de prevalência extrema, conferindo maior robustez à interpretação da concordância. Os valores de CP, *kappa*, PABAK e CCI foram classificados de acordo com o critério de Byrt^38^ em: concordância excelente (≥0,92), concordância muito boa (0,80 a 0,91), concordância boa (0,60 a 0,79), concordância razoável (0,40 a 0,59), concordância superficial (0,20 a 0,39), concordância pobre (0,00 a 0,19), nenhuma concordância (<0,00). Não foram calculados valores de *kappa* e PABAK para os itens que não eram comercializados em nenhuma cantina, pois as estatísticas não puderam ser computadas pela ausência de um ou mais níveis nas tabelas de referência cruzadas.

As análises foram realizadas com o auxílio do *software Statistical Package for the Social Sciences* (SPSS 21)^48^ e do *Software for Statistics and Data Science* (Stata/SE 15)^49^. Este estudo obedeceu aos preceitos éticos da Declaração de Helsinki e das Resoluções n^o^ 466/2012 e 510/2016 do Conselho Nacional de Saúde, sendo aprovado pelo Comitê de Ética em Pesquisa da Universidade Federal de Minas Gerais (parecer número 5.240.459). Participaram do estudo especialistas e gestores/as de cantinas que consentiram em participar e registraram o aceite no termo de consentimento livre e esclarecido.

## Declaração de Disponibilidade de Dados:

Todo o conjunto de dados que dá suporte aos resultados deste estudo foi publicado no artigo e na seção “Materiais suplementares”.

## RESULTADOS

### Avaliação por especialistas

83,3% (n=10) dos especialistas convidados participaram da validação de conteúdo. A concordância foi satisfatória, com média do IVC de 0,9. As médias por seção estão na Tabela 1 do material suplementar.

Com base na avaliação, os itens foram revisados e ajustados conforme as sugestões pertinentes, discutidas pelos coordenadores do estudo. As principais alterações foram: inclusão da pergunta sobre a existência da estratégia de venda, presente na dimensão “promoção de alimentos”; inclusão do item sobre a existência de oferta de refeição e seu preço na cantina; retirada de quatro itens referentes à identificação da cantina; e reorganização da dimensão do tamanho e preço dos alimentos e bebidas - anteriormente o objetivo era conhecer o tamanho único (quando houvesse somente uma opção do alimento/bebida), mínimo e máximo, bem como o preço único (quando houvesse somente uma opção do alimento/bebida), mínimo e máximo. A versão final contou com o tamanho do alimento de menor preço e o menor preço. A dimensão da publicidade dos alimentos sofreu uma reorganização e passou a ser o objetivo da terceira seção.

Os especialistas recomendaram a elaboração de um manual de coleta abrangente, com orientações sobre aferição da variedade, unidade de medida (peso, volume ou unidade) e exemplos para a classificação dos alimentos quanto ao seu grau de processamento (conforme o manual de coleta de dados). As alterações estão descritas na Tabela 1 do material suplementar.

### Pré-teste

O pré-teste mostrou que os itens foram compreendidos e preenchidos adequadamente, sem necessidade de alterações. A sequência das seções mostrou-se pertinente e o conteúdo, claro. Então, o instrumento foi programado em um aplicativo, com itens configurados de forma dinâmica e condicionados às respostas anteriores, o que trouxe maior praticidade e reduziu erros de digitação.

### Versão final do instrumento

A versão final do instrumento elaborado considerou as dimensões: informação alimentar e nutricional, infraestrutura para alimentação, conveniência, acessibilidade financeira, disponibilidade, variedade e promoção. Contemplou 945 itens distribuídos em três seções:

1. Identificação e caracterização da cantina;
2. Comercialização, variedade, tamanho, valor do menor preço e estratégia de venda de alimentos, bebidas e preparações culinárias; e
3. Presença de publicidade desses itens.

A primeira seção foi composta de 95 itens relacionados a: aspectos administrativos, presença de nutricionista, tipo de refeições, local de preparo, disponibilidade de cardápios e informações nutricionais, estrutura para consumo, comercialização de preparações destinadas a dietas especiais (por exemplo: alergias, intolerâncias, diabetes), disponibilidade de condimentos, critérios e proibições de alimentos, incentivos de fornecedores, ações e materiais educativos sobre alimentação saudável, estratégias de preço e venda de alimentos realizada por outros membros da comunidade escolar fora da cantina.

A segunda seção foi composta de 300 itens que levam em conta a presença, a variedade, o tamanho do alimento de menor preço, o menor preço e as estratégias de venda (como combos ou promoções) para 50 alimentos e bebidas. Destes, 21 são alimentos *in natura*, minimamente processados ou processados e preparações culinárias baseadas nestes alimentos, e 29 são alimentos ultraprocessados e preparações culinárias baseadas nesses alimentos.

A terceira e última seção diz respeito às possíveis estratégias de comunicação mercadológica dos 50 alimentos e bebidas comercializados e contém 550 itens. Verifica 11 tipos de estratégias publicitárias como *banners*, vestimentas, réplicas, cardápios, entre outros. O instrumento completo está disponível no material suplementar.

### Confiabilidade do instrumento

Participaram da coleta 14 cantinas, com duração média das entrevistas de 30 minutos em escolas particulares e 20 minutos em públicas. O instrumento foi reaplicado em média após 21 dias, com intervalo de 19 a 24 dias. Na seção 1, não foi possível calcular *kappa*, PABAK e CCI para 33 itens em razão da ausência de um ou mais níveis nas tabelas de referência cruzadas, dado o baixo número de ocorrências. O mesmo ocorreu na seção 2, com 124 itens. Além disso, 50 itens não foram contemplados pela amostra, pois dez alimentos do instrumento não estavam disponíveis nas cantinas, impossibilitando a avaliação de variedade, tamanho, preço, combos e promoções. Na seção 3, nenhum item permitiu o cálculo dessas medidas, porém o percentual de concordância no teste interobservador e intraobservador foi excelente (100%). Assim, no total, foi possível avaliar a confiabilidade de 188 itens.

No desempenho geral, 95,2% do total dos itens analisados (n=188) apresentaram concordância percentual excelente, muito boa ou boa. No teste interobservador, considerando-se as análises para as variáveis categóricas, entre os 112 itens avaliados, 90,1% tiveram concordância excelente e 9,9% muito boa ou boa, para o *kappa* (variação de 0,75-1,00). Para o PABAK 89,3% dos itens tiveram concordância excelente e 9,8% muito boa ou boa. A concordância nula foi observada em apenas 0,9% dos itens, especificamente na análise do PABAK no item sobre a comercialização do suco 100% integral em caixinha, lata ou garrafa (variação de -0,28 a 1,00) (Tabela 1). O CCI calculado para os itens “quantidade clientes atendidos por dia” e para a variedade, tamanho e preço dos alimentos/refeição na análise interobservador apresentou variação de 0,0 a 1,0 (92,1%≥0,92; 2,6% entre 0,6 a 0,91; 1,3% entre 0,2 a 0,39 e 3,9%<0,0). Do total de itens avaliados (n=76), 92,1% apresentaram concordância excelente e 2,6% concordância muito boa ou boa (Tabela 2). Apenas 1,3% dos itens apresentaram concordância superficial (variedade da pizza com recheio ultraprocessado) e 3,9% dos itens apresentaram nenhuma concordância (variedade do doce de frutas; tamanho da fruta fresca e variedade do sanduíche com recheio ultraprocessado).

**Tabela 1.** Confiabilidade interobservador (*kappa* e *kappa* ajustado para prevalência e viés do observador) das seções do instrumento de avaliação da comercialização de alimentos e bebidas em cantinas escolares.

Seções	Número de itens considerados para o cálculo de kappa*	**% de itens segundo concordância (*kappa*)** ^†^			% de itens segundo concordância (PABAK)^‡^			
		E	MB	B	E	MB	B	N
Seção 1: Identificação e caracterização da cantina	59	93,2	5,1	1,7	93,2	6,8	-	-
Seção 2: Alimentos comercializados na cantina	53	86,8	9,4	3,7	84,9	11,3	1,8	1,8

*Dos 345 itens que compõem o instrumento (seção 1=95 itens e seção 2=250 itens), 123 itens referem-se às variáveis quantitativas (variedade, tamanho e preço), não se aplicando o cálculo de *kappa* e PABAK. Dos 222 itens para os quais caberia este cálculo, em 110 as estatísticas não puderam ser computadas pela ausência de um ou mais níveis nas tabelas de referência cruzadas (33 na seção 1 e 77 na seção 2); ^†^As categorias “razoável”, “superficial”, “pobre” e “nula” não foram encontradas; ^‡^Categorias “razoável”, “superficial” e “pobre” não foram encontradas.

E: excelente; MB: muito boa; B: boa; N: nula.

**Tabela 2.** Confiabilidade interobservador e intraobservador (coeficiente de correlação intraclasse) das seções do instrumento de instrumento de avaliação da comercialização de alimentos e bebidas em cantinas escolares.

Seções	Número de itens considerados para o cálculo do CCI*	% de itens segundo CCI Teste interobservador^†^					Número de itens considerados para o cálculo do CCI*	% de itens segundo CCI Teste intraobservador^‡^	
		E	MB	B	S	N		E	B
Seção 1: Identificação e caracterização da cantina	3	100	0,0	0,0	0,0	0,0	3	100	0,0
Seção 2: Alimentos comercializados na cantina	73	91,7	1,3	1,3	1,3	4,1	73	98,6	1,4

*Na seção 2, dos 120 itens que caberia esse cálculo, em 47 deles as estatísticas não puderam ser computadas pela ausência de um ou mais níveis nas tabelas de referência cruzadas; ^†^A categoria “razoável” não foi encontrada; ^‡^As categorias “muito boa”, “razoável”, “superficial”, “pobre” e “nula” não foram encontradas;

CCI: Coeficiente de Correlação Intraclasse; E: excelente; MB: muito boa; B: boa; S: superficial; N: nula.

No teste intraobservador, para os 76 itens em que o CCI foi calculado, 97,3% apresentaram concordância excelente, 2,7% apresentaram concordância muito boa e boa (variação de 0,61 a 1,00) e nenhum item teve concordância razoável, superficial, pobre nem nula (Tabela 2). Considerando-se as análises com as variáveis categóricas, do total dos 112 itens avaliados para o *kappa*, 95,5% apresentaram concordância excelente (≥0,92), 3,6% concordância muito boa e boa (CCI entre 0,6 a 0,91) e 0,9% nenhuma concordância (CCI<0,0). Com relação ao PABAK, encontraram-se 94,6% dos itens apresentando concordância excelente e 5,4% concordância muito boa ou boa, com variação de 0,8 a 1,0 (Tabela 3). Considerando-se o *kappa*, observou-se nenhuma concordância em 0,9% dos itens, especificamente no item que avalia a permissão de acordo com legislação municipal ou estadual como critério de comercialização dos alimentos. O detalhamento da avaliação encontra-se nas Tabelas 2, 3 e 4 do material suplementar.

**Tabela 3.** Confiabilidade intraobservador (*kappa* e *kappa* ajustado para prevalência e viés do observador) das seções do instrumento de instrumento de avaliação da comercialização de alimentos e bebidas em cantinas escolares.

Seções	Número de itens considerados para o cálculo de kappa*	**% de itens segundo concordância (*kappa*)** ^†^				% de itens segundo concordância (PABAK)^‡^	
		E	MB	B	P	E	MB
Seção 1: Identificação e caracterização da cantina	59	96,6	1,7		1,7	96,6	3,4
Seção 2: Alimentos comercializados na cantina	53	94,3	-	5,7		92,4	7,6

*Dos 345 itens que compõem o instrumento (seção 1=95 itens e seção 2=250 itens), 123 itens referem-se as variáveis quantitativas (variedade, tamanho e preço), não se aplicando o cálculo de *kappa* e PABAK. Dos 222 itens para os quais caberia este cálculo, em 110 as estatísticas não puderam ser computadas pela ausência de um ou mais níveis nas tabelas de referência cruzadas (33 na seção 1 e 77 na seção 2); ^†^As categorias “razoável”, “superficial” e “nula” não foram encontradas; ^‡^Categorias “boa”, “razoável”, “superficial”, “pobre” e “nula” não foram encontradas. E:excelente; MB: muito boa; B: boa; P: pobre.

## DISCUSSÃO

Este estudo elaborou, verificou a validade de conteúdo e a confiabilidade de um instrumento de avaliação da comercialização de alimentos e bebidas em cantinas escolares. Nos contextos estudados, o instrumento apresentou validade de conteúdo e confiabilidade satisfatórias, podendo ser utilizado em estudos sobre comercialização de alimentos em cantinas escolares.

A identificação e caracterização das cantinas permite traçar o perfil desses estabelecimentos e entender os alimentos comercializados nesse importante espaço organizacional. Estudos que elaboram instrumentos para avaliar ambientes alimentares organizam os itens de forma detalhada, assegurando que cada aspecto a ser avaliado esteja identificado e que as dimensões do ambiente alimentar sejam claramente definidas^28^ ^,^ ^29^ ^,^ ^30^. Os alimentos presentes no instrumento foram selecionados com base em pesquisas nacionais e dos alimentos mais frequentemente comercializados nas cantinas^6^ ^,^ ^50^ ^,^ ^51^, levando-se em consideração a classificação NOVA^52^ e as atuais diretrizes do Guia Alimentar para a População Brasileira^32^. Informações sobre variedade, preço, estratégias de venda e publicidade dos alimentos comercializados permitem uma avaliação detalhada do ambiente alimentar escolar^4^ ^,^ ^53^, analisando os obstáculos e facilitadores que crianças e adolescentes enfrentam ao tomar decisões nesses espaços.

Apesar de mais de 90% dos itens apresentarem confiabilidade satisfatória, alguns poucos itens não foram bem avaliados. Entre eles, destacam-se aqueles relacionados à comercialização do suco 100% integral em caixinha, lata ou garrafa; à variedade de pizzas com recheio ultraprocessado; à variedade de doces de frutas; ao tamanho da fruta fresca; à variedade de sanduíches com recheio ultraprocessado; e ao critério de permissão para comercialização de alimentos com base na legislação municipal ou estadual, cuja confiabilidade insatisfatória pode ser explicada pelas diferenças na interpretação e aplicação dessas legislações entre escolas, um problema que ressalta a inconsistência na implementação das regras.

No teste interobservador, as diferenças podem ser atribuídas à interpretação dos critérios pelos avaliadores, à falta de clareza ou ambiguidade nas instruções ou a variações na observação e registro dos dados, indicando a necessidade de aprimoramento do treinamento realizado. Outros estudos também encontraram esse cenário^30^ ^,^ ^54^ ^,^ ^55^, o que demonstra a complexidade de aferição de diferentes dimensões do ambiente alimentar, relacionada às características dos itens, que podem envolver subjetividade na mensuração ou apresentar variação na disponibilidade de itens.

No teste intraobservador os resultados foram ainda mais satisfatórios. As diferenças podem ser atribuídas a mudanças no ambiente, como a disponibilidade e variedade de alimentos, ou ao comportamento dos participantes entre as duas aplicações do teste. No estudo de Franco et al.^30^, cujo objetivo foi avaliar a validade de conteúdo e a confiabilidade de um instrumento para avaliação do ambiente alimentar universitário brasileiro, as diferenças encontradas deram-se pelas variações do ambiente, especificamente na oferta dos alimentos. Borges et al.^29^, ao avaliarem a confiabilidade de um instrumento de auditoria do ambiente alimentar do consumidor, encontraram pequenas mudanças na disponibilidade, preço, quantidade de marcas e estratégias publicitárias dos alimentos. É importante mencionar que o referido estudo avaliou estabelecimentos como supermercados, hipermercados e mercados, que apresentam maior variação de preço, marcas e publicidades. Isso difere do presente estudo e do estudo de Franco et al.^30^, que analisaram estabelecimentos como cantinas escolares e lanchonetes/restaurantes universitários, ambos inseridos no ambiente organizacional, onde a variedade do cardápio não costuma sofrer tantas alterações e os reajustes de preços costumam ser realizados com menor frequência, em momentos mais pontuais como as voltas às aulas.

Ainda com relação ao teste intraobservador, o estudo que avaliou a validade e a confiabilidade do instrumento *Perceived Nutrition Environment Measures Survey* (NEMS-P)^56^, traduzido e adaptado para o Brasil, também encontrou valores de CCI aceitáveis. Entretanto, assim como observado no presente estudo, algumas dimensões apresentaram desempenho inferior - no caso do NEMS-P, questões relativas a preços, *marketing* em supermercados e promoção de alimentos, que chegaram a apresentar valores baixos ou até negativos. Os autores atribuíram esses resultados ao contexto de inflação no país, às dificuldades de compreensão das questões e às limitações dos métodos psicométricos para captar percepções individuais^56^.

Uma revisão sistemática^57^ que objetivou classificar e avaliar a qualidade dos métodos de aferição do ambiente alimentar escolar identificou que poucos estudos realizaram as etapas de elaboração e avaliação da validade de conteúdo e da confiabilidade dos instrumentos^57^. Ressalta-se o ineditismo do presente estudo ao realizar todas essas etapas para um instrumento específico para mensuração da comercialização de alimentos em cantinas escolares.

Entre os pontos fortes do estudo, tem-se a validação de conteúdo por meio da análise qualitativa e quantitativa e a realização da avaliação da confiabilidade do instrumento em escolas públicas e privadas, abrangendo, assim, os dois contextos do ambiente alimentar escolar. Além disso, o instrumento incluiu uma lista ampla de alimentos, baseada na classificação NOVA, e incorporou informações detalhadas sobre a comercialização de alimentos nas cantinas. O instrumento inclui perguntas que identificam a influência da indústria alimentícia nas escolas, permitindo uma análise mais crítica desse contexto.

Entre as limitações, destaca-se o período de coleta de dados, que durou três meses, não permitindo a identificação de diferenças sazonais. No entanto, o estudo contemplou duas cidades com características socioeconômicas e demográficas distintas, o que agrega valor à análise. Para alguns dos itens para os quais caberia cálculo de *kappa* e PABAK, estas estatísticas não foram computadas, o que pode estar relacionado ao número reduzido de cantinas avaliadas, embora o tamanho da amostra tenha apresentado poder estatístico suficiente para as análises. Ademais, foram avaliadas cantinas de escolas públicas, onde a comercialização de alimentos é menos frequente, em consequência do Programa Nacional de Alimentação Escolar. Nessas escolas, limitações de recursos humanos impactam a estrutura das cantinas, resultando em alto número de respostas negativas na aplicação do instrumento.

No contexto das escolas estudadas, o instrumento demonstrou ser confiável para mensurar a comercialização de alimentos em cantinas escolares, indicando aplicabilidade em contextos similares. Sua utilização contribui para o avanço na aferição do ambiente alimentar dessas cantinas, permitindo um monitoramento detalhado e fornecendo subsídios para intervenções que promovam ambientes mais saudáveis e alimentação adequada para os estudantes.

## Materiais suplmentares

Material suplementar 1

Material suplementar 2
